# Liriopogons (Genera *Ophiopogon* and *Liriope*, Asparagaceae): A Critical Review of the Phytochemical and Pharmacological Research

**DOI:** 10.3389/fphar.2021.769929

**Published:** 2021-12-03

**Authors:** Feiyi Lei, Caroline S. Weckerle, Michael Heinrich

**Affiliations:** ^1^ Department of Systematic and Evolutionary Botany, University of Zurich, Zurich, Switzerland; ^2^ Research Group ‘Pharmacognosy and Phytotherapy’, UCL School of Pharmacy, University of London, London, United Kingdom

**Keywords:** ophiopogon, liriope, liriopogons, phytochemistry, pharmacology, critical review

## Abstract

The closely related genera *Liriope* and *Ophiopogon* (Asparagaceae), collectively known in English as liriopogons, have similar therapeutic uses in treating cough, rheumatoid arthritis, and cleaning heat. The main aim of this review is to understand the current phytochemical and pharmacological knowledge including an assessment of the quality of the scientific evidence. A literature search was conducted in line with PRISMA guidelines, by retrieving available information up to 2020 from five online resources. The bioactive metabolites of liriopogons include steroidal saponins, flavonoids, polysaccharides, organic acids, phenols. Cardiovascular protective, anti-inflammatory, anti-diabetic, anti-oxidant, anti-cancer, neuroprotective, anti-viral, anti-acute myeloid leukemia and hepatoprotective effects have been at the center of attention. From a toxicological perspective *Ophiopogon japonicus* seems to be safe. Some problems with the quality of the pharmacological evidence stand out including the application of excessive dose level and methodological problems in the design. Additionally, a reasonable link between local/traditional uses and pharmacological assessment is often vague or not reflected in the text. Future researches on liriopogons are required to use rigorous scientific approaches in research on evidence-based natural products for the future benefits of patients.

## Introduction

*Liriope* Lour. and *Ophiopogon* Ker Gawl. are two closely related genera, collectively known as liriopogons (Fantz, 1993). They comprise a total of some 84 species and are indigenous to Asia, with many species having been traditionally used as medicines in China, with the common label ‘maidong’ or ‘mai men dong’ (for the tuberous roots)- including *Ophiopogon japonicus*, together with *Liriope spicata* and *L. muscari* as alternative sources, is an example of what Linares and Bye (Linares and Bye, 1987) called plant complexes, i.e., different (and not necessarily related) species being classed under the same common name. Interestingly in this case, there is a double labelling one in Chinese but also in popular botanical nomenclature – liriopogons. According to *Shenong’s Canon on Materia Medica* (ca. 200–250 CE), maidong is categorized as upper herb to extend longevity by ameliorating heart-qi stagnation, vacuity-taxatio, and suppressing vomiting and retching. Furthermore, they are also locally used among China in treating cough, rheumatoid arthritis and cleaning heat (Huang, 1982; Zheng and Xing, 2009; CP Commission, 2020).

Different species of liriopogons exhibit similar phytopharmacological properties; they are rich in saponins, flavonoids and polysaccharides, which have been linked to relevant pharmacological activities, such as cardiovascular protective, anti-inflammatory, immunomodulatory, anti-cancer and anti-diabetic effects (Li et al., 2006; Zheng and Xing, 2009; Chen MH. et al., 2016). Recently, *Ophiopogon japonicus* (Thunb.) Ker Gawl. has been extensively used in treating COVID-19. Since the start of the COVID-19 pandemic, 31 prescriptions (including a total of 72 medicinal plants) have been recommended by the Chinese authorities, *O. japonicus* ranks as the fourth frequently used in these 31 prescriptions (Zhang and Li, 2020). Obviously, *O. japonicus*, plays a predominant role as medicinal plant among liriopogons and its phytopharmacological properties have been investigated without observing significant toxicity (Chen MH. et al., 2016). However, other species of liriopogons have received more limited scientific attention.

The combined complexity of local/traditional phytotherapeutic uses and the resulting biochemical and biomedical investigations makes this group of plants an interesting case study for a review focusing on current approaches in phytopharmacological research and to develop strategies for more robust approaches. Phytopharmacological research, as a flourishing field focusing on complex mixtures, requires as all fields of research, robust and reproducible research. Recently, editors of leading journals called for better designed and reported research, i.e., to consider and cover appropriate models, controls, dosage, reasonable link between local/traditional uses and the assay (Heinrich et al., 2020). Core to this is a greater emphasis on the characterisation of the material under study. This includes botanical, pharmacognositc, chemical as well as other methodological details. Accordingly, in this review, the core aims are to assess:

1) The species most commonly used with regards to the level of information is available on their pharmacological and chemical characteristics,
2) The chemical metabolites or extracts isolated from liriopogons,
3) The corresponding pharmacological effects of the bioactive metabolites, and
4) The rigorousness of these pharmacological studies according to good practice standards.

## Methodology

### Search Strategy

**FIGURE 1:**
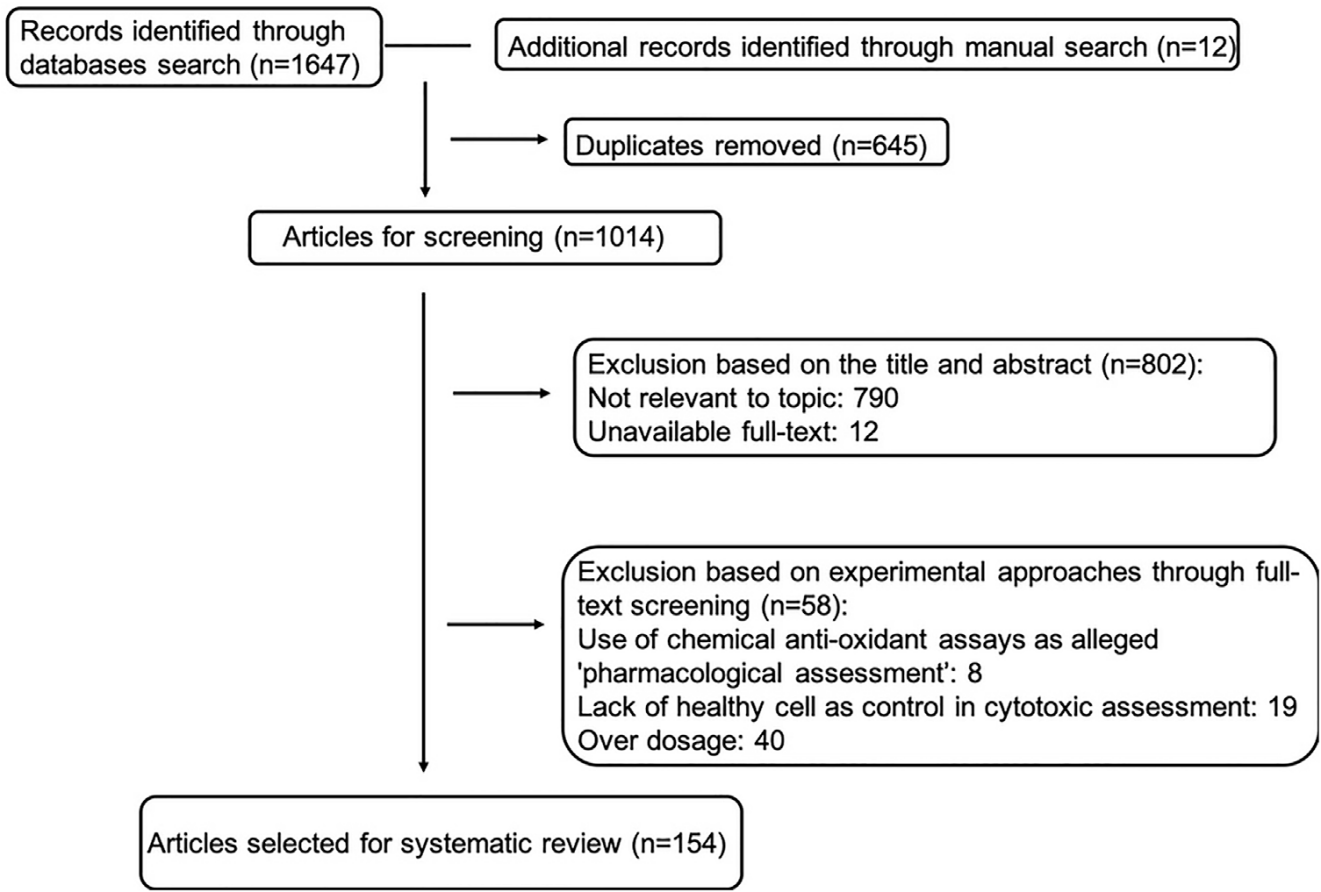
Workflow chart of inclusion and exclusion of studies in the systematic review (exclusion based on experimental approaches may overlap, therefore selected article number is slightly different from simple subtraction).

A literature search was conducted in line with PRISMA guidelines (Moher et al., 2009). Predominantly, four databases, Web of Science (core collection), PubMed, Scopus and SciFinder were consulted from inception until 2020. MeSH terms were used to identify search terms. *Ophiopogon* and *Liriope* were searched separately, for each using Boolean operators: Pharmaceutical OR Biological Activity OR Phytochemistry OR Chemical Constituent OR Pharmacology OR Phytopharmacology, respectively. Additional information was retrieved by manual searching through Google scholar. Since liriopogons have been traditionally and widely used in Chinese Medicine, publications in Chinese were considered using the database China National Knowledge Infrastructure (CNKI). Here, 麦冬 (maidong) was jointly searched with 化学成分 (phytochemistry and chemical constituent) OR 活性 (pharmaceutical and biological activity) OR药理 (pharmacology). The application of scientific names was in accordance with the World Flora Online (WFO, 2021). The workflow of our search strategy is shown in Figure 1.

### Primary Selection Criteria

Publications were included for further critical assessment if one of these two criteria was met: 1) Isolation of pure metabolites of liriopogons was reported (phytochemical publications, *n* = 106); 2) Pharmacological effects of extracts, pure metabolites (not including derivatives) were evaluated (pharmacological publications, *n* = 113). The publications retrieved are summarized in Supplementary Table S6.

### Terminology

Since the terminology especially as it relates to the botanical drugs is often not precise or misleading, we standardized the terms used for plant parts as follows- standardized terminology (original sources):

- Tuberous root (tuber)
- Tuberous root (rhizome)
- Tuberous root (tuberous root)
- Fibrous root (fibrous root)
- Subterranean part (underground part)
- Subterranean part (subterranean part)
- Subterranean part (root)
- Aerial part (aerial part)
- Whole plant (whole plant)
- Fruit (fruit, seed)
- Stalk (stalk)

### Critical Review of Pharmacological Publications

For the literature analysis we critically assessed the experimental approaches used, following Heinrich et al. (2020). Specifically, we looked at the dosage, antioxidant models, controls (esp. cytotoxic findings); additionally, methodological details were taken into consideration (Table 1). Table 3 (derived from Supplementary Table S6 with all publications before assessment) includes the pharmacological publications included in the analysis after assessment.

**TABLE 1:** Criteria for the exclusion of studies which are considered to be of limited relevance in a pharmacological context (based on Heinrich et al., 2020).

Category	Concerns	Critique
Experimental approaches	Antioxidant models	No therapeutic benefits can be deduced from chemical antioxidant assessment like the DPPH or ABTS assay
	Dosage range	Results based on doses/concentrations higher than what can be achieved in humans do not provide therapeutic value
		- For extracts, the dose range should not exceed 100–200 mg/kg (p.o.) for *in vivo* studies, and 100–200 μg/ml was considered as being the upper limit for *in vitro* studies
		- For pure metabolites, the upper limit dose should be even lower, ca. 50 mg/kg for *in vivo* studies (p.o.) and of 30–50 μM for *in vitro* studies
	Appropriate controls	- Generally, check whether appropriate controls were included
		- Specifically, for cytotoxic findings there should be a comparison of the effect on tumor and healthy cells
Methodological details	Composition	Sufficient details on the extract are needed, e.g., at least the drug: extract ratio, and a clear indication of the solvents and type of extraction
	General	Is there a reasonable link between local/traditional uses and the pharmacological assessment?

## Phytochemistry

**FIGURE 2:**
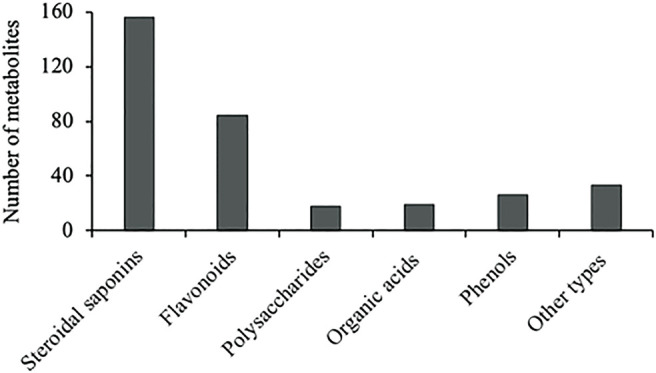
Major classes of secondary metabolites isolated from liriopogons.

Various metabolites have been isolated and characterized from different parts (tuberous roots, fibrous roots and aerial parts) of liriopogons, including steroidal saponins (Supplementary Table S1), flavonoids (Supplementary Table S2), polysaccharides (Supplementary Table S3), phenols and organic acids (Supplementary Table S4) and other types of metabolites (Supplementary Table S5). Steroidal saponins are a core group of secondary metabolites of liriopogons, followed by flavonoids (Figure 2). Chemical structures of pharmacologically active metabolites are shown in Table 2. As main bioactive metabolites, the chemical structures of steroidal saponins and flavonoids are shown in Supplementary Tables S1, S2 and Supplementary Figure S2.

**TABLE 2:** Chemical structures of pharmacologically investigated metabolites with corresponding activities of liriopogons (details of activities can be found in Table 3).

Metabolite	Chemical structures	Investigated pharmacological activity
Ruscogenin	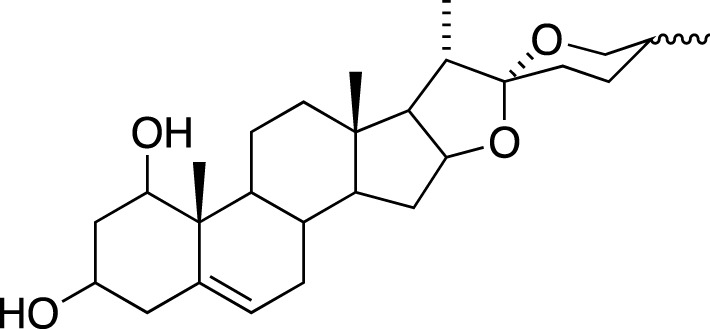	Cardiovascular protective, anti-inflammatory, effects on the endocrine system, Immunomodulation, anti-cancer
Ophiopogonin D	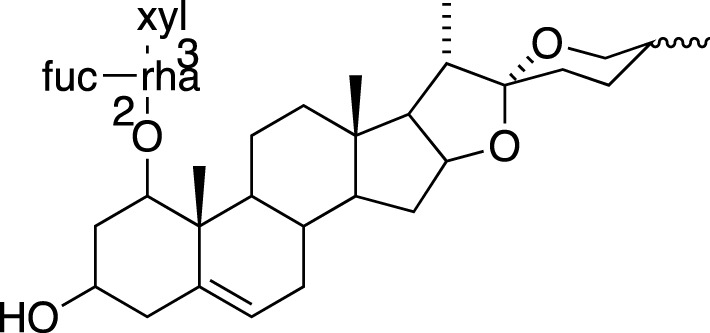	Cardiovascular protection, anti-inflammation, effects on the endocrine system, anti-oxidation, cytotoxicity, anticancer, anti-tussive
Ophiopogonin D′	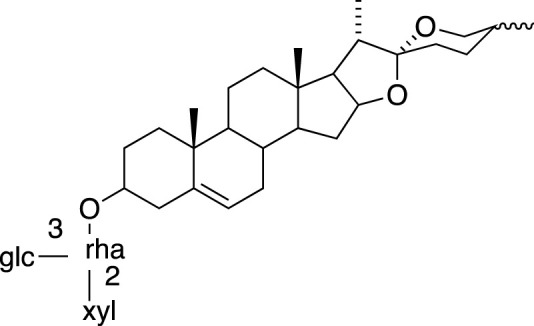	Cytotoxicity, anti-cancer
DT-13	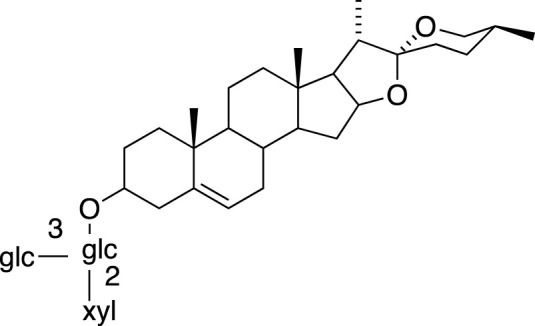	Carddiovascular protection, anti-cancer, immunomodulation, cytotoxicity, anti-cancer, anti- acute myeloid leukemia
Sprengerinin C	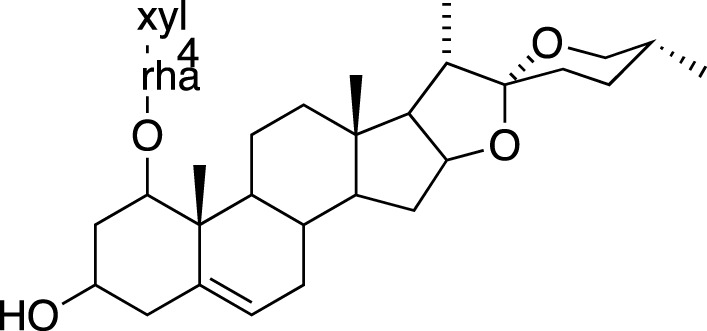	Anti-cancer
Diosgenin-3-O-[2-O-acetyl-α-l-rhamnopyranosyl-(1→2)][β-d-xylopyranosyl-(1→4)]-β-d-glucopyranoside (Metabolite 26)	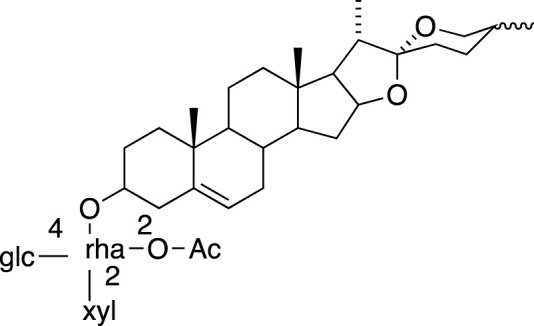	Anti-cancer
Ophiopogon saponin C1	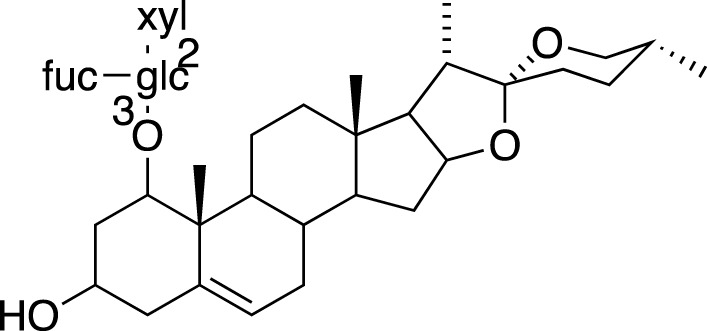	Anti-cancer
Spicatoside A	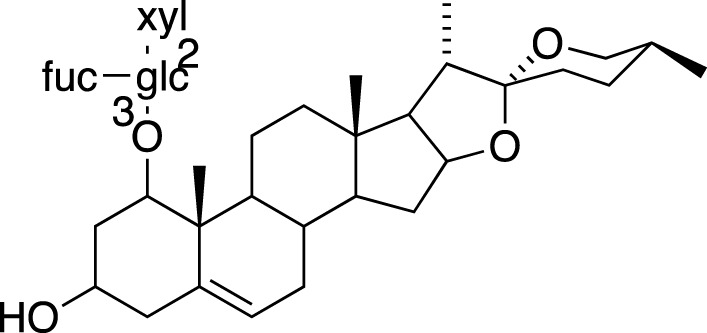	Anti-inflammation, anti-viral
Methylophiopogonone A	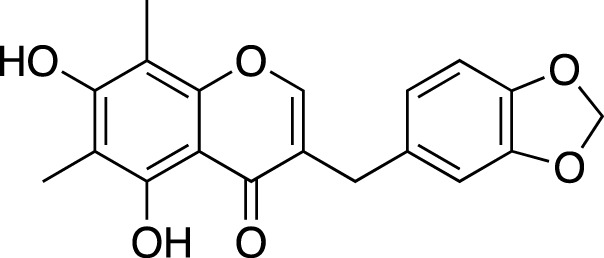	Anti-inflammation
Methylophiopogonanone A (MONA)	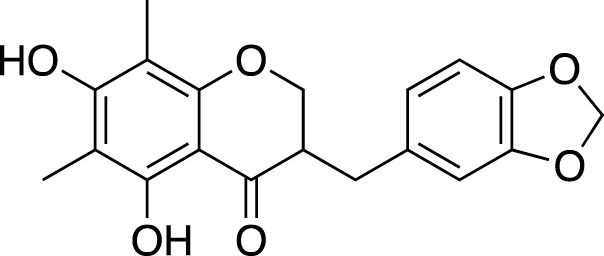	Cardiovascular protection, effects on the endocrine system
Methylophiopogonanone B	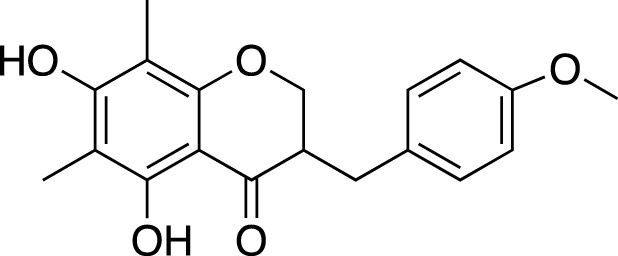	Cardiovascular protection, anti-inflammation, cytotoxicity
(3R)-3-(2′,4′-dihydroxybenzyl)-5,7-dihydroxychroman-4-one	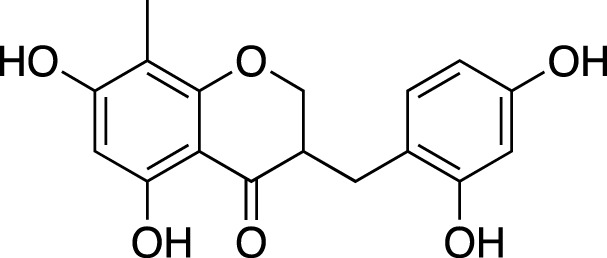	Cardiovascular protection
(Metabolite 209)		
(3R)-3-(2′,4′-dihydroxybenzyl)-5,7-dihydroxy-6-methyl-chroman-4-one	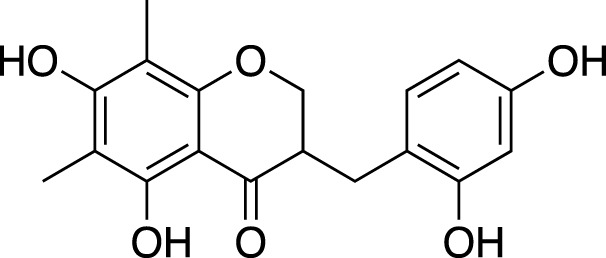	Cardiovascular protection
(Metabolite 210)		
4′-O-Demethylophiopogonnaone E	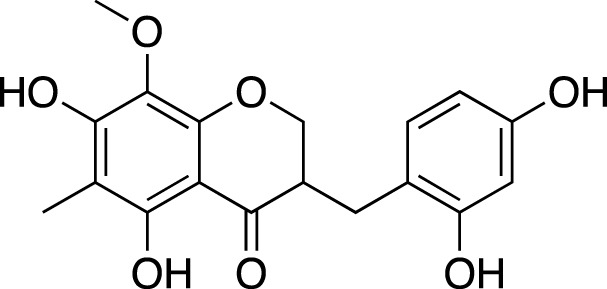	Anti-inflammation
Ophiopogonone E	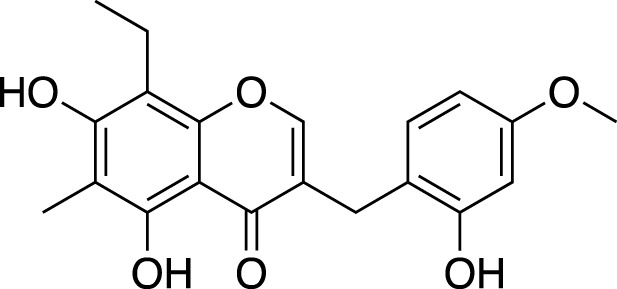	Anti-inflammation
Ophiopogonanone H	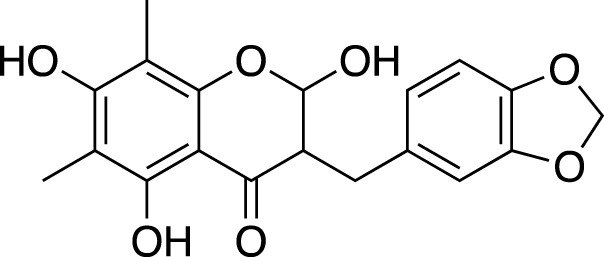	Anti-inflammation
(2R)-(4-methoxybenzyl)-5,7-dimethyl-6-hydroxyl-2,3-dihydrobenzofuran	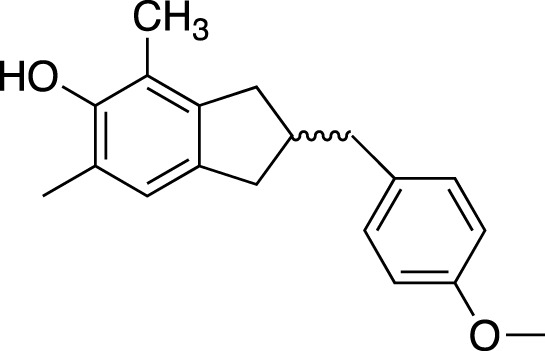	Anti-inflammation
2-(2-hydroxyl-4-methoxy-benzyl)-5-methyl-6-methoxyl-2,3-dihydrobenzofuran	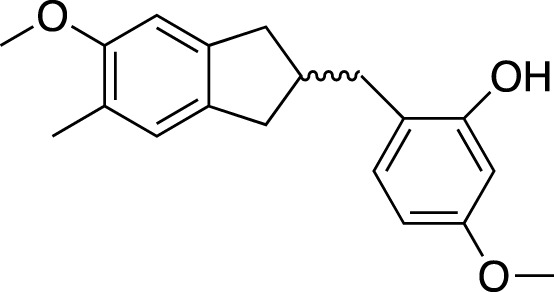	Anti-inflammation
8-formylophiopogonanone B (FOB-8)	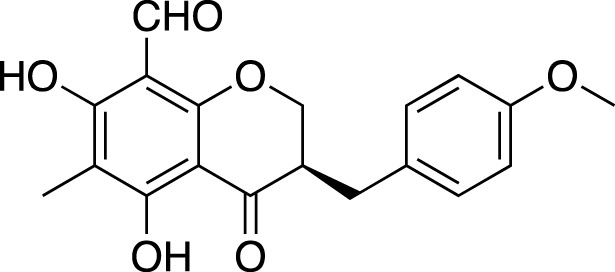	Anti-oxidation
(3R)-3-(4′-hydroxybenzyl)-5,7-dihydroxy-6-methyl-chroman-4-one (Metabolite 207)	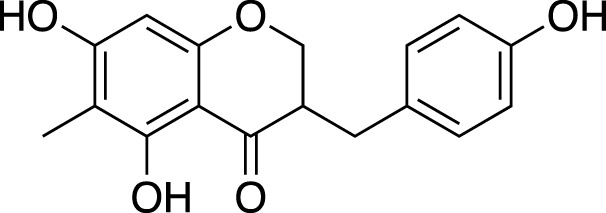	Anti-viral
58-F	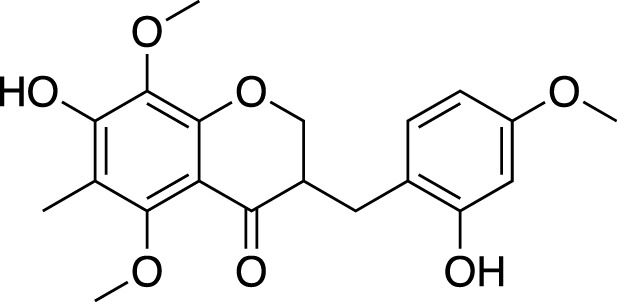	Hepatoprotection
oleic acid	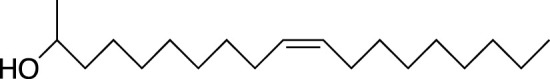	Cardiovascular protective, anti-inflammatory, effects on the endocrine system, immunomodulation, anti-cancer
syringic acid	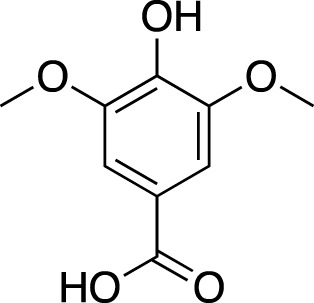	Cardiovascular protection, anti-inflammation, effects on the endocrine system, anti-oxidation, cytotoxicity, anti-cancer, anti-tussive effect

*Ophiopogon japonicus, Liriope muscari* and *L. spicata* have been well studied phytochemically, while less attention paid to other species. Overall, 337 metabolites have been isolated from liriopogons, only three metabolites have been reported each from *Ophiopogon planiscapus* Nakai and *Ophiopogon jaburan* (Siebold) Lodd.*,* one from *Ophiopogon intermedius* D. Don and 15 from *Lirioipe graminifolia* (Drude) Becc., the rest of them are found in *O. japonicus, L. muscari* and *L. spicata*.

### Steroidal Saponins

The absolute configurations of many steroidal saponins have been comprehensively determined by 1D, 2D, NMR, CD and MS spectral data analysis. Overall, so far, 156 steroidal saponins have been isolated and characterized from liriopogons from tuberous and fibrous roots, including 88 from *O. japonicus*; 37 from *L. muscari*; 31 from *L. spicata*; nine from *L. graminifolia*; two from *O. jaburan*; five from *O. planiscapus*; one from *O. intermedius* (Rawat et al., 1988). In addition, metabolites 53, 57, 58 (DT-13),71 (Ophiopogon A), 72 (Ophiopogon B) and 76 (Ophiopogon D) have been isolated both from *Ophiopogon* and *Liriope* spp., and most of them have been further evaluated focusing on a broad spectrum of bioactivities.

### Flavonoids

Generally, flavonoids including flavanones, isoflavanones and homoisoflavanones have been reported mainly from tuberous roots and fibrous roots of liriopogons, a few are from the aerial parts and fruits (Lee and Choung, 2011; Tsai et al., 2015). Flavonoids isolated from *Ophiopogon* spp. are mainly homoisoflavones; whereas, the diversity of flavones and isoflavones is higher in *Liriope* spp., together with several homoisoflavones.

In total, 84 flavonoids have been isolated from liriopogons, including 47 from *O. japonicus*; 29 from *L. muscari*; six from *L. graminifolia*; one from *O. jaburan*. Only, methylophiopogonanone B was isolated both from *O. japonicus* and *L. graminfolia*.

### Polysaccharides

Polysaccharides in the tuberous roots of *L. spicata*, *L. muscari* and *O. japonicus* have been evaluated reaching 53.2%, 54.7 and 55.2%, respectively, and their structures were distinctively different kim (Gong et al., 2017). In total, 18 polysaccharides have been isolated and identified from liriopogons.

#### Others

Other metabolites have also been isolated from the tuberous roots, fibrous roots and aerial part of liriopogons, including 19 organic acids (metabolite 260–278, 26 phenols (metabolite 279–304), 13 glycosides (metabolite 305–317) and 20 other types of metabolites (metabolite 318–337). Among them, three organic acids existed both in *Ophiopogon* and *Liriope* spp. Vanillic acid were found existing in *O. japonicus*, *L. spicata* and *L. muscari*. Oleanolic acid and palmitic acid have been both isolated from *O. japonicus* and *L. muscari*.

## Pharmacological Properties of Liriopogons

Several species of liriopogons have been used in local/traditional medicines in South-East Asia, and are especially popular within China. Although only four species of liriopogons have been pharmacologically investigated *in vitro* or *in vivo*, a variety of pharmacological properties have been reported including anti-inflammatory, immunomodulatory, antioxidant, anti-cancer, anti-tussive, neuroprotective, anti-viral activities, and the effects on the cardiovascular and endocrine system. All pharmacological findings performed with extracts and pure metabolites isolated from liriopogons together with an overview of tested species (with main focus on *O. japonicus, L. muscari and L. spicata*) for each pharmacological effect are summarized in Table 3. Generally, *O. japonicus* represents the most important medicinal species of liriopogons and has been widely studied both *in vitro* and *in vivo*, exhibiting anti-inflammatory, immunomodulatory, antioxidant, anti-cancer, and anti-tussive activities, as well as the effects on the cardiovascular and endocrine system. In addition to neuroprotective, anti-infective and hepatoprotective effects, *Liriope muscari* possesses similar pharmacological activities as *O. japonicus* does (except anti-oxidative, anti-tussive and immunomodulatory activities). *Liriope spicata* exhibits the effects on the endocrine system and inflammatory diseases. Meanwhile, the cytotoxic effects of liriopogons on various tumor cells and traditional therapeutic effects of *L. muscari* on dry eye syndrome, gastrointestinal motility and bronchial asthma have also been investigated (Kim et al., 2016; Lee et al., 2019; Song et al., 2019). During the search we did not identify any clinical studies of relevance.

**TABLE 3:** Summary of pharmacological studies on extracts/metabolites isolated from liriopogons included in this review.

Activity	Plant resource	Metabolite tested pharmacologically	Model	Effect	Dosage	References
Cardiovascular protection (this activity has been tested on *O. japonicus* and *L. muscari*)	*O. japonicus*	Steroidal saponins extract	DOX-induced SD rats	↓ values of LVEDP, LVESD and LVEDD; levels of IL-6, TNF-α, IL-1β, MDA; the relative activity of p38 MAPK ↑values of LVESP, +2dP/dtmax, –dP/dtmax, EF and FS; activities of SOD, CAT and GSH-Px	100 mg/kg (p.o.)	Wu et al. (2019)
		Aqueous extract	ICR mice SD rats	↓ length of tail thrombus ↓ arterial-venous shunt	12.5 and 25.0 mg/kg; 6.25 and 12.5 mg/kg (p.o.)	Kou et al. (2006)
		Ethanol extract	SD rats HL-60 cells and ECV304 cells	↓ the dried weight of thrombus (36.0 and 70.6%); endothelium injury, adherent or transmigrated leukocytes ↓ adhesion of HL-60 cells to ECV304 cells	12.5 and 25.0 mg/kg; (p.o) 0.1, 1.0 and 10 μg/ml	Kou et al. (2005b)
		Ruscogenin	(MCAO/R)-injured mice	↓ infarct size; brain water; ICAM-1, iNOS, COX-2, TNF-α, IL-1β; NF-κB p65 and phosphorylation ↑ neurological deficits	5 and 10 mg/kg (i.g.)	Guan et al. (2013)
		Ruscogenin	(MCAO/R)-injured mice	↓ brain infarction and edema, EB leakage ↑ neurological deficits, cerebral brain flow CBF, ameliorated histopathological damage; expression of TJs	10 mg/kg	Cao et al. (2016)
			OGD/R-injured bEnd.3 cells	↓ sodium fluorescein leakage, expression of TJs, IL-1β and caspase-1, NLRP3 and TXNIP ↑ cell viability and TEER value	0.1–10 μM	
		Ophiopogonin D	H9c2 cells ^5^C7BL/6J mice	↓ LC3-II/LC3-I ratio, activation of JNK and ERK in H9c2 cells ↓ DOX-induced cardiac dysfunction in mice	1 μM 10 mg/kg (i.p.)	Zhang et al. (2015c)
		Ophiopogonin D	H9c2 cells Ang II-induced H9c2 cells	↑ CYP2J3 expression and 14,15-DHET levels in normal H9c2 cells ↓ angiotensin II-induced abnormalities in Ca^2+^ homeostasis, ER stress	100, 250 and 500 nM	You et al. (2016)
		Ophiopogonin D	DOX-induced H9c2 cell DOX-induced rats	↓ ROS accumulation and up-regulation of ERS related proteins ↓ cardiac ultrastructural abnormalities in rats	1 μM 10 mg/kg (i.p.)	Meng et al. (2014)
		Ophiopogonin D	Ang II-infused H9c2 cells Ang II-infused rats	↓ ANP, BNP, β-MHC, *p*-IκBα, *p*-REL-A, and REL-A proteins ↑ LVESD and LVEDD	0.1, 0.25, and 0.5 μM 5 or 10 mg/kg (i.p.)	Wang et al. (2018)
		DT-13	Rat ventricular myocytes	↓ cardiac intracellular Ca^2+^ ↑ current voltage curve	0.1 μM	Tao et al. (2005)
		Methylophiopogonanone A (MONA)	MCAO-induced rats ODG/R -induced bEND.3 cells THP-1 cells	↓ infarct volume and brain edema, body weight decreases, ROS production, MMP-9 release, ICAM-1 and VCAM-1 expression ↑ neurological deficit scores, survival time, TJ	1.25, 2.50 or 5.00 mg/kg (i.v.) 2.5, 5.0 or 10 μM	Lin et al. (2015)
		Methylophiopogonanone A (MONA)	I/R-induced mice H/R-induced H9C2 cells	↓ infarct size (by 60.7%) and myocardial apoptosis (by 56.8%), cell apoptosis and cleaved caspase-3 expression ↑ cardiac function; PI3K, *p*-Akt, *p*-eNOS, Bcl-2/Bax ratio and restored NO production	10 mg/kg (p.o.) 10 μM	He et al. (2016)
		Methylophiopogonanone B (MONB)	H_2_O_2_-induced HUVECs	↓ production of MDA and ROS, H_2_O_2_-induced apoptosis, p22phox ↑ SOD activity	10, 20, 40 and 50 μM	Wang et al. (2019)
	*L. muscari*	DT-13	C57BL/6 mice HUVECs	↓ ROS, TNFR, IL-8, MCP-1 and NO (dose dependent) ↓ NO production, phosphorylation of endothelial NO synthase	4 mg/kg (i.v.) 0.01, 0.1, 1 μM	Fan et al. (2018)
		DT-13	SD rats	↓ mRNA expression levels of IL-6 and TF	1.0, 2.0 and 4.0 mg/kg (p.o.)	Tian et al. (2013)
		DT-13	HUVECs	↓ cleaved caspase-3 and cleaved PARP ↑ mitochondrial membrane potential, Akt phosphorylation	1, 2, 5 μM	Qiu et al. (2014)
		Metabolite 209 and 210 (Flavonoids)	Plates	↓ platelet aggregation at IC50 value of 11.59 and 10.69 μM	-	Tsai et al. (2013)
Anti-inflammatory effects (this activity has tested on *O. japonicus, L. muscari* and *L. spicata*)	*O. japonicus*	ROJ-ext (Aquesous extract)	ICR mice and SD rats; HL-60 and ECV304 cells	↓ ear swelling, pawedema, pleural leukocyte migration, peritoneal total leukocyte and neutrophil migration ↓ adhesion of HL-60 cells to ECV304 cells, with IC50 of 42.85 μg/ml	25 and 50 mg/kg (p.o.) -	Kou et al. (2005a)
		Ruscogenin	LPS-induced mice	↓ lung wet/dry weight ratio, LPS-induced MPO activity and nitrate/nitrite content; expression of TF, iNOS, procoagulant activity; NF-κB p-p65	0.3, 1.0 and 3.0 mg/kg (p.o.)	Sun et al. (2012)
		Ruscogenin	MCT-rats	↓ endothelial cell apoptosis ↑ eNOS, caveolin-1, and CD31	0.1, 0.4 and 0.7 mg/kg (p.o.)	Bi et al. (2013)
		Ophiopogonin D	TNF-α- inflamed HaCaT cell; DNCB-treated mice	↓ spleen/body weight ratio; TNF-α, IL-4, and IL-5; p38 and ERK protein activation and NF-κB nuclear translocation	1 and 10 μM; 125 and 250 nM	An et al. (2020)
		DT-13	HUVECs THP-1 TNF-α induced mice	↓ vascular inflammation, expression of ICAM-1 and VCAM-1; NF-кB p65 phosphorylation, p38 phosphorylation and Src degradation	0.01, 0.1and 1 μM 4 mg/kg (i.g.)	Zhang et al. (2015b)
		4′-O-Demethylophiopogonanone E	LPS-induced RAW 264.7 cell	↓ production of NO with IC50 value of 80.2 μg/ml; production of IL-1β and IL-6 with the IC50 value of 32.5 μg/ml and 13.4 μg/ml, respectively	0–50 μg/ml	Zhao et al. (2017)
		Methylophiopogonone A; Ophiopogonone E; Methylophiopogonanone B; Ophiopogonanone H	LPS-induced murine microglial cell BV-2	↓ NO production with IC50 of 19.2, 14.4, 7.8 and 20.1 μM, respectively	-	Li et al. (2012a)
		Ophiopogonanone G; Ophiopogoside A; Ophiopogoside B	human bronchial epithelial BEAS- 2B cell	↓ IL-4-induced eotaxin production and eotaxin expression	25.0 μM	Hung et al. (2010)
		MDG-1	HUVECs	↓ Bax/Bcl-2 protein ratio, caspase-3, TNF-α, IL-1β, IL-6 and Cox-2	5, 10 or 50 mM	Li et al. (2017)
		Metabolite 289; Metabolite 290 (phenols)	LPS-induced RAW 264.7 macrophage cells	↑ LPS-induced NO production in RAW264.7 cells with the IC50 value of 11.4 and 29.1 μM, respectively	-	Dang et al. (2017b)
	*L. muscari*	DT-13	Mice; HL-60/ECV304	↓ acute paw edema induced by histamine in mice; adhesion of HL-60 to ECV304 cells induced by TNF-α or PMA	4.6 mg/kg (p.o.) 0.01, 0.1 and 1 μM	Tian et al. (2011)
	*L. spicata*	Metabolite 279, 280 (phenols)	Neutrophils	↓ neutrophil respiratory burst stimulated by PMA with IC50 value of 5.96 and 4.15 μM, respectively	-	Hu et al. (2011)
Effects on the endocrine system (this activity has been tested on *O. japonicus* and *L. spicata*)	*O. japonicus*	Methylophiopogonanone A (MONA)	HFD-induced obese rat model	↓ expression of ACC and SREBP-1C ↑ activities of lipoprotein lipase and hepatic lipase in serum and liver; expression of LDLR and PPAR α	10 mg/kg (i.g.)	Li et al. (2020)
		Ruscogenin	STZ-induced diabetic rat	↓ macrophage influx; expression of TNF-α, IL-6 and IL-1β	3.0 mg/kg (p.o.)	Lu et al. (2014)
		Ophiopogonin D	HFD male mice	↓ *Firmicutes/Bacteroidetes* ratios and endotoxin-bearing *Proteobacteria* levels	1 mg/kg (i.g.)	Chen et al. (2018b)
		Ophiopogonin D	STZ-induced DN rats	↑ serum albumin and creatinine clearance, serum creatinine, blood urea nitrogen, kidney hypertrophy; TGF-β1, and, GSH, SOD, CAT ↓ MDA, IL-6, IL-1β	2.5, 5 and 10 mg/kg (p.o.)	Qiao et al. (2020)
	*L. spicata*	LSP1, LSP2	STZ-induced diabetic mice	↓ fasting blood glucose, TC, TG, LDL-C, HDL-C/TC ↑ glucose tolerance, insulin resistance	100 and 200 mg/kg (p.o.)	Chen et al. (2009a)
		Aqueous ethanol extract	STZ-diabetic rats	↓ creatinine clearance, ICAM-1, MCP-1, and fibronectin protein, TNF- α and IL-1β ↑ histological architecture, blood urea nitrogen and proteinuria	100 or 200 mg/kg (p.o.)	Lu et al. (2013)
		LSP1, LSP2	KKAy diabetic mice	↓ fasting blood glucose, lipid accumulation, hepatic gluconeogenesis ↑ insulin resistance and serum lipid metabolism, glycolysis and hepatic glycogen content; expression of InsR, IRS-1, phosphatidylinositol 3-kinase, and PPAR *γ*	100 and 200 mg/kg (i.g.)	Liu et al. (2013)
Immunomodulation (this activity has been tested on *O. japonicus* and *L. muscari*)	*O. japonicus*	Polysaccharides	C57BL/6 mouse	↓ SMG index, spleen index, IFN-γ level and IFN-γ/IL-4 ratio ↑ salivary flow, body weight; water intake	5 and 10 mg/kg (p.o.)	Wang et al. (2007)
	*L. muscari*	DT-13, ruscogenin	ICR mice; nonparenchymal cells; hepatocytes and spleen cells	↓ ALT level, hepatocelluar necrosis and adipose degeneration ↓ release of ALT innonparenchymal cells with IC50 of 6.3 × 10^–10^ M and 3.9 × 10^–7^ M, lympho proliferation	10 or 20 mg/kg (i.p.); 10^−5^–10^–4^ μM	Wu et al. (2001)
		Water extract	LPS-induced mouse	↓ NO, IL-6, IL-10, IL-12p40, IP-10, KC, MCP-1, VEGF, GM-CSF, PDGF-BB, intracellular calcium, NF-κB and CREB	25–200 μg/ml	Kim et al. (2012)
Anti-oxidation (this activity has been tested on *O. japonicus*)	*O. japonicus*	Ophiopogonin D	HUVECs	↓ H_2_O_2_-induced oxidative stress, apoptosis and ERK1/2 activation	0.6–60.0 μM	Qian et al. (2010)
		Ophiopogonin D	MC3T3-E1cells and RAW264.7 cells; BALB/c female mice	↓ induced MC3T3-E1 dysfunction, H_2_O_2_-induced MC3T3-E1 dysfunction ↓ CTX-1, TRAP activities, MDA, ROS generation, expression of β-catenin, mRNA expressions of Axin2 and OPG	1, 10,100 μM 5 and 25 mg/kg (i.p.)	Huang et al. (2015)
		8-formylophiopogonanone B (FOB-8)	PQ-induced mice	↓ PQ-induced elevation in MDA, GSH and SOD levels	20 mg/kg (i.g.)	Qian et al. (2019)
Anti-cancer (this activity has been tested on O. japonicus and *L. muscari*)	*O. japonicus*	Ophiopogonin D′	PC3 and DU145 cells (prostate cancer); BALB/c nude mice implanted with PC3 and DU145 cells	↓ levels of cleaved-RIPK1, caspase 8, cleaved-caspase 8, Bid, caspase 10, and cleaved-caspase 10 ↑ cell apoptosis, expression levels of RIPK1 and Bim ↓ PC3 and DU145 xenograft tumors in BALB/c nude mice	1, 2.5, 5, 10, 25, and 50 μM 2.5 or 5.0 mg/kg (i.p.)	Lu et al. (2018)
		DT-13	95D cells (lung cancer); Orthotopic implantation mouse model	↓ 95D cells metastasis, expression of paxillin, *p*-paxillin, p-c-Raf, total c-Raf, *p*-ERK1/2, total ERK1/2 and β-actin ↑ non-muscle myosin IIA	0.01, 0.1 and 1 μM 2.5 or 10 mg/kg (i.g.)	Wei et al. (2016)
		DT-13	HCT-15, HT-29 cells (colorectal cancer); Orthotopic implantation mouse model of colorectal cancer; C57BL/6J APC^min^ mice model	↓ glucose uptake, ATP generation, lactate production, m-TOR ↑ AMPK ↓ expression of GLUT1, colorectal cancer growth	2.5, 5 and 10 μM 0.625, 1.25, 2.5 mg/kg (i.g.) 10 mg/kg (i.g.)	Wei et al. (2019)
		Ruscogenin	SMMC-7721 and HCCLM3 (liver cancer); nude mice implanted with HCCLM3 cells	↓ cell migration and invasion; levels of MMP-2, MMP-9, urokinase-type plasminogen activator, VEGF and HIF-1α; phosphorylation of Akt, mTOR	0–100 μM; 0.3, 1.0, or 3.0 mg/kg (i.v.)	Hua et al. (2018)
		Sprengerinin C	HUVECs, HepG-2/BEL7402 cells; nude mice implanted with HepG-2 cells	↓ VEGF-induced vascular endothelial cell proliferation, invasion and tube formation; VEGFR2 activation, MMP-2/9 and VEGF expression ↑ G2/M phase arrest, NADPH oxidase activity, reactive oxygen species, cleaved caspase-3 and cleaved PARP ↓ tumor growth in a nude mouse	0.5, 1.0 and 2.0 μM; 7.5 and 15 mg/kg (i.p.)	Zeng et al. (2013)
		Metabolite 26 (saponin)	HUVECs C57/BL mice	↓ HUVECs invasion and tube formation; expression of Src tyrosine kinase ↓ angiogenesis and MMPs/VEGF expression	1.25, 2.5, 5.0 and 10.0 μM 5.0 μM (SC)	Zeng et al. (2015)
	*L. muscari*	Ophiopogon Saponin C1	A549 cells; mice	↓ cell migration ↓ degradation and breakage of the ZO-1 protein, PKCδ and Src	0.01, 0.1, 1 μM 4.0 mg/kg (i.g.)	Zhang et al. (2020)
Anti-viral	*L. muscari*	Metabolite 207 (falvonoid)	HBV-transfected Huh7 cells	↓ pCore-Luc, pS-Luc, pPreS-Luc activities; binding activity of NF- κB protein to CS1 element; CS1 containing promoter activity ↓ expression of p65/p50 NF- κB protein, phosphorylated NF-κB p65 ↑ cytoplasmic I κB αprotein levels	0–10 μg/ml	Huang et al. (2014)
		Spicatoside A	Huh 7.5 (hepatocellular carcinoma cell)	↓ replication of the genotype 3 HEV replicon ↓ HEV genotype 3 strain 47832c ↓ expression of HEV ORF2	0.5, 1 and 2 μg/ml; 2 μg/ml 0.2, 0.5, 1 and 2 μg/ml	Park et al. (2019)
Anti-tussive	*O. japonicus*	Ophiopogonin D	Paratracheal neurones	hyperpolarized the paratracheal neurones from a resting membrane potential of -65.7 to -73.5 mV	10 μM	Ishibashi et al. (2001)
Neuroprotection	*L. muscari*	Ethanol extract	H_2_O_2_-induced injury in SH-SY5Y cells (neuroblastoma cell)	↓ intracellular oxidative stress, mitochondrial dysfunction; poly (ADP ribose) polymerase and caspase-3 cleavage	0.5–50 μg/ml	Park et al. (2015)
Acute myeloid leukemia (anti-AML)	*L. muscari*	DT-13	Human leukemia cell lines; NOD/SCID mice with the engraftment of HL-60 cells	↑ apoptosis of HL-60 and Kasumi-1 cells ↑ Fas, FasL, DR5, TRAIL, the cleaved-PARP and cleaved-caspase 3 and 8, differentiation markers CD11b and CD14, level of C/EBPα and C/EBPβ ↑ NOD/SCID mice survival time	0–18 μM; 10 and 20 mg/kg (p.o.)	Wang et al. (2020a)
Hepatoprotection	*O. japonicus*	58-F	CCl_4_-induced mouse; H_2_O_2_-induced BNL CL.2 hepatocyte cell	↓ lysosome membrane permeabilization, cathepsin B, cathepsin D ↑ lysosomal enzyme translocation to the cytosol, fluorescence intensity of the LysoTracker Green, cell viability	15 mg/kg (i.g.) 50 μM	Yan et al. (2016)

**Abbreviations**: ABTS, 2,2′-azino-bis (3-ethylbenzthiazoline-6-sulfonicacid); ACC, acetyl CoA carboxylase; AMPK, adenosine 5′-monophosphate (AMP)-activated protein kinase; Ang II, Angiotensin II; ANP, atrial natriuretic peptide; ALT, alanine transaminase; AST, aspartate transaminase; BBB, blood brain barrier; BBMV, intestinal brush border membrane vesicles; bFGF, basic fibroblast growth factor; BNP, B-type natriuretic peptide; CREB, cyclic adenosine monophosphate response element-binding protein; CAT, catalase; CBF, cerebral flow; CCR3, C-C motif chemokine receptor 3; CHF, chronic heart failure; COX-2, cyclooxygenase; CTGF, connective tissue growth factor; DN, diabetic nephropathy; DPPH, 2,2-diphenyl-1-picrylhydrazyl; DT-13, 25 (R,S)-ruscogenin1-O-[β-d-glucopyranosyl-(1→2)]-[β-d-xylopyranosyl-(1→3)]-β-d-fucopyranoside; EB, evans blue; EETs, epoxyeicosatrienoic acids; eNOS, endothelial nitric oxide synthase; Egr-1, Early growth response gene-1; EF, ejection fraction; ER, endoplasmic reticulum; ERK, extracellular signal-regulated kinase; ET-1, endothelin-1; FAS, fatty acid synthase; FasL, fas ligand; FS, fractional shortening; GLP-1, glucagon-like peptide-1; GLUT1, glucose transporter 1; GM-CSF, granulocyte macrophage colony-stimulating factor; GPx, glutathione peroxidase; GSH, glutathione; hBSM, human bronchial smooth muscle cells; HEV, hepatitis e virus; HFD, high fat diet; HDL-C, high density lipoprotein cholesterol; HMEC-1, microvascular endothelial cells; HUVECs, human umbilical vein endothelial cells; ICAM, intercellular adhesion molecules; IFN-γ, interferon-γ; iNOS, inducible nitric oxide synthase; IL, interleukin; LVESP, left ventricular end-systolic pressure; InsR, insulin receptor; ISO, isoproterenol; JNK, c-Jun N-terminal kinase; KC, keratinocyte-derived chemokine; LVESD, left ventricular end systolic diameter; LVEDD, left ventricular end diastolic diameter; LVEDP, left ventricular end-diastolic pressure; LPS, lipopolysaccharide; mAChRs, muscarinic acetylcholine receptors; MAPK, mitogen-activated protein kinase; MCAO, middle cerebral artery occlusion; MCAO/R, middle cerebral artery occlusion/reperfusion; MCP-1, monocyte chemoattractant protein-1; MCT, monocrotaline; MDA, malondialdehyde; MHC, myosin heavy chain; MLE, mouse lung epithelial cells; MMP, matrix metalloproteinase; MPO, myeloperoxidase; mTOR, mammalian target of rapamycin; NF-κB, nuclear factor-κB; NOD, nucleotide-binding domain; NOD/SCID, nonobese diabetic/severe combined immunodeficiency; NSCLC, non-smallcell lung cancer; NLRP3, pyrin domain containing 3; OGD/R, oxygen–glucose deprivation/reoxygenation; OGTT, oral glucose tolerance test; ORF, open reading frame; PDGF-BB, platelet derived growth factor; PI3-Kp85, phosphoinositide 3-kinase p85 subunit; ROS, reactive oxygen species; PKC, protein kinase C; PMA, phorbol myristate acetate; PPAR, peroxisome proliferator-activated receptor; PSA, prostate-specific antigen; PTP1B, protein-tyrosine phosphatase 1B; S1P, sphingosine 1-phosphate; SCr, serum creatinine; SD, Sprague-Dawley; sICAM-1, human soluble intercellular adhesion molecule-1; SMG, submandibular gland; SOD, superoxide dismutase; SPHK1, sphingosine kinase-1; SREBP-1C, sterol regulatory element-binding protein 1c; STZ, streptozotocin; TEER, trans-endothelial electeical resistance; TC, total cholesterol; TF, tissue factor; TG, triglycerides; THP-1, human monoblastic leukemia cells; TJ, tight junction; TNF- α, tumour necrosis factor- α; TNFR, tumor necrosis factor receptor; TXNIP, thiredoxin-interactive protein; UA, uric acid; VCA,-1, vascular adhesion molecule-1; VEGF, vascular endothelial growth factor.

A critical assessment of pharmacological findings retrieved was conducted (Table 1). The application of excessively high dose, with over 1/3 publications, is seen as the most comm problem among the studies on liriopogons. Additionally, most of the high dose studies are on the pharmacological investigation of polysaccharides. The lack of controls using healthy cells in the evaluation of cytotoxic effects limits the scientific conclusion that can be drawn. Therefore, such studies were excluded. Similarly, eight out of eleven studies on ‘antioxidant’ effects included in the initial list of sources merely rely on chemical assays, which are of no therapeutic relevance and, therefore, were excluded. Methodological details are also assessed but not considered as exclusion criteria.

### Cardiovascular Protection

#### Ophiopogon japonicus

Protective effects on the cardiovascular system have been a core focus of research using both extracts and many metabolites isolated from *Ophiopogon japonicus*, including steroidal saponins and flavonoids. In essence, the level of cytokines such as interleukin (IL)-6, tumor necrosis factor (TNF)-α and IL-1β were reduced which inhibited the activation of NF-κB and MAPK pathway. Additionally, this protective effect is associated with antioxidant effect through improving antioxidant enzymes (superoxide dismutase (SOD), glutathione peroxidase (GSH-Px), catalase (CAT)).

Steroidal saponins from *Ophiopogon japonicus* mainly consisting of ophiopogonin B, ophiopogonin D (OPD) and ophiopogonin D’, significantly improved cardiac function of doxorubicin (DOX)-induced chronic heart failure (CHF) in rats linked to the increased blood pressure values of markers for left ventricular function, and the decreased value of left ventricular end-diastolic pressure (LVEDP), left ventricular end systolic diameter (LVESD) and left ventricular end diastolic diameter (LVEDD). This protective effect was achieved through suppressing oxidative stress and inflammatory response by improving SOD, GSH-Px, CAT and reducing inflammatory cytokine levels including IL-6, TNF-α and IL-1β (Wu et al., 2019). Methylophiopogonanone (MONB) exerted protective effects by increasing antioxidant potential in human umbilical vein endothelial cells (HUVECs), which is evidenced by the decreased production of malondialdehyde (MDA), ROS and increased SOD activity. Moreover, this effect might be associated to NADPH-related signaling by suppressing the expression of p22phox (an important component of NADPH oxidase) (Wang et al., 2019).

#### Liriope muscari

DT-13, a key biologically active steroidal saponin isolated from *L. muscari* has been investigated for the protective effects on the cardiovascular system through diverse ways. It protected C57BL/6 mice endothelium through inhibiting endothelium vascular inflammation by regulating nitric oxide (NO) production and the expression of ROS, tumor necrosis factor receptor (TNFR), IL-8, monocyte chemoattractant protein (MCP)-1 (Fan et al., 2018). The anti-thrombotic activity of DT-13 was observed in SD (Sprague-Dawley) rats by inhibiting thrombosis and down-regulating mRNA expression levels of IL-6 and tissue factor (TF) (Tian et al., 2013). Additionally, DT-13 showed anti-apoptosis activity on HUVECs by decreasing the expression of cleaved caspase-3 and cleaved poly (ADP ribose) polymerase (PARP) through regulating PI3 K/Akt signaling pathway (Qiu et al., 2014). Two homoisoflavonoids (metabolite 209 and 210) (Supplementary Table S2) exhibited anti-platelet activity at IC50 value of 11.59 and 10.69 μM (Tsai et al., 2013).

### Anti-Inflammatory Effects

Steroidal saponins, flavonoids and polysaccharide-rich fraction have been broadly studied for anti-inflammatory activities both *in vitro* and *in vivo*. In addition, a few phenols have also been assessed for anti-inflammatory activity. Lipopolysaccharides (LPS), monocrotaline (MCT), DNCB, IL-4 are applied for establishing the inflammatory models. In general, the bioactive metabolites inhibit the production of inflammatory cytokines, e.g., NO, IL-Iβ, IL-6, TNF-α etc., and suppress the phosphorylation of MAPK and NF-κB signaling pathways. Moreover, the anti-inflammatory effect may be achieved through reducing cell adhesion.

#### Ophiopogon japonicus

Sun et al. (2012) looked at the inhibitory effect of ruscogenin on LPS-induced mice with acute lung injury. Ruscogenin remarkably alleviated lung injury by attenauting LPS- induced myeloperoxidase (MPO) activity and nitrate/nitrite content, downregulating the expression of TF, iNOS, and regulating NF-κB pathway and NF-κB p-p65. The inhibitory effects of DT-13 on TNF-α-induced vascular inflammation and the potential molecular mechanisms were investigated as well. It diminished vascular inflammation through reducing adhesion molecules tandemed with regulating the Src/NF-kappa B/MAPK pathway by suppressing NF-кB p65 phosphorylation, TNF-α induced luciferase activities of ICAM-1 and VCAM (vascular adhesion molecule)-1, and p38 phosphorylation and Src degradation (Zhang et al., 2015b).

A range of homoisoflavonoids have been assessed for anti-inflammatory activity *in vitro*. 4′-O-Demethylophiopogonanone E, and MONA, ophiopogonone E, MONB and ophiopogonanone H were observed with signidicant anti-inflammatory activity. In LPS-induced RAW 264.7 cell and LPS-induced murine microglial cell BV-2, the production of NO was significantly suppressed, along with the decreased level of IL-1β and IL-6 (Li N. et al., 2012; Zhao et al., 2017).

#### Liriope muscari

The anti-inflammatory effect of DT-13 isolated from *L. muscari* was reported both *in vitro* and *in vivo*. Acute paw edema induced by histamine was reduced up to 17.2% by DT-13, *in vitro* assay indicated it also significantly suppressed the adhesion of HL-60 to ECV304 cells induced by TNF-α or 12-myristate 13-acetate (PMA) (Tian et al., 2011).

#### Liriope spicata

Two phenols (metabolite 279, 280) (Supplementary Table S4) from *L. spicata* were investigated for anti-inflammatory activities against neutrophil respiratory burst stimulated by PMA with IC50 value of 4.15 and 5.96 μM, respectively (Hu et al., 2011).

### Effects on the Endocrine System

A small number of studies have revealed the effects of metabolites isolated from liriopogons on the endocrine system, such as anti-obesity, anti-hyperlipidemic and anti-diabetic activities. Generally, KKay mice, rats (mice) fed with high fat diet (HFD) or induced by streptozotocin (STZ), and diabetic rats/mice models are normally applied to study the regulatory potential on the endocrine system.

#### Ophiopogon japonicus

Ruscogenin and OPD isolated from *O. japonicus* act on the endocrine system. OPD exerted anti-obesity effect on HFD-induced metabolic syndrome mice through regulating gut microbiota, precisely, by reducing *Firmicutes/Bacteroidetes* ratios and endotoxin-bearing *Proteobacteria* levels (Chen S. et al., 2018). In addition, OPD played a protective role against renal damage in STZ-induced diabetic nephropathy through suppressing oxidative damage as evidence by the decreased level of MDA and increased activity of SOD, GSH, CAT, along with inflammatory response by reducing pro-inflammatory cytokines (IL-6, IL-1β) (Qiao et al., 2020). Ruscogenin was also reported can improve diabetic nephropathy (Lu et al., 2014).

Pretreatment with MO-A (10 mg/kg), a homoisoflavonoid, significantly ameliorated the hyperlipidemia in rats induced by high fat diet HFD through regulating the serum lipid profile by modulating the expression levels involved in lipogenesis and lipid oxidation, i.e., inducing the expression levels of both low-density lipoprotein receptor (LDLR) and peroxisome proliferators-activated receptors (PPAR) α, and suppressing the expression levels of both acetyl CoA carboxylase (ACC) and sterol regulatory element-binding protein 1c (SREBP-1C) (Li et al., 2020).

#### Liriope spicata

Two polysaccharides (LSP1, LSP2) obtained from *L. spicata* have been investigated for anti-diabetic activity. Chen et al. (2009a) evaluated their effects on STZ-induced diabetic mice and observed a remarkable reduction of fasting blood glucose, improvement of glucose tolerance and insulin resistance, as well as the decreased level of cholesterol. Moreover, they also exerted anti-diabetic effects on insulin-resistant diabetic KKAy mice through up-regulating the expression of insulin-receptor (InsR), insulin-receptor substrate-1 (IRS-1), phosphatidylinositol 3-kinase, and PPAR *γ* (Liu et al., 2013). The anti-diabetic activity of aqueous-ethanol extract of *L. spicata* was observed through inhibiting the expression of ICAM-1, MCP-1, and fibronectin protein and inflammatory cytokines (Lu et al., 2013).

### Immunomodulation

Th1/Th2 cytokine imbalance may play a role in the pathogenesis of Sjogren’s syndrome SS (Price and Venables, 1995). *O. japonicus* polysaccharides (OJP) was found to significantly improve the SS in mouse via the regulation of Th1/Th2 cytokine imbalance by reducing IFN-γ level and IFN-γ/IL-4 ratio (Wang et al., 2007).

### Antioxidative Effects

Up to now, OPD and FOB-8, the bioactive metabolites from *O. japonicus* have been reported for significant antioxidative activities both *in vitro* and *in vivo*.

OPD played a protective role as an effective antioxidant agent in H_2_O_2_-induced endothelial injury by decreasing H_2_O_2_-induced oxidative stress through inhibiting the activation of ERK1/2 (Qian et al., 2010). Moreover, it demonstrated anti-osteoporosis activity both *in vitro* and *in vivo*, through decreasing oxidative stress which was related to FoxO3a-β-catenin signaling pathway by down-regulating the protein expression of β-catenin, mRNA expressions of Axin2 and OPG (Huang et al., 2015).

8-FOB showed protective effect against paraquat-induced hepatotoxicity through suppressing oxidative stress by attenuating MDA levels and GSH and SOD levels (Qian et al., 2019).

### Cytotoxic and Anti-Cancer Activity

The anti-cytotoxic and cancer activities of steroidal saponins isolated from liriopogons have been investigated, including the effects on prostate cancer, lung cancer, colorectal cancer, liver cancer, and the possible mechanisms were also studied. Generally, the anti-cancer effects of liriopogons are achieved by inducing apoptosis, suppressing glucose transporter 1 (GLUT1) transmembrane glucose pathway, and inhibiting proliferation and angiogenesis.

#### Ophiopogon Japonicus

Ophiopogonin D′, an active metabolite from *O. japonicus*, suppressed the growth of PC3 and DU145 xenograft tumors (prostate cancer) in BALB/c nude mice through inducing apoptosis. The cellular mechanism of this activity might be through modulating RIPK1-related pathway as evidenced by the increased protein expression of RIPK1 and Bcl-2-like protein 11, and the decreased levels of cleaved-RIPK1, caspase 8, cleaved-caspase 8, Bid, caspase 10, and cleaved-caspase 10 (Lu et al., 2018). DT-13 inhibited the proliferation of colorectal cancer in orthotopic implantation mouse model of colorectal cancer model and C57BL/6J APC^min^ mice model were reported, which is associated with GLUT1 transmembrane glucose pathway. The results indicated that DT-13 significantly suppressed GLUT1 and activating AMPK/mTOR pathway (Wei et al., 2019). Sprengerinin C inhibited the angiogenesis in HUVECs through repressing the activation of VEGFR2-dependent PI3K/Akt/mTOR and p38 MAPK signaling pathways by down-regulating the expression of MMP-2/9 and VEGF. Meanwhile, a significant improvement of reactive oxygen species, cleaved caspase-3 and cleaved PARP was detected after DT-13 treatment on HepG-2 and BEL7402 cells, which induced the apoptosis (Zeng et al., 2013).

#### Liriope muscari

Ophiopogon Saponin C1, the bioactive metabolite of *L. muscari* against lung tumor through stablizing endothelium permeability by inhibiting the disassembly of ZO-1 protein, TNF-α and repressing PKCδ and Src kinase (Zhang et al., 2020).

### Anti-Viral Activity

Only two steroidal saponins isolated from *L. muscari* have been investigated for anti-viral activity *in vitro*.

The effect of metabolite 207 (Supplementary Table S2) against hepatitis B virus was reported (Huang et al., 2014) as shown by the decreased level of viral gene expression and viral DNA replication. This was possibly regulated through NF-κB signaling pathway by decreasing the expression of p65/p50 NF- κB protein and phosphorylated NF-κB p65, simultaneously elevating cytoplasmic IκBα protein levels.

Park et al. (2019) looked at the anti-viral activity of the steroidal saponin spicatoside A on hepatitis E virus (HEV). It inhibited the replication of HEV genotype 3 strain 47832c replicon in a concentration-dependent manner, and down-regulating the expression of HEV open reading frame 2 (ORF2).

### Others

Other pharmacological benefits such as anti-tussive and neuroprotective effects and the therapeutic effects on acute myeloid leukemia (AML) have also been evaluated, but received far less attention.

Ophiopogonin D isolated from *O. japonicus* exerted anti-tussive activity by hyperpolarizing the paratracheal neurones from a resting membrane potential of -65.7 to -73.5 mV (Ishibashi et al., 2001). Ethanol extract of *L. muscari* was reported for neuroprotective effect by attenuating intracellular oxidative stress and mitochondrial dysfunction, where PARP and caspase-3 cleavage was suppressed (Park et al., 2015).

The anti-AML activity of DT-13 was investigated *in vitro* and *in vivo*. It induced the apoptosis AME cells, especially HL-60 and Kasumi-1 cells through modulating death receptor pathway by enhancing the expression of cleaved-PARP and cleaved-caspase 3 and 8. Moreover, the differentiation of AML cells was promoted by DT-13 as shown by the increased level of differentiation markers CD11b and CD14, as well as transcription factor C/EBPα and C/EBPβ. *In vivo* evaluation was carried out on NOD/SCID mice with the engraftment of HL-60 cells revealing the anti-leukemia activity of DT-13 (Wang C. et al., 2020).

The hepatoprotective effect has also been reported. 58-F, a flavanone isolated from *O. japonicus*, protected against hepatocyte from death through lowering lysosomal membrane permeability as shown by the increased the fluorescence intensity of the LysoTracker Green and cell viability, and through elevating lysosomal enzyme translocation to the cytosol as evidenced by the suppressed activity of cathepsin B and cathepsin D (Yan et al., 2016).

## Toxicological Assessments of Liriopogons

Liriopogons are noted for their therapeutic benefits with little recorded toxicity since *Shenong’s Canon on Materia Medica* (ca. 200–250 CE), but also in numerous contemporary medical monographs (Huang, 1982; Li et al., 2011). Only a few scientific studies have been conducted on toxicological properties of liriopogons but confirmed the traditional cognition.

*O. japonicus* decoction showed no chromosome damage of bone marrow cells in ICR mice, and no genotoxicity *in vivo* with metabolic activation (Hu et al., 2009). Moreover, a *O. japonicus* decoction was investigated for the potential development of toxicity in rats by evaluating the maternal body weight, fetus weight and viability, incidences of fetalmal formation and variation, showing no obvious adverse effect (Min et al., 2010).

## Conclusion and Perspectives

The metabolites and pharmacological activities of liriopogons are reasonably well understood and this supports the idea of the two genera *Ophiopogon* and *Liriope* forming – in ethnopharmacological terms – a plant complex. Some species are also relatively well known pharmacologically. Steroidal saponins, flavonoids and polysaccharides are the major classes of metabolites in both genera. Several organic acids, phenols and other types of metabolites have also been isolated. Crude extracts and isolated pure metabolites from liriopogons exhibit a wide spectrum of reported pharmacological properties. Especially, steroidal saponins and flavonoids have been linked to experimental pharmacological studies focusing on cardiovascular diseases and inflammatory syndromes. However, clinical evidence needs to be developed. Despite the extensive studies on liriopogons, the focus has mainly been on three species - *O. japonicus*, *L. muscari* and *L. spicata*. Less emphasis has been placed on other species, which are also traditionally used as local and traditional medicines, such as *L. gramilifolia*, *O. dracaenoides*, *O. platyphyllus* and *O. reversus* (Li et al., 2006; Zheng and Xing, 2009), leaving a large open area for future investigations. On the other hand, studies generally have only focused on broad ranging *in vivo* effects and not on molecular mechanisms, which need to be explored further, e.g., the modulation of pathways, along with related cytokines and genes.

Limited evidence exists with regards to the species’ safety and specifically, there is a lack of assessing potential toxicological effects of liriopogons. In general, the findings indicated that, consistent with traditional perception, decoctions derived from the species have a low toxicity. However, this is clearly insufficient from a clinical perspective.

We also critically assessed the experimental approaches (Table 1) and identified a number of problems, which make an assessment of the species’ potential benefits difficult if not impossible. In experimental terms, the use of excessively high dose levels needs to be addressed. Numerous studies reviewed use high dose levels resulting in these results being of very limited scientific relevance. Especially in case of *in vivo* studies, the dose per day and kg body weight often seems to be of limited or no therapeutic relevance. The use of such high doses is often justified with the rate of metabolism being higher in rodent models. While the calculations commonly used in drug discovery (where the starting values in humans are nano or microMol) makes sense, this is not meaningful if the starting dose is higher like in traditional (tea) preparations. Moreover, the majority of high dose studies are on pharmacological investigations of polysaccharides. Evaluations of potential cytotoxic effects on liriopogons lack controls using healthy cells, making it impossible to assess the specificity of the effect. Investigations of potential chemical antioxidant effects cannot make pharmacological claims based on such assays.

In the current review, we present the case study of the liriopogons in order to assess how the pharmacological evaluation of extracts needs to be improved in experimental terms. Methodological details provided are also evaluated (Table 1). In general, the extraction process of the crude extracts or pure metabolites and characterization are well described. However, the link between local/traditional uses and the pharmacological assessment is often vague or is not reflected in the publications.

All this is not just specific to studies on liriopogons, but represents a more general situation of the current state of ethnopharmacological research. When conducting pharmacological assays, researchers need to reassess what constitute therapeutically meaningful doses, in particular for the pharmacological assessment of polysaccharides. Moreover, the assessment of anti-oxidant effects need to of pharmacological relevance. Appropriate controls in cytotoxic studies, further investigation on toxicological properties, and molecular mechanism and clinical evidence of diverse pharmacological activities are also required.

Therefore, it is essential, and our responsibility, to use rigorous scientific approaches and to deliver high quality findings for the future benefits of patients, and for the better development of evidence-based natural products.
