# Supplementary material for: Liriopogons (Genera Ophiopogon and Liriope, Asparagaceae): A Critical Review of the Phytochemical and Pharmacological Research
Source: Front Pharmacol. 2021 Dec 3;12:769929. doi: 10.3389/fphar.2021.769929 (PMC8678496; doi:10.3389/fphar.2021.769929)
Supplement: Supplementary file 1 [file DataSheet1.pdf]

Supplementary Material

Supplementary Table 1 Steroidal saponins isolated from liriopogons

No.	Metabolites	Plant source	Plant part investigated (as stated in the original source)[a]	Type	Structures								References
					R_1	R_2	R_3	R_4	R_5	R_6	R_7	25-C	
1	glycoside A	*L. muscari*	Subterranean part (underground part)	A	OH	rha	H	H	H	H	H	R	(Watanabe et al., 1983a)
2	glycoside B	*L. spicata*	Subterranean part (subterranean part)	A	O-fuc	rha	H	H	H	H	H	S	(Yu et al., 1990)
		L. muscari	Subterranean part (underground part)										(Watanabe et al., 1983a)
		L. graminifolia	Whole plant (whole plant)										(Wang, 2010)
3	glycoside C	*O. japonicus*	Subterranean part (underground part)	A	rha(1→2)fuc	H	H	H	H	H	H	S	(Asanno et al., 1993b)

		L. muscari	Subterranean part (underground part)										(Nakakuki, 1986; Watanabe et al., 1983a)
4	glycoside D	*L. muscari*	Subterranean part (underground part)	A	H	glc (1→3)rha	H	H	H	H	H	R	(Watanabe et al., 1983a)
5	glycoside G	*L. muscari*	Subterranean part (underground part)	A	O-SO_3M^b	rha	H	H	H	H	H	R	(Watanabe et al., 1983a)
6	(25S)-ruscogenin 1-O-sulfate-3-O-α-L-rhamnopyranoside	*L. graminifolia*	Subterranean part (underground part)	A	O-SO_3M^b	rha	H	H	H	H	H	S	(Wang et al., 2012a)
7	(25R, S)-ruscogenin 1-O-sulfate-3-O-α-L-rhamnopyranoside	*L. graminifolia*	whole plant (whole plant)	A	O-SO_3M^b	rha	H	H	H	H	H	R,S	(Wang, 2010)
8	glycoside H	*L. muscari*	Subterranean part (subterranean part)	Supplementray Figure 2									(Watanabe et al., 1983a)
9	glycoside J	*L. spicata*	Fibrous root (fibrous root)	A	O-rha(1→2)xyl(1→3)fuc	H	H	H	H	H	H	S	(Qi et al., 2015)
10	LS-10	*O. japonicus*	Subterranean part (underground part)	A	O-rha(1→2)xyl(1→3)fuc	H	H	H	H	H	H	S	(Asanno et al., 1993b)

11	nolinospiroside F	*O. japonicus*	Unclear	A	O-fuc	rha	H	H	H	H	H	S	(Sun et al., 2013)
12	25(S)-ruscogenin	*O. japonicus*	Tuberous root (tuber)	A	OH	H	H	H	H	H	H	S	(Liu et al., 2006)
13	25(R)-ruscogenin	*O. japonicus*	Tuberous root (tuber)	A	OH	H	H	H	H	H	H	R	(Liu et al., 2006)
14	25(R, S) ruscogenin	*L. spicata*	Fibrous root (fibrous root)	A	H	H	H	H	H	H	H	R,S	(Qi et al., 2015)
15	25(R, S) ruscogenin 1-O-β-D-fucopyranoside	*L. spicata*	Fibrous root (fibrous root)	A	O-fuc	H	H	H	H	H	H	R,S	(Qi et al., 2015)
16	25(R) ruscogenin 1-O-β-D-fucopyranoside	*L. muscari*	Tuberous root (tuber)	A	O-fuc	H	H	H	H	H	H	R	(Li et al., 2014a)
17	25(S)-ruscogenin-1-O-β-D-glucopyranosyl-(1→2)-[α-L-arabinofuranosyl-(1→3)]-β-D-fucopyranoside	*L. muscari*	Subterranean part (subterranean part)	A	O-glc(1→2)ara(1→3)fuc	H	H	H	H	H	H	S	(Wu et al., 2017)
18	(25R)-ruscogenin-1-O-β-D-glucopyranosyl-(1→2)-[α-L-arabinofuranosyl-(1→3)]-β-D-fucopyranoside	*L. muscari*	Subterranean part (subterranean part)	A	O-glc(1→2)ara(1→3)]fuc	H	H	H	H	H	H	R	(Wu et al., 2017)
19	25(S) ruscogenin 1-O-β-D-fucopyranoside-3-O-α-L-rhamnopyranoside	*L. spicata*	Subterranean part (subterranean part)	A	O-fuc	rha	α-L-rha	H	H	H	H	S	(Yu et al., 1990)
		L. muscari	Subterranean part (subterranean part)										(Watanabe et al., 1983a)

20	ruscogenin1-O-sulfate	*O. japonicus*	Subterranean part (underground part)	A	O-SO_3M	H	H	H	H	H	H	R	(Asanno et al., 1993b)
21	(25R)-ruscogenin-3-yl α- L-rhamnopyranosyl-(1→2)-[β-D-xylopyranosyl-(1→4)]-β-D-glucopyranoside	*O. japonicus*	Fibrous root (fibrous root)	A	H	O-rha(1→2)xyl-(1→4)glc	H	H	H	H	H	R	(Duan et al., 2010a)
22	(25R)-3β-hydroxyspirost-5-en-1-β-yl-3-O-α-L-rhamnopyranosyl-(1→2)-O-β-D-xylopyranosyl-(1→3)-α-L-arabinopyranoside	*O. japonicus*	Subterranean part (root)	A	H	rha-O-xyl (1→3)ara	H	H	H	H	H	R	(Wang et al., 2011b)
23	(23S,24S,25S)-23,24-dihydroxyruscogenin1-O-[α-L-rhamnopyranosyl(1→2)][β-D-xylopyranosyl(1→3)]-α-L-arabinopyranoside24-O-β-D-fucopyranoside	*O. japonicus*	Subterranean part (underground part)	A	O-rha(1→2)xyl(1→3)]ara	H	H	H	H	OH	fuc	S	(Asanno et al., 1993b)
24	(23S,24S,25S)-23,24-dihydroxyruscogenin1-O-[α-L-2,3,4-tri-O-acetylrhamnopyranosyl (1→2)][β-D-xylopyr- anosyl (1→3)]-α-L-arabinopyranoside24-O-β-D-fucopyranoside	*O. japonicus*	Subterranean part (underground part)	A	O-2,3,4-tri-O-Ac rha (1→2)xyl(1→3)ara	H	H	H	H	OH	fuc	S	(Asanno et al., 1993b)
25	diosgenin	*O. japonicus*	whole plant (whole plant)	A	H	H	H	H	H	H	H	Unknown	(Okanishi et al., 1975)
26	diosgenin-3-O-[2-O-acetyl-α-L-rhamnopyranosyl-(1→2)][β-D-xylopyranosyl-	*O. japonicus*	Fibrous root (fibrous root)	A	H	2-O-Ac-rha-(1→2)xyl-(1→4)glc	H	H	H	H	H	Unknown	(Duan et al., 2010a)

	(1→4)]-β-D-glucopyranoside											
27	ophiogenin3-O-α-L-rhamnopyranosyl-(1→2)-β-D-glucopyranoside	*O. japonicus*	Subterranean part (root)	A	H	rha(1→2)glc	H	OH	OH	H	H	R	(Adinolfi et al., 1990)
28	disogenin 3-O-α-L-rhamnopyranosyl-(1→2)-β-D-glucopyranosyl-(1→4)-β-D-glucopyranosid	*O. planiscapus*	Subterranean part (subterranean part)	A	H	rha(1→2)glc(1→4) glc	H	H	H	H	H	unknown	(Watanabe et al., 1983b)
29	bornyl7-O-α-L-arabinofuranosyl(1-6)-β-D-glucopyranoside	*O. japonicus*	Tuberous root (tuberous root)	A	H	ara(1→6)glc	H	OH	OH	H	H	R	(Adinolfi et al., 1990)
30	cixi-ophiopogon A	*O. japonicus*	Tuberous root (tuber)	A	H	rha(1→2)xyl(1→3)glc(1→4)glc	H	OH	OH	H	H	R	(Chen et al., 2000)
31	cixi-ophiopogon B	*O. japonicus*	Tuberous root (tuber)	A	H	rha(1→2)xyl(1→3)glc(1→4)glc	H	OH	OH	H	H	R	(Chen et al., 2000)
32	(25R)-14α,17α-hydroxyspirost-5-en-3β-yl3-O-α-L-rhamnpyranosyl-(1→2)-β-D-glucopyranosyl-(1→3)-β-D-glucopyranoside	*O. japonicus*	Tuberous root (tuberous root)	A	H	rha(1→2) glc(1→3)glc	H	OH	OH	H	H	R	(Wang et al., 2011b)
33	pennogenin-3-O-α-L-rhamnopyranosyl-(1→2)-β-D-xylopyranosyl-(1→3)-β-D-glucopyranosid	*L. muscari*	Tuberous root (tuberous root)	A	H	rha-(1→2)xyl(1→3)glc	H	H	OH	H	H	R	(Li et al., 2014a)
34	floribundasaponin B	*O. japonicus*	Tuberous root (tuber)	A	H	rha(1→2)xyl(1→4)	H	H	OH	H	H	R	(Wang et al., 2008)

35	pennogenin-3-O-[2-O-acetyl-α-L-rhamnopyranosyl-(1→2)][β-D-xylopyranosyl-(1→4)]-β-D-glucopyranoside	*O. japonicus*	Tuberous root (tuber)	A	H	Ac-rha(1→2)xyl(1→4)glc	H	H	OH	H	H	R	(Wang et al., 2008)
36	pennogenin-3-O-[4'-O-acetyl-α-L-rhamnopyranosyl-(1→3)][β-D-xylopyranosyl-(1→4)]-β-D-glucopyranoside	*L. muscari*	Tuberous root (tuber)	A	H	4'-O-Ac-rha-(1→3)xyl(1→4)glc	H	H	OH	H	H	R	(Cheng et al., 2006a)
37	3-O-β-D-xylopyranosyl-(1→4)-β-D-glucopyranoside	*O. japonicus*	Tuberous root (tuber)	A	H	xyl(1→4)glc	H	H	OH	H	H	R	(Cheng et al., 2006b)
38	(25R) spirost-5-ene-3β,14α-diol-3-β-O-β-L-rhamnopyranosyl(1α-dioβ-D-xylopyranosyl(1α-dβ-D-glucopyranoside	*O. japonicus*	Tuberous root (tuber)	A	β-D-glu	rha-dio-xylopyranosyl	H	H	H	H	H	R	(Xu et al., 2008a)
39	(25S) spirost-5-ene-3β,17α-diol-3-O-[β-D-xylopyranosyl(1→3)][α-L-arabinofuranosyl(1→2)][α-L-rhamnopyranosyl(1→4)]-β-D-glucopyranoside	*L. graminifolia*	whole plant	A	H	xyl(1→3)ara(1→2)rha(1→4)glc	H	H	H	H	H	S	(Wang, 2010)
40	(25R) ruscogenin 1-O-α-L-rhamnopyranosyl-(1→2)-β-D-xylopyranoside	*L. graminifolia*	Subterranean part (underground part)	A	rha-(1→2)xyl	H	H	H	H	H	H	R	(Wang et al., 2011a)
41	(25S) ruscogenin 1-O-α-L-rhamnopyranosyl-(1→2)-β-D-xylopyranoside	*L. spicata*	Tuberous root (tuber)	A	O-rha(1→2)xyl	H	H	H	H	H	H	S	(Qi et al., 2015; Yu et al., 1990)

42	14-hydroxydiosgenin3-O-α-L-rhamnopyranosyl-(1→2)-β-D- glucopyranoside	*O. japonicus*	Tuberous root (tuber)	A	H	rha(1→2)glc	H	H	H	H	H	R	(Li et al., 2013)
43	14-hydroxydiosgenin3-O-α-L-rhamnopyranosyl-(1→2)-β-D-xylopyranosyl-(1→4)-β-D-glucopyranoside	*O. japonicus*	Whole plant (whole plant)	A	H	rha(1→2)xyl(1→4) glc	H	H	H	H	H	R	(Li et al., 2013)
44	prazerigenin A	*O. japonicus*	Subterranean part (underground part)	A	H	H	H	OH	H	H	H	R	(Zhou et al., 2013a)
45	sprengerinin A	*O. japonicus*	Tuberous root (tuber)	A	H	xyl(1→4)glc	H	H	H	H	H	R	(Li et al., 2013)
46	sprengerinin C	*O. japonicus*	Tuberous root (tuber)	A	H	rha(1→2)xyl(1→4)	H	H	H	H	H	R	(Li et al., 2013)
47	25(R, S) ruscogenin 1-O-[3-O-acetyl-α-L-rhamnopyranosyl-(1→2)]-β-D-fucopyranoside	*L. spicata*	Subterranean part (underground part)	A	O-3-O-Ac-rha(1→2)fuc	H	H	H	H	H	H	R,S	(Yu et al., 1990)
48	(25R, S) ruscogenin 1-O-α-L-rhamnopyranosyl(1→2)] [β-D-xylopyranosyl (1→3)]-β-D-frucopyranosyl-3-O-α-L- rhamnopyranoside	*L. muscari*	Tuberous root (tuberous root)	A	O-rha(1→2)xyl(1→3)fuc	rha	H	H	H	H	H	R,S	(Li et al., 2014a)
49	lirioprolioside A	*L. spicata*	Subterranean part (subterranean part)	A	O-rha(1→2)xyl(1→3)]fuc	rha	H	H	H	H	H	S	(Yu et al., 1990)
50	lirioprolioside B	*L. spicata*	Subterranean part	A	O-Acrha(1→2)fuc	H	H	H	H	H	H	S	(Yu et al., 1996)

			(subterranean part)										
51	lirioprolioside C	*L. spicata*	Subterranean part (subterranean part)	A	acetyl-rha(1→2)fuc	H	H	H	H	H	H	S	(Yu et al., 1996)
52	lirioprolioside D	*L. spicata*	Subterranean part (subterranean part)	A	H	O-(3-O-Ac)rha(1→2)fuc	H	H	H	H	H	R	(Yu et al., 1996)
53	ruscogenin1-O-α-L-rhamnopyranosyl-(1→2)-4-O-sulfo-α-L-arabinopyranoside-3-O-β-D-glucopyranoside	*O. japonicus*	Tuberous root (tuber)	A	O-rha(1→2)-4-O-sulfo-ara	glc (1→3)rha	H	H	H	H	H	R	(Dai et al., 2005; Lan et al., 2013)
		L. spicata	Fibrous root (fibrous root)										(Qi et al., 2015)
54	ruscogenin1-O-α-L-rhamnopyranosyl-(1→2)-4-O-sulfo-α-L-arabinopyranoside	*O. planiscapus*	Subterranean part (subterranean part)	A	O-rha(1→2)-4-O-sulfo-ara	H	H	H	H	H	H	unknown	(Watanabe et al., 1983b)
55	neoruscogenin1-O-α-L-rhamnopyranosyl-(1→2)-4-O-sulfo-α-L-arabinopyranoside-3-O-β-D-glucopyranoside	*O. jaburan*	Tuberous root (tuber)	D	O-rha(1→2)-4-O-sulfo-ara	glc	-	-	-	-	-	-	(Watanabe et al., 1984)
56	(25S) ruscogenin 1-O-((2-O-acetyl)-α-L-rhamnopyranosyl(1→2))(β-D-xylopyranosyl(1→3))-β-D-fucopyranoside	*L. spicata*	Subterranean part (subterranean part)	A	H	O-(2-O-Ac)rha(1→2)xyl(1→3)fuc	H	H	H	H	H	S	(Yu et al., 1990)

57	(25S) ruscogenin 1-O-[(3-O-acetyl)-α-L-rhamnopyranosyl(1→2)][(β-D-xylopyranosyl(1→3)]-β-D-fucopyranoside	*O. japonicus*	Tuberous root (tuber)	A	O-(3-O-acetyl)rhal(1→2) xyl(1→3)uc	H	H	H	H	H	H	S	(Dai et al., 2005)
		L. spicata	Subterranean part (subterranean part), fibrous root (fibrous root)										(Qi et al., 2015; Yu et al., 1990)
58	DT-13	*L. muscari*	Tuberous root (tuber)	A	O-glc(1→2)xyl(1→3)fuc	H	H	H	H	H	H	S	(Ma et al., 2011)
		O. japonicus	Tuberous root (tuberous root)										(Adinolfi et al., 1990)
59	26-O-β-D-glucopyranosyl(25S)-furost-5(6)-ene-1β,3β-26- triol-1-O-[β-D-glucopyranosy-(1→2)][β-D-xylopyranosyl-(1→3)]-β-D-fucopyranoside	*L. muscari*	Tuberous root (tuber)	B	O-glc(1→2)xyl(1→3)]fuc	H	H	H	H	CH3	glc	O-glc(1→2) xyl(1→3)fuc	(Lee et al., 1989)
60	25(S)-ruscogenin 1-O-[β-D-fucopyranosyl(1→2)] [β-D-xylopyranosyl (1→4)]-β-D-frucopyranoside	*L. muscari*	Tuberous root (tuberous root)	A	O-fuc(1→2)xyl (1→4)-fuc	H	H	H	H	H	H	S	(Li et al., 2014a)
		L. spicata	Tuberous root (tuber)										(Do et al., 1995)
61	26-O-β-D-glucopyranosyl(25S)-furost-5(6)-ene-1β,3β,22α-26-tetraol-1-O-[β-D-glucopyranosy-(1→2)][β-D-	*L. muscari*	Tuberous root (tuber)	B	O-glc(1→2)xyl(1→3)]fuc	H	H	H	H	CH3	glc	O-glc(1→2) xyl(1→3)fuc	(Choi et al., 2015)

	xylopyranosyl-(1→3)]-β-D-fucopyranoside												
62	25(R, S)-ruscogenin 1-O-[β-D-fucopyranosyl(1→2)] [β-D-xylopyranosyl (1→4)]-β-D-frucopyranoside	*L. muscari*	Tuberous root (tuberous root)	A	O-fuc(1→2)xyl(1→4)fuc	H	H	H	H	H	H	R,S	(Li et al., 2015)
63	25(R)-ruscogenin 1-O-[β-D-fucopyranosyl(1→2)] [β-D-xylopyranosyl (1→3)]-β-D-glucopyranoside	*L. muscari*	Tuberous root (tuberous root)	A	O-fuc(1→2)xyl(1→3)fuc	H	H	H	H	H	H	R	(Li et al., 2014a)
64	25(S)-ruscogenin 1-O-[β-D-fucopyranosyl(1→2)] [β-D-xylopyranosyl (1→3)]-β-D-glucopyranoside	*L. muscari*	Tuberous root (tuberous root)	A	O-fuc(1→2)xyl(1→3)fuc	H	H	H	H	H	H	S	(Li et al., 2014a)
65	(25R)-ruscogenin-1-O-β-D-glucopyranosyl-(1→2)-[β-D-xylopyranosyl (1→3)]-β-D-glucopyranoside	*L. muscari*	Subterranean part (subterranean part)	A	O-glc(1→2)xyl (1→3)glu	H	H	H	H	H	H	R	(Wu et al., 2017)
66	25(S)-ruscogenin 1-O-[β-D-glucopyranosyl (1→2)] [β-D-xylopyranosyl (1→3)]-β-D-glucopyranoside	*L. muscari*	Subterranean part (subterranean part)	A	O-glu(1→2)xyl (1→3)glu	H	H	H	H	H	H	S	(Wu et al., 2017)
67	ophiogenin	*O. japonicus*	Tuberous root (tuber)	A	H	H	H	OH	OH	H	H	R	(Tada et al., 1980b)
68	ophiopojaponin A	*O. japonicus*	Tuberous root (tuber)	A	’	2'-O-Ac-rha(1→2)xyl (1→3)glc	H	H	OH	H	H	R	(Dai et al., 2000)
69	ophiopojaponin B	*O. japonicus*	Tuberous root (tuber)	B	H	rha(1→2)glc	H	H	OH	H	glc	H	(Dai et al., 2000)

70	ophiopojaponin C	*O. japonicus*	Tuberous root (tuber)	A	H	rha(1→2)xyl(1→4) glc	H	OH	OH	H	H	H	(Dai et al., 2005)
71	ophiopogonin A	*O. japonicus*	Tuberous root (tuberous root)	A	H	O-[(3-O-Ac)-rha(1→2)fuc	H	H	H	H	H	H	(Watanabe et al., 1977)
		L. spicata	Subterranean part (subterranean part)										(Yu et al., 1996)
72	ophiopogonin B	*O. japonicus*	Tuberous root (tuber)	A	O-rha(1→2)fuc	H	H	H	H	H	H	O-rha(1→2)fuc	(Tada & Shoji, 1972)
		L. spicata	Fibrous root (fibrous root)										(Qi et al., 2015)
73	ophiopogoninB’	*O. japonicus*	Tuberous root (tuberous root)	A	H	[(4-O-Ac)-rha (1→2)xyl(1→3)glc	H	H	H	H	H	H	(Watanabe et al., 1977)
74	ophiopogonin C	*O. japonicus*	Tuberous root (tuberous root)	A	O-rha(1-2)xyl(1-3)uc	Ac	H	H	H	H	H	O-rha(1-2)xyl(1-3)uc	(Watanabe et al., 1977)
75	ophiopogoninC’	*O. japonicus*	Tuberous root (tuberous root)	A	H	rha (1→2)glc	H	H	H	H	H	R	(Watanabe et al., 1977)
76	ophiopogonin D	*O. japonicus*	Tuberous root (tuberous root)	A	O-rha(1-2)xyl(1-3)fuc	H	H	H	H	H	H	R	(Tada et al., 1973)

		L. muscari	Tuberous root (tuber)										(Park et al., 2014)
		L. spicata	Subterranean part (subterranean part)										(Yu et al., 1996)
		O. jaburan	Tuberous root (tuber)										(Watanabe et al., 1984)
77	ophiopogonin D’	*O. japonicus*	Tuberous root (tuber)	A	H	rha (1→2)xyl (1→3)glc	H	H	H	H	H	unknown	(Watanabe et al., 1977)
78	Ophiopogonin E	*O. japonicus*	Tuberous root (tuber)	A	H	xyl(1→4)glc	H	H	OH	H	H	R	(Cheng et al., 2006b)
79	ophiopogonin F	*O. japonicus*	Tuberous root (tuber)	B	H	xyl(1→4)-O-rha(1→2)glc	H	H	H	H	glc(1→ 2)gl c	H	(Zhang et al., 2009)
80	ophiopogonin G	*O. japonicus*	Tuberous root (tuber)	B	H	xyl(1→4)-O-rha(1→2)glc	H	H	H	H	glc(1→ 6)gl c	H	(Zhang et al., 2009)
81	ophiopogonin H	*O. japonicus*	Fibrous root (fibrous root)	B	H	rha(1→ 2)]glc	H	H	H	H	glc(1→ 2)gl c	H	(Duan et al., 2010b)
82	ophiopogonin J	*O. japonicus*	Fibrous root (fibrous root)	C	rha(1→2)xyl(1→ 4)]glc	glc(1→2)glc	-	-	-	-	-	-	(Duan et al., 2012)

83	ophiopogonin I	*O. japonicus*	Fibrous root (fibrous root)	C	rha-(1→ 2)glc	glc-(1→2)glc	-	-	-	-	-	-	(Duan et al., 2010b)
84	ophiopogonin K	*O. japonicus*	Tuberous root (tuber)	B	H	rha(1→2)glc	H	OH	OH	H	glc(1→2)glc	H	(Zhang et al., 2012a)
85	ophiopogonin L	*O. japonicus*	Tuberous root (tuber)	B	H	rha(1→2)xyl(1→4)glc	OH	OH	H	H	glc	H	(Zhang et al., 2012a)
86	ophiopogonin M	*O. japonicus*	Tuberous root (tuber)	B	H	xyl(1→4)rha(1→2)glc	OH	OH	OH	H	glc	H	(Zhang et al., 2012a)
87	ophiopogonin N	*O. japonicus*	Tuberous root (tuber)	B	H	rha(1→2)xy-(1→4)glc	H	OH	H	H	glc(1→6)glc	H	(Zhang et al., 2012a)
88	ophiopogonin S	*O. japonicus*	Tuberous root (tuberous root)	A	H	xyl-(1→4)glc	H	OH	H	H	H	R	(Li et al., 2013)
89	ophiopogonin O	*O. japonicus*	Tuberous root (tuber)	A	H	rha(1→2)xyl-(1→4)glc	H	OH	OH	H	glc	R	(Zhang et al., 2012a)
90	ophiopogonin P	*O. japonicus*	Tuberous root (tuberous root)	A	H	4-O-Ac-rha(1→2)(1→4)glc	H	H	H	H	H	Unknown	(Li et al., 2013)
91	ophiopogonin Q	*O. japonicus*	Tuberous root (tuberous root)	A	H	rha (1→2)[2-O-Ac-xyl(1→4)]glc	H	H	H	H	H	Unknown	(Li et al., 2013)

92	ophiopogonin R	*O. japonicus*	Tuberous root (tuberous root)	A	H	rha-(1→2)glc	OH	OH	OH	H	H	R	(Li et al., 2013)
93	ophiopogonin T	*O. japonicus*	Tuberous root (tuber)	B	O-xyl(1→3)rha(1→2)]fuc	H	H	H	H	H	glc	O-xyl(1→3)rha(1→2)]fuc	(Lee et al., 2016)
94	ophiofurospiside A	*O. japonicus*	Tuberous root (tuber)	Supplementray Figure 2									(Xu et al., 2008b)
95	ophiofurospiside B	*O. japonicus*	Tuberous root (tuber)	Supplementray Figure 2									(Xu et al., 2007)
96	ophiofurospiside C	*O. japonicus*	Tuberous root (tuber)	B	H	rha(1→2)xyl(1→4) glc	H	H	OH	H	glc(1→6)glc	-	(Liu, 2013)
97	ophiofurospiside D	*O. japonicus*	Tuberous root (tuber)	B	H	rha(1→2)glc	OH	OH	OH	H	glc	-	(Liu, 2013)
98	ophiofurospiside E	*O. japonicus*	Tuberous root (tuber)	B	H	rha(1→2)xyl(1→4) glc	H	OH	OH	H	glc	-	(Liu, 2013)
99	ophiofurospiside F	*O. japonicus*	Tuberous root (tuber)	B	H	rha(1→2)glc	H	OH	OH	H	glc	-	(Liu, 2013)
100	ophiofurospiside G	*O. japonicus*	Tuberous root (tuber)	B	H	xyl(1→4)glc	H	H	H	H	glc(1→6)glc	-	(Liu, 2013)
101	ophiofurospiside H	*O. japonicus*	Tuberous root (tuber)	B	H	xyl(1→4)glc	H	H	H	H	glc(1→	-	(Liu, 2013)

											2)glc		
102	ophiofurospiside I	*O. japonicus*	Tuberous root (tuber)	B	H	rha(1→2)glc	H	OH	H	H	glc(1→2)glc	-	(Liu, 2013)
103	ophiofurospiside J	*O. japonicus*	Tuberous root (tuber)	B	H	glc	H	H	H	H	glc(1→2)glc	-	(Liu, 2013)
104	ophiofurospiside K	*O. japonicus*	Tuberous root (tuber)	B	H	rha(1→2)glc	H	H	H	H	glc(1→6)glc	-	(Liu, 2013)
105	ophiofurospiside L	*O. japonicus*	Tuberous root (tuber)	B	OH	rha(1→2)(1→4)glc	H	H	H	H	glc	-	(Liu, 2013)
106	ophiofurospiside M	*O. japonicus*	Tuberous root (tuber)	B	OH	rha(1→2)glc	H	H	H	H	glc	-	(Liu, 2013)
107	ophiofurospiside N	*O. japonicus*	Tuberous root (tuber)	B	O-α-L-rha(1→2)β-D-xyl(1→3)β-D-fuc	H	H	H	H	H	glc	-	(Liu, 2013)
108	26-O-β-D-glucopyranosyl (25S)-furost-5-ene-1β,3β,22α,26-tetraol 1-O-β-D-xylopyranosyl- (1→3)-[α-L-rhamnopyranosyl-(1→2)]-β-D-fucopyranoside	*O. japonicus*	Tuberous root (tuber)	B	O-β-D-xyl(1→3)α-L-rha(1→2)β-D-fuc	H	H	H	H	H	glc	-	(Xu et al., 2008a)
109	26-O-β-D-glucopyranosyl-(25R)- furost-5-en-3β,14α17α,22α,26-pentaol-3-	*O. japonicus*	Tuberous root (tuber)	B	H	rha(1→2)glc	H	OH	OH	H	glc	-	(Liu et al., 2014)

	O-α-L-rhamnopyranosyl-(1→2)- β-D-glucopyranoside												
110	(25R)-26-[(O-β-D-glucopyranosy-(1→2)-β-D-glucopyranosyl)]-20α-hydro-xyfurost-5,22-diene-3-O-α-L-rhamnopyranosyl-(1→2)-[β-D-xylopyranosyl (1→4)]-β-D- glucopyranoside	*O. japonicus*	Tuberous root (tuber)	C	rha(1→2)xyl(1→4) glc	glc (1→2)glc	-	-	-	-	-	-	(Liu et al., 2014)
111	25(R, S) ruscogenin 1-O-α-L-rhamnopyranosyl-(1→2)-β-D-fucopyranoside	*L. spicata*	Fibrous root (fibrous root)	A	rha(1→2)fuc	H	H	H	H	H	H	R,S	(Qi et al., 2015)
112	(25S) ruscogenin 1-O-α-L-rhamnopyranosyl-(1→2)-β-D-rhamnopyranoside	*L. muscari*	Tuberous root (tuber)	A	O-rha (1→2)rha	H	H	H	H	H	H	S	(Do et al., 1991)
113	(25S) ruscogenin 1-O-α-L-rhamnopyranosyl(1→2)] [β-D-xylopyranosyl (1→4)]-glucopyranoside	*L. muscari*	Tuberous root (tuber)	A	O-rha(1→2)xyl (1→4)glc	H	H	H	H	H	H	S	(Li et al., 2014a)
114	(25R)-ruscogenin-1-O-α-L-rhamnopyranosyl-(1→2)-[β-D-xylopyranosyl-(1→3)]-β-D-glucopyranoside	*L. muscari*	Subterranean part (subterranean part)	A	O-rha(1→2)xyl(1→3)glc	H	H	H	H	H	H	R	(Wu et al., 2017)
115	ruscogenin l-O-[α-L-arabinopyranosyl (l-2)]-β-D-glucopyranoside	*O. intermedius*	Tuberous root (rhizome)	A	O-ara(l→2)glc	H	H	H	H	H	H	Unknown	(Rawat et al., 1988)
116		*L. muscari*	Tuberous root (tuber)	A	1-O-glc(1→2)fuc	H	H	H	H	H	H	S	(Do et al., 1991)

	25(S) ruscogenin 1-O-β-D-glucopyranosyl-(1→2)-β-D-fucopyranoside	*L. spicata*	Subterranean part (subterranean part)										(Yu et al., 1990)
117	(25S)-ruscogenin-1-O-β-D-glucopyranosyl-(1→2)-[β-D-xylopyranosyl-(1→3)]-β-D-xylopyranoside	*L. muscari*	Subterranean part (subterranean part)	A	O-glc(1→2)xyl(1→3)xyl	H	H	H	H	H	H	S	(Wu et al., 2017)
118	(25S) ruscogenin 1-O-[α-L-rhamnopyranosyl-(1→2)]-[β-D-xylopyranosyl (1→4)]-β-D-glucopyranoside	*L. muscari*	Tuberous root (tuberous root)	A	O-rha(1→2)xyl (1→4)glc	H	H	H	H	H	H	S	(Li et al., 2014a)
119	(25S)-ruscogenin 1-O-β-D-xylopyranosyl-3-O-α-L-rhamnopyranoside	*L. graminifolia*	Subterranean part (underground part)	A	O-xyl-3-O-rha	H	H	H	H	H	H	S	(Wang et al., 2011a)
		L. spicata	Subterranean part (subterranean part)	A	O-xyl(1→3)fuc	H	H	H	H	H	H	R	(Yu et al., 1990)
120	(25R)-ruscogenin 1-O-[β-D-xylopyranosyl-(1→3)]-β-D-frucopyranoside	*L. muscari*	Fibrous root (fibrous root)	A	O-xyl(1→3)fuc	H	H	H	H	H	H	R,S	(Cheng et al., 2005c)
121	(25R, S)-ruscogenin 1-O-[β-D-xylopyranosyl-(1→3)]-β-D-fucopyranoside	*L. muscari*	Tuberous root (tuberous root)	A	O-xyl(1→3)glc(1→2)fuc	H	H	H	H	H	H	R	(Li et al., 2014a)
122	(25R)-ruscogenin 1-O-[β-D-xylopyranosyl-(1→3)][β-D-glucopyranosyl-(1→2)]-β-D-fucopyranoside	*L. spicata*	Tuberous root (tuberous root)	A	H	xyl(1→3)ara(1→2)rha(1→4)glc	H	H	H	H	H	S	(Liu et al., 1989)

123	(25S)-spirost-5-ene-3β,17α-diol-3-O-[β-D-xylopyranosyl-(1→3)-α-L-arabinopyranosyl-(1→2)-[a-L-rhamnopyranosyl-(1→4)]-β-D-glucopyranosyl-(25S)-spirost-5-ene-3β,17α-diol	*L. graminifolia*	Subterranean part (underground part)	A	O-xyl-3-O-rha	H	H	H	H	H	H	S	(Wang et al., 2011a)
124	3-O-α-L-rhamnopyranosyl-1-O-sulfo-(25S)-ruscogenin	*L. graminifolia*	Subterranean part (underground part)	A	O-sulfo	rha	H	H	H	H	H	S	(Wang et al., 2011a)
125	26-O-β-D-glucopyranosyl-22-hydroxyfurost-5-ene-3β, 26-diol-3-O-α-L-rhamnopyranosyl(1→2)-β-D-glucopyranoside	*O. planiscapus*	Subterranean part (subterranean part)	B	H	rha(1→2)glc	H	H	H	H	glc	-	(Watanabe et al., 1983b)
126	26-O-β-D-glucopyranosyl-22-hydroxyfurost-5-ene-1β, 3β, 26-triol-1-O-α-L-rhamnopyranosyl (1→2)-4-O-sulfo-α-L-arabinopyranosides	*O. planiscapus*	Subterranean part (subterranean part)	B	O-rha(1→2)-4-O-sulfo-ara	H	H	H	H	H	glc	-	(Watanabe et al., 1983b)
127	yamogenin-3-O-[α-L-rhamnopyranosyl(1→2)][β-D-xylopyranosly(1→3)]-β-D-fucopyranoside	*L. spicata*	Subterranean part (subterranean part)	A	H	rha(1→2)xyl(1→3)fuc	H	H	H	H	H	unknown	(Yu et al., 1990)
128	yamogenin-1-O-[α-L-rhamnopyranosyl(1→2)][β-D-xylopyranosly(1→3)]-β-D-glucopyranoside	*L. spicata*	Fibrous root (fibrous root)	A	O-rha(1→2)(1→3)glc	H	H	H	H	H	H	unknown	(Qi et al., 2015)
129	neoruscogenin	*L. spicata*	Fibrous root (fibrous root)	D	H	-	-	-	-	-	-	-	(Qi et al., 2015)

130	neoruscogenin-1-O-[α-L-rhamnopyranosyl-(1→2)]-β-D-fucopyranoside	*L. spicata*	Fibrous root (fibrous root)	D	O-rha(1→2)fuc	-	-	-	-	-	-	-	(Qi et al., 2015)
131	neoruscogenin-1-O-β-D-glucopyranosyl-(1→2)-[β-D-xylopyranosyl-(1→3)]-β-D-xylopyranoside	*L. muscari*	Subterranean part (subterranean part)	D	O-glc (1→2)xyl(1→3)xyl	-	-	-	-	-	-	-	(Wu et al., 2017)
132	neoruscogenin-1-O-α-L-rhamnopyranosy-l-(1→2)-[β-D-xylopyranosyl-(1→3)]-β-D-glucopyranoside	*L. muscari*	Subterranean part (subterranean part)	D	O-rha(1→2)xyl(1→3)glc	-	-	-	-	-	-	-	(Wu et al., 2017)
133	neoruscogenin-1-O-β-D-glucopyranosyl-(1 -2)-[β-D-xylopyranosyl-(1-3)]-β-D-fucopyranoside	*L. muscari*	Subterranean part (subterranean part)	D	O-glc(1→2)xyl(1→3)fuc	-	-	-	-	-	-	-	(Wu et al., 2017)
134	(25S)-ruscogenin1-O-2,3-O-diacetyl-α-L-rhamnopyranosyl-(1→2)-[β-D-xylopyranosyl-(1→3)]-β-D-fucopyranoside.	*L. spicata*	Fibrous root (fibrous root)	A	H	2,3-O-diAc-rha(1→2)xyl(1→3)fuc	H	H	H	H	H	S	(Qi et al., 2015)
135	neoruscogenin-1-O-3-acetyl-[α-L-rhamnopyranosyl-(1→2)]-β-D-fucopyranoside	*L. spicata*	Fibrous root (fibrous root)	D	O-3-Ac-rha(1→2)fuc	-	-	-	-	-	-	-	(Qi et al., 2015)
136	neoruscogenin-1-O-2-O-acetyl-[α-L-rhamnopyranosyl-(1→2)]-β-D-fucopyranoside	*L. spicata*	Fibrous root (fibrous root)	D	O-2-O-Ac-rha-(1→2)fuc	-	-	-	-	-	-	-	(Qi et al., 2015)
137	Ophiopogon Saponin C1	*L. muscari*	Tuberous root (tuberous root)	A	O-glc(1→2)xyl(1→3)fuc	H	H	H	H	H	H	R	(Zhang et al., 2020)

138	spicatoside A	*L. muscari*	Unclear	A	O-glc(1→2)xyl(1→3)fuc	H	H	H	H	H	H	S	(Kwon et al., 2014)
		L. spicata	Tuberous root (tuber)										(Lee et al., 1989)
139	spicatoside C	*L. spicata*	Tuberous root (tuber)	A	fuc(1→2)xyl(1→4)fuc	OH	H	H	H	H	H	S	(Do et al., 1995)
140	fibrophiopogonins A	*O. japonicus*	Fibrous root (fibrous root)	Supplementray Figure 2									(Duan et al., 2018)
141	fibrophiopogonins B	*O. japonicus*	Fibrous root (fibrous root)	B	H	rha (1→2)]glc	H	H	H	H	glc(1→6)glc	-	(Duan et al., 2018)
142	25(R)- spirost-5,8(14)-diene-3β-ol-3-O-α-L-rhamnopyranosyl-(1→2)- [β-D-xylopyranosyl-(1→4)]-β-D-glucopyranoside	*O. japonicus*	Tuberous root (tuber)	A	H	rha(1→2)xyl(1→4) gluc	H	H	H	H	H	R	(Qi et al., 2015)
143	(20R,25R)- 26-O-b-D-glucopyranosyl-3b,26-dihydroxycholest-5-en-16,22-dioxo-3-O-a-L-rham-nopyranosyl-(1→2)-b-D-glucopyranoside	*O. japonicus*	Tuberous root (tuber)	B	H	rha(1→2)glc	H	H	H	H	glc	-	(Liu et al., 2014)
144	Ruscogenin 1-O-α-L-rhamnopyranosyl-(1 → 2)-4-O-sulfo-β-D-fucopyranosido-3-O-β-D-glucopyranoside	*O. japonicus*	Tuberous root (tuber)	A	O-rha(1 → 2)-4-O-sulfo-fuc	glc	H	H	H	H	H	R	(Wang et al., 2017b)

145	ophiojaponin A	*O. japonicus*	Tuberous root (tuber)	Supplementray Figure 2									(Wang et al., 2017b)
146	ophiojaponin B	*O. japonicus*	Tuberous root (tuber)	Supplementray Figure 2									(Wang et al., 2017b)
147	ophiojaponin C	*O. japonicus*	Tuberous root (tuber)	Supplementray Figure 2									(Wang et al., 2017b)
148	ophiojaponin D	*O. japonicus*	Tuberous root (tuber)	Supplementray Figure 2									(Wang et al., 2017b)
149	(25R)-ruscogenin 1-O-(4-O-sulfo)-β-D-fucopyranoside	*O. japonicus*	Tuberous root (tuber)	A	O-(4-O-sulfo)-fuc	H	H	H	H	H	H	R	(Chung et al., 2017)
150	pennogenin-3-O-α-L-rhamnopyranosyl-(1 → 2)-[β-D-apiofuranosyl-(1 → 4)]-β-D-glucopyranoside	*O. japonicus*	Subterranean part (root)	A	H	rha(1 → 2)api(1 → 4)]-β-D-glc	H	H	OH	H	H	unknown	(Wu et al., 2018)
151	ophiopogenin-3-O-α-L-rhamnopyranosyl-(1 → 4)-[β-D-xylopyranosyl-(1 → 3)-α-L-arabinopyranosyl-(1 → 2)]-β-D-glucopyranoside	*O. japonicus*	Subterranean part (root)	A	H	rha(1 → 4)xyl(1 → 3)ara(1 → 2)glc	H	OH	OH	H	H	R	(Wu et al., 2018)
152	pennogenin-3-O-α-L-rhamnopyranosy l-(1 → 4)-[β-D-xylopyranosyl-(1 → 3)-α-L-arabinopyranoseyl-(1 → 2)]-β-D-glucopyranoside	*O. japonicus*	Subterranean part (root)	A	H	rha(1 → 4)xyl(1 → 3)ara(1 → 2)glc	H	H	OH	H	H	R	(Wu et al., 2018)
153	prazerigenin A-3-O-α-L-rhamnopyranosyl-(1 → 4)-[β-D-xylopyranosyl-(1 → 3)-α-L-arabinopyranosyl-(1 → 2)]-β-D-glucopyranoside	*O. japonicus*	Subterranean part (root)	A	H	rha(1 → 4)xyl(1 → 3)ara(1 → 2)glc	H	OH	H	H	H	R	(Wu et al., 2018)

154	pennogenin-3-O-α-L-rhamnopyranosyl-(1 → 2)-[β-D-xylopyranosyl-(1 → 3)]-[β-D-glucopyranosyl-(1 → 4)]-β-D-glucopyranoside	*O. japonicus*	Subterranean part (root)	A	H	rha(1 → 2)xyl(1 → 3)glc(1 → 4)glc	H	H	OH	H	H	R	(Wu et al., 2018)
155	prazerigenin A-3-O-α-L-rhamnopyranosyl-(1 → 2)-[β-D-xylopyranosyl-(1 → 3)]-[β-D-glucopyranosyl-(1 → 4)]-β-D-glucopyranoside	*O. japonicus*	Subterranean part (root)	A	H	rha(1 → 2)xyl(1 → 3)glc(1 → 4)glc	H	OH	H	H	H	R	(Wu et al., 2018)
156	Liriopesides B	*L. muscari*	Unclear	A	O-rha	fuc	H	H	H	H	H	S	(Sheng et al., 2020)

(Note: [a] Since the terms applied to describe investigated plant part differ among papers and can be confusing, therefore we interpreted and unified the botanical terms in the case of liriopogons.

Specifically, 'plant part investigated' is our interpretation basing on original context, 'as stated in the original source' refers to the terms which are used in original context (see 2.3 - Terminology). [b]SO_3M refers to the sulfate of an unknown metal ion; Basic structures of different types of saponins are shown in Supplementary Figure 1)

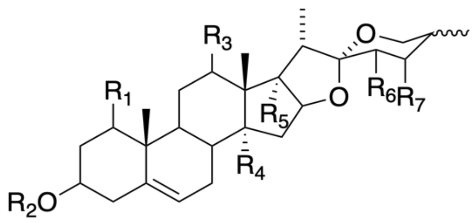

A- Spirostanol saponin

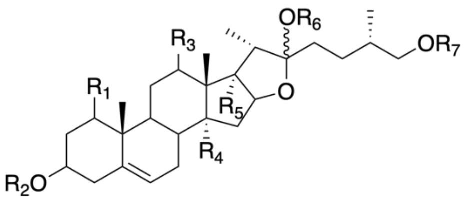

B- Furostanol saponin
(Type I)

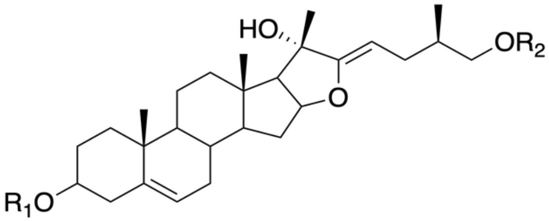

C- Furostanol saponin
(Type II)

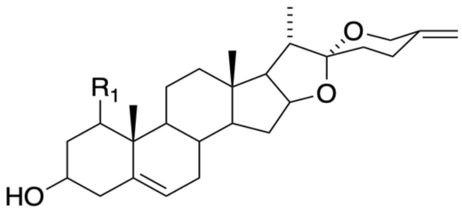

D- Neoruscogenin

Suplementary Figure 1 Basic structures of different types of saponins

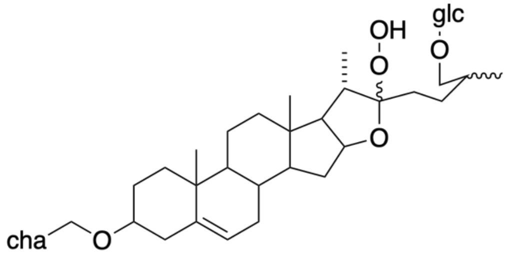

glycoside H

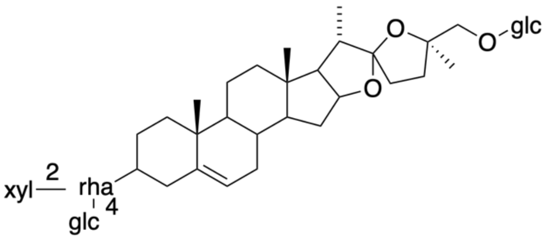

ophiofurospiside A

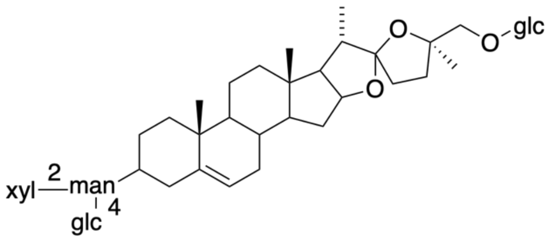

ophiofurospiside B

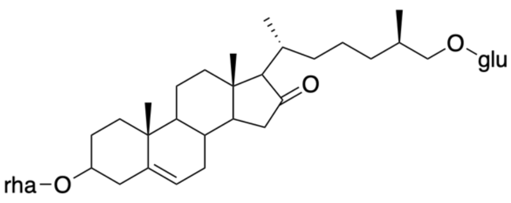

fibrophiopogonins A

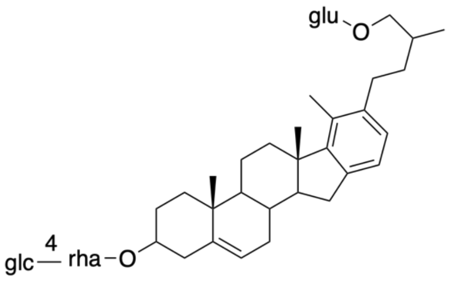

ophiojaponin A

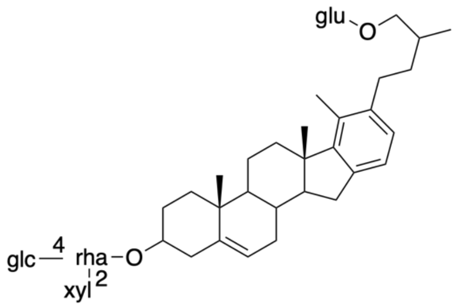

ophiojaponin B

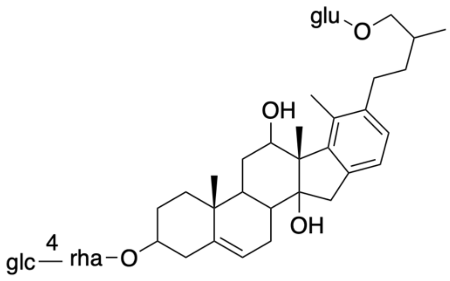

ophiojaponin C

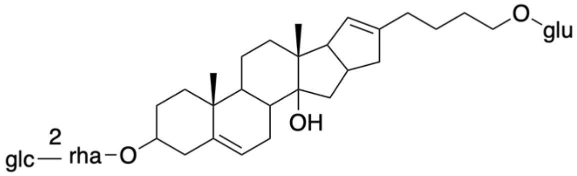

ophiojaponin D

Supplementary Figure 2 Chemical structures of other saponins from liriopogons

Supplementary Table 2 Flavonoids isolated from liriopogons

No.	Metabolites	Plant source	Plant part investigated (as stated in the original source)	Type	Structures										References
					R_1	R_2	R_3	R_4	R_5	R_6	R_7	R_8	R_9	R_{10}	
157	5,7-dihydroxy-6-methyl-3-(2′, 4′-dihydroxybenzyl) chroman-4-one	*O. japonicus*	Tuberous root (rhizome)	A	OH	H	OH	H	OH	H	OH	H	-	-	(Zhao et al., 2017)
158	4′-O-Demethylophiopogonanone E	*O. japonicus*	Tuberous root (rhizome)	A	OH	H	OH	OCH_3	OH	H	OH	H	-	-	(Zhao et al., 2017)
159	6-formylisoophiopogononeB	*O. japonicus*	Subterranean part (root)	A	OH	CHO	OH	CH_3	H	H	OCH_3	H	-	-	(Zhu et al., 1987)
160	5,7-dihydroxy-3-(4′-hydroxybenzyl)-6-methylchromone	*O. japonicus*	Subterranean part (subterranean part)	A	OH	CH_3	OH	H	H	H	OH	H	-	-	(Asanno et al., 1993a)
161	ophiopogonone A	*O. japonicus*	Subterranean part (subterranean part)	A	OH	CH_3	OH	H	H	$-O-CH_2-O-$		H	-	-	(Asanno et al., 1993a)
162	ophiopogonone B	*O. japonicus*	Tuberous root (tuber)	A	OH	CH_3	OH	H	H	H	OCH_3	H	-	-	(Tada et al., 1980a)
163	ophiopogonone C	*O. japonicus*	Tuberous root (tuber)	A	OH	OH	OH	CH_3	CHO	$-O-CH_2-O-$		H	-	-	(Chang et al., 2002)

164	ophiopogonone D	*O. japonicus*	Fibrous roots (fibrous roots)	A	OH	CH_3	OH	H	OH	H	OH	H	-	-	(Duan et al., 2009)
165	ophiopogonone E	*O. japonicus*	Fibrous roots (fibrous roots)	A	OCH_3	CH_3	OH	CHO	H	H	OCH_3	H	-	-	(Li et al., 2012a)
166	8-formyl-7-hydroxy-5,4′-dimethoxy-6-methylhomoisoflavone	*O. japonicus*	Tuberous root (root tuber)	A	OH	H	OH	H	H	H	OH	H	-	-	(Zhou et al., 2013b)
167	desmethylisoophiopogonone B	*O. japonicus*	Tuberous root (tuber)	A	OH	H	OH	H	H	H	OH	H	-	-	(Tada et al., 1980b)
168	methylophiopogonone A	*O. japonicus*	Tuberous root (tuber)	A	OH	CH_3	OH	CH_3	H	$-O-CH_2-O-$		H	-	-	(Tada et al., 1980a)
169	methylophiopogonone B	*O. japonicus*	Tuberous root (tuber)	A	OH	CH_3	OH	CH_3	H	H	H	H	-	-	(Tada et al., 1980a)
170	isoophiopogonone A	*O. japonicus*	Tuberous root (tuber)	A	OH	H	OH	CH_3	H	$-O-CH_2-O-$		H	-	-	(Tada et al., 1980b)
171	6-aldehydo-isoophiopogonone A	*O. japonicus*	Subterranean part (root)	A	OH	CHO	OH	CH_3	H	$-O-CH_2-O-$		H	-	-	(Zhu et al., 1987)
172	2′-hydroxy-methylophiopogononeA	*O. japonicus*	Tuberous root (tuber)	A	OH	CH_3	OH	CH_3	OH	$-O-CH_2-O-$		H	-	-	(Watanabe et al., 1985)
173	5,7,2′-trihydroxy-6-methyl-3-(3′,4′-methylenedioxybenzyl) chromone	*O. japonicus*	Subterranean part (subterranean part)	A	OH	CH_3	OH	H	OH	$-O-CH_2-O-$		H	-	-	(Asanno et al., 1993a)

174	5,7,2′-trihydroxy-8-methyl-3-(3′,4′-methylenedioxybenzyl) chromone	*O. japonicus*	Subterranean part (subterranean part)	A	OH	H	OH	CH_3	OH	$-O-CH_2-O-$		H	-	-	(Asanno et al., 1993a)
175	5,7,2′-trihydroxy-6,8-dimethyl-3-(3′,4′-methylenedioxybenzyl) chromone	*O. japonicus*	Fibrous roots (fibrous roots)	A	OH	CH_3	OH	CH_3	OH	$-O-CH_2-O-$		H	-	-	(Zhou et al., 2008)
176	5-hydroxy-7,8-dimethoxy-6-methyl-3-(3′,4′-dihydroxybenzyl) chroman-4-one	*O. japonicus*	Subterranean part (subterranean part)	B	OH	CH_3	OCH_3	OCH_3	H	OH	OH	OH	H	H	(Asanno et al., 1993a)
177	5,8-Dimethoxy-6-methyl-7-hydroxy-3-3(2-hydroxy-4-methoxybenzyl) chroman-4-one (58-F)	*O. japonicus*	Unclear	B	OCH_3	CH_3	OH	$O CH_3$	OH	H	OCH_3	OCH_3	H	H	(Yan et al., 2016)
178	5,7-dihydroxy-6,8-dimethyl-3-(4′-hydroxy-3′-methoxybenzyl) chroman-4-one	*O. japonicus*	Tuberous root (tuberous root)	B	OH	CH_3	OH	CH_3	H	OCH_3	OH	OH	H	H	(Nguyen et al., 2003)
179	5-hydroxy-7,8-dimethoxy-6-methyl-3-(3′,4′-dihydroxybenzyl) chroman-4-one	*O. japonicus*	Tuberous root (tuberous root)	B	OH	H	OCH_3	OCH_3	H	OCH_3	OH	OH	H	H	(Nguyen et al., 2003)
180	5,7-dihydroxy-6,8-dimethyl-3-(4′-hydroxy-3′5′-	*O. japonicus*	Tuberous root (tuberous root)	B	OH	CH_3	OH	CH_3	H	OCH_3	OH	OCH_3	H	H	(Nguyen et al., 2003)

	dimethox-ybenzyl) chroman-4-one														
181	8-formyl-5-O-methylophiopogonanoneB	*O. japonicus*	Tuberous root (root tuber)	B	OH	CH_3	OH	CHI	H	H	OCH_3	H	H	H	(Zhou et al., 2013b)
182	methylophiopogonanone A	*O. japonicus*	Tuberous root (tuber)	B	OH	CH_3	OH	CH3	H	-O-CH_2-O-		H	H	H	(Tada et al., 1980a)
183	methylophiopogonanone B	*O. japonicus*	Tuberous root (tuber)	B	OH	CH_3	OH	CH_3	H	H	OCH_3	H	H	H	(Tada et al., 1980a)
		L. graminifolia	Whole plant (whole plant)												(Wang, 2010)
184	2,5,7-trihydroxy-6,8-dimethyl-3-(4′-methoxybenzyl) chroman-4-one	*O. japonicus*	Tuberous root (tuberous root)	B	OH	CH_3	OH	CH_3	H	H	OCH_3	H	OH	H	(Nguyen et al., 2003)
185	8-formylophiopogonone B	*O. japonicus*	Tuberous root (tuber)	A	OH	CH_3	OH	CHO	H	H	OCH_3	H	-	-	(Tada et al., 1980a)
186	8-formylophiopogonanone B (8-FOB)	*O. japonicus*	Tuberous root (root tuber)	B	OH	CH_3	OH	CHO	H	H	OCH_3	H	H	H	(Zhou et al., 2013b)
187	4′-O-Demethylophiopogonanone E	*O. japonicus*	Tuberous root (rhizome)	A	OH	H	OH	OCH3	OH	H	OH	H	H	H	(Zhao et al., 2017)

188	ophiopogonanone A	*O. japonicus*	Subterranean part (root)	B	OH	CH_3	OH	H	H	-O-CH_2-O-		H	H	H	(Kaneda et al., 1983)
189	ophiopogonanone B	*L. graminifolia*	Tuberous root (tuber)	B	OH	CH_3	OH	H	H	H	OCH_3	H	H	H	(Wang et al., 2011a)
190	ophiopogonanone C	*O. japonicus*	Tuberous root (tuber)	B	OH	CH_3	OH	CHO	H	-O-CH_2-O-		H	H	H	(Chang et al., 2002)
191	Ophiopogonanone D	*O. japonicus*	Rhizomes (tubers)	B	OCH3	CH_3	OH	CHO	H	-O-CH_2-O-		H	H	H	(Chang et al., 2002)
192	ophiopogonanone E	*O. japonicus*	Tuberous root (tuber)	B	OH	CH_3	OH	OCH3	OH	H	OCH_3	H	H	H	(Chang et al., 2002)
193	ophiopogonanone F	*O. japonicus*	Tuberous root (tuber)	B	OCH3	CH_3	OH	OCH3	OH	H	OCH_3	H	H	H	(Chang et al., 2002)
194	ophiopogonanone G	*O. japonicus*	Fibrous roots (fibrous roots)	B	OH	CH_3	OH	H	OH	H	OH	H	H	H	(Duan et al., 2009)
195	ophiopogonanone H	*O. japonicus*	Fibrous roots (fibrous roots)	B	OH	CH_3	OH	CH3	H	-O-CH_2-O-		H	H	OH	(Li et al., 2012a)
196	ophiopogoside A	*O. japonicus*	Subterranean part (root)	B	OH	CH_3	O-glc	CHO	H	-O-CH_2-O-		H	H	H	(Hung et al., 2010)
197	ophiopogoside B	*O. japonicus*	Subterranean part (root)	B	OCH_3	CH_3	O-glc	CHO	H	-O-CH_2-O-		H	H	H	(Hung et al., 2010)
198	2,5,7-trihydroxy-6,8-dimethyl-3-(3′,4′-methylenedioxybenzyl)- chroman-4-one	*O. japonicus*	Subterranean part (root)	B	OH	CH_3	OH	CH3	H	H	OCH_3	H	OH	H	(Hung et al., 2010)

199	2,5,7-trihydroxy-6,8-dimethyl-3-(2′-hydroxy-3′,4′-methylene-dioxybenzyl) chromone	*O. japonicus*	Tuberous root (tuberous root)	B	OH	CH_3	OH	CH3	OH	-O-CH_2-O-		H	H	H	(Nguyen et al., 2003)
200	4',7-dihydroxy-5-methoxyflavonon e	*L. graminifolia*	Subterranean part (underground part)	C	OCH_3	H	OH	H	H	H	OH	H	H	-	(Wang et al., 2012a)
201	hesperidin	*L. graminifolia*	Subterranean part (underground part)	C	OH	H	O-rut	H	H	H	OH	OCH_3	H	-	(Wang et al., 2012a)
202	5,7-dihydroxy-8-methoxy-flavone	*L. muscari*	Fibrous roots (fibrous roots)	D	OH	H	OH	OCH3	H	H	H	-	-	-	(Li et al., 2012b)
203	(3S)3,5,4′-trihydroxy-7-methoxy-6-methylhomoisofla vanone	*L. muscari*	Fibrous roots (fibrous roots)	B	OH	CH_3	OCH_3	H	H	H	OH	H	H	OH	(Li et al., 2012b)
204	(-)-liriopeins A	*L. muscari*	Tuberous root (tuberous root)	B	OH	CH_3	OCH_3	H	H	H	OH	H	H	H	(Tsai et al., 2013)
205	(-)-liriopeins B	*L. muscari*	Tuberous root (tuberous root)	B	OH	CH_3	OCH_3	H	OH	H	OH	H	H	H	(Tsai et al., 2013)
206	(3R)-3-(4'-hydroxybenzyl)-5,7-dihydroxylchroma n-4-one	*L. muscari*	Tuberous root (tuberous root)	B	OH	H	OH	H	H	H	OH	H	H	H	(Tsai et al., 2013)
207	(3R)-3-(4'-hydroxybenzyl)-5,7-dihydroxy-6-	*L. muscari*	Tuberous root (tuberous root)	B	OH	CH_3	OH	H	H	H	OH	H	H	H	(Tsai et al., 2013)

	methyl-chroman-4-one													
208	3-(4'-hydroxybenzylidene)-5,7-dihydroxychroman-4-one	*L. muscari*	Tuberous root (tuberous root)	B	OH	CH_3	OH	H	OH	H	OH	H	H	H	(Tsai et al., 2013)
209	(3R)-3-(2',4'-dihydroxybenzyl)-5,7-dihydroxychroman-4-one	*L. muscari*	Tuberous root (tuberous root)	B	OH	H	OH	H	OH	H	OH	H	H	H	(Tsai et al., 2013)
210	(3R)-3-(2',4'-dihydroxybenzyl)-5,7-dihydroxy-6-methyl-chroman-4-one	*L. muscari*	Tuberous root (tuberous root)	B	OH	CH_3	OH	H	OH	H	OH	H	H	H	(Tsai et al., 2013)
211	(3R)-3-(4'-hydroxybenzyl)-3,5-dihydroxy-7-methoxy-6-methylchroman-4-one	*L. muscari*	Tuberous root (tuberous root)	B	OH	H	OH	H	OH	H	OH	H	H	H	(Tsai et al., 2013)
212	3,5-dihydroxy-7-methoxy-3-(4-hydroxybenzyl) chroman-4-one	*L. muscari*	Fibrous root (fibrous root)	B	OH	CH_3	OCH_3	H	H	H	OH	H	H	OH	(Wu et al., 2014)
213	3,5-dihydroxy-7-methoxy-6-methyl-3-(4-hydroxybenzyl) chroman-4-one	*L. muscari*	Fibrous root (fibrous root)	B	OH	H	OH	H	H	H	OH	H	H	H	(Wu et al., 2014)

214		*L. muscari*	Aerial part (aerial part)	C	H	H	OH	H	H	H	OH	H	H	-	(Tsai et al., 2015)
215	isoliquiritigenin	*L. muscari*	Subterranean part (root)	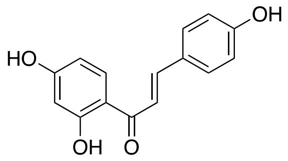											(Tsai et al., 2013)
216	3-O-methylquercetin	*L. muscari*	Subterranean part (root)	D	OH	H	OH	H	OH	OH	OCH	-	-	-	(Tsai et al., 2015)
217	3,3'-O-dimethylquercetin	*L. muscari*	Aerial part (aerial part)	D	OH	H	OH	H	OCH_3	OH	OCH	-	-	-	(Tsai et al., 2015)
218	3,4'-O-dimethylquercetin	*L. muscari*	Aerial part (aerial part)	D	OH	H	OH	H	OH	OC H3	OCH3	-	-	-	(Tsai et al., 2015)
219	kaempferol	*L. muscari*	Aerial part (aerial part)	D	OH	H	OH	H	OH	H	OH	-	-	-	(Tsai et al., 2015)
220	kaempferol-3-O-glucoside	*L. muscari*	Aerial part (aerial part)	D	OH	H	OH	H	OH	H	O-glc	-	-	-	(Tsai et al., 2015)
221	kaempferol-3-O-β-D-galactoside-4'-O-β-D-glucoside	*O. jaburan*	Fruit (seed)	D	OH	H	OH	H	OH	H	O-ga'-4'-O-glc	-	-	-	(Yoshitama et al., 1993)
222	3R-(2',4'-dihydroxybenzyl)-5,7-dihydroxy-6-methylchroman-4-one	*L. muscari*	Aerial part (aerial part)	B	OH	CH_3	OH	H	OH	H	OH	H	H	H	(Tsai et al., 2015)

223	quercetin-3-O-glucoside	*L. muscari*	Aerial part (aerial part)	D	OH	H	OH	H	OH	OH	O-glc	-	-	-	(Tsai et al., 2015)
224	isorhamnetin-3-O-glucoside	*L. muscari*	Aerial part (aerial part)	D	OH	H	OH	H	OH	OCH_3	O-glc	-	-	-	(Tsai et al., 2015)
225	diosmetin	*L. muscari*	Aerial part (aerial part)	D	OH	H	OH	H	OCH_3	OH	H	-	-	-	(Tsai et al., 2015)
226	disporopsin	*L. muscari*	Aerial part (aerial part)	C	OH	H	OH	H	OH	H	OH	H	H	-	(Tsai et al., 2015)
227	3-(2',4'-dihydroxybenzyl)-5,7-dihydroxy-6-methylchroman-4-one	*L. muscari*	Aerial part (aerial part)	B	OH	H	OH	H	OH	H	OH	H	H	H	(Tsai et al., 2015)
228	delphinidin-3-O-rutinoside	*L. muscari*	Fruit (fruit)	C	OH	H	OH	H	H	OH	OH	OH	O-rut	-	(Lee & Choung, 2011)
229	cyanidin-3-O-glucoside	*L. muscari*	Fruit (fruit)	C	OH	H	OH	H	H	H	OH	OH	O-glc	-	(Lee & Choung, 2011)
230	petunidin-3-O-rutinoside	*L. muscari*	Fruit (fruit)	C	OH	H	OH	H	H	OH	OH	OCH3	O-glc	-	(Lee & Choung, 2011)
231	petunidin-3-O-glucoside	*L. muscari*	Fruit (fruit)	C	OH	H	OH	H	H	OH	OH	OCH3	O-rut	-	(Lee & Choung, 2011)
232	malvidin-3-O-glucoside	*L. muscari*	Fruit (fruit)	C	OH	H	OH	H	H	OCH_3	OH	OCH_3	O-glc	-	(Lee & Choung, 2011)

233	6-C-methylquercetin-3-methylether	*L. muscari*	Aerial (aerial part)	D	OH	H	OH	H	OH	OH	OCH_3	-	-	-	(Tsai et al., 2015)
234	homoisopogon A	*O. japonicus*	Tuberous root (tuber)	B	OH	CH_3	OCH_3	H	OH	H	OCH_3	H	H	H	(Dang et al., 2017a)
235	homoisopogon B	*O. japonicus*	Tuberous root (tuber)	B	H	CH_3	OCH_3	H	OH	H	OCH_3	H	H	H	(Dang et al., 2017a)
236	homoisopogon C	*O. japonicus*	Tuberous root (tuber)	B	H	H	OCH_3	H	OH	H	OCH_3	H	H	H	(Dang et al., 2017a)
237	homoisopogon D	*O. japonicus*	Tuberous root (tuber)	B	H	CH_3	OH	H	H	-O-CH_2-O-		H	H	H	(Dang et al., 2017a)
238	3,4′-dimethoxy-3′,5,5′,7-tetrahydroxy-8-methylflavone	*O. japonicus*	Tuberous root (tuber)	D	OH	H	OH	CH_3	OCH_3	OH	OCH_3	-	-	-	(Chung et al., 2017)
239	5,7-Dihydroxy-6-methyl-3-(4-methoxybenzyl)-chroman-4-one	*L. graminifolia*	Stalk (stalk)	B	OH	CH_3	OH	H	H	H	OCH_3	H	H	H	(Chen et al., 2017)
240	7,4’-Dihydroxy-5-methoxyflavanone	*L. graminifolia*	Stalk (stalk)	D	OH	H	OCH_3	H	OCH_3	H	H	-	-	-	(Chen et al., 2017)

(Note: Basic structures of different types of flavonoids are shown in **Supplementar Figure** 3)

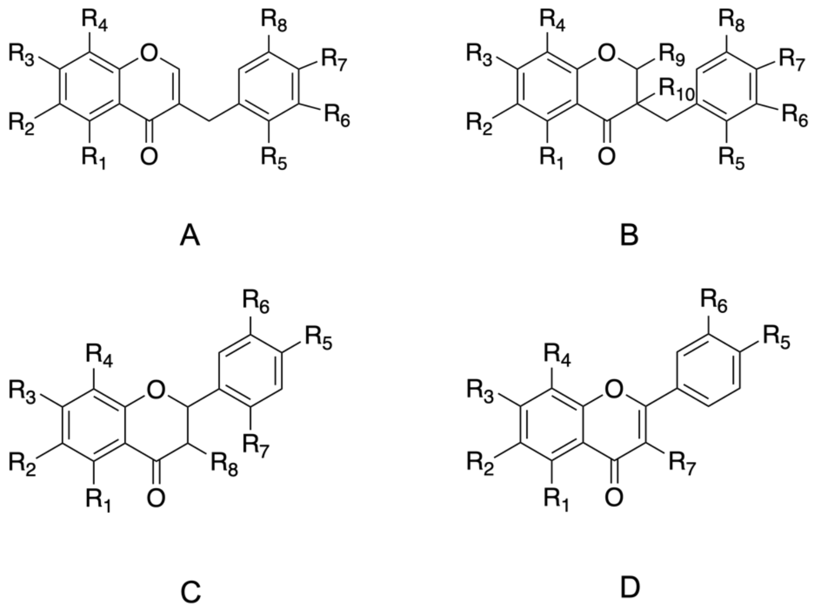

Suplementary Figure 3 Basic structures of different types of saponins

Supplementary Table 3 Polysaccharides isolated from liriopogons

No.	Metabolites	MW (kDa)	Backbone and branches	Plant sources	Plant part investigated (as stated in the original source)	References
241	Md-1	2.7064	Backbone: D-glucose and α-(1→4)glc	*O. japonicus*	Tuberous root (tuberous root)	(She & Shi, 2003)
242	Md-2	4.8651	Backbone: D-glucose and α-(1→4)glc	*O. japonicus*	Tuberous root (tuberous root)	(She & Shi, 2003)
243	MDG-1	3.4	Backbone: β-(2→1)-Frufand β-(2→6)-Fruf Branch:Fruf(2-6) Fruf(2- per average2.8 of main chain residues and trace of α-D-Glc) Fruf	*O. japonicus*	Rhizome (tuber root)	(Wang et al., 2010)

244	FOJ-5	5	Backbone: β-(2→1)-Frufand β-(2→6)-Fruf Branch:Fruf(2→6) Fruf(2- per average2.8ofmainchainresiduesandtraceof α-D-Glc) Fruf	*O. japonicus*	Tuberous root (tuberous root)	(Zheng et al., 2009)
245	Opaw-2	14	Backbone: β-(1→2)-Frufand β-(2→6) -Fruf	*O. japonicus*	Tuberous root (tuberous root)	(Wu et al., 2006)
246	OJP	4.92	Backbone composed of mainly →2)-Fruf-(6→	*O. japonicus*	Tuberous root (tuberous root)	(Gong et al., 2017)
247	OJP-1	2.74	-	*O. japonicus*	Tuberous root (tuber)	(Xiong et al., 2011)
248	OJP-2	124.3	-	*O. japonicus*	Tuberous root (tuber)	(Xiong et al., 2011)
249	OJP-3	324.65	-	*O. japonicus*	Tuberous root (tuber)	(Xiong et al., 2011)
250	OJP-4	6.75	-	*O. japonicus*	Tuberous root (tuber)	(Xiong et al., 2011)
251	POJ-U1a	4.02	1,6-α-D-glucopyranoseand1,3,6-α-D-glucofuranose Branch:1,3-α-D-glucopyranoseand1-α-D-glucopyranose	*O. japonicus*	Tuberous root (tuberous root)	(Wang et al., 2012d)
252	POJ-U1b	n.a.	→6)-α-D-Glcp (1→	*O. japonicus*	Tuberous root (tuberous root)	(Wang et al., 2016)
253	OJP1	35.2	1,6-linked glc; 1,4-linked glc; and 1,4,6-linked glc	*O. japonicus*	Tuberous root (tuberous root)	(Chen et al., 2011)

254	OJP2	88.2	(1 → 3)-linked Xyl and (1→ 6)-linked Gal	*O. japonicus*	Tuberous root (tuberous root)	(Fan et al., 2015)
255	OPF-1	48	(2 → 1)-Fruf or (2 → 6)-Fruf	*O. japonicus*	Tuberous root (tuberous root)	(Lv et al., 2012)
256	LSP	4.74	→2)-Fruf-(6→	*L. spicata*	Tuberous root (tuberous root)	(Gong et al., 2017)
257	LSP1	3.2	β-(6 → 2)-Branched β (1 → 2) fructosan	*L. spicata*	Tuberous root (tuberous root)	(Chen et al., 2009b)
258	LSP2	4.29	β-(6 → 2)-Branched β (1 → 2) fructosan	*L. spicata*	Tuberous root (tuberous root)	(Chen et al., 2009b)
259	LMP	4.14	→2)-Fruf-(6→	*L. spicata*	Tuberous root (tuberous root)	(Gong et al., 2017)

Supplementary Table 4 Organic acids and phenols isolated from liriopogons

No.	Metabolites	Plant source	Plant part investigated (as stated in the original source)	References
260	salicylic acid	*O. japonicus*	Tuberous root (tuberous root)	(Iqbal et al., 2004)
261	p-hydroxybenzoic	*O. japonicus*	Tuberous root (tuberous root)	(Iqbal et al., 2004)
262	vanillic acid	*O. japonicus*	Tuberous root (tuber)	(Cheng et al., 2005a)
		L. spicata	Fibrous root (fibrous root)	(Hu et al., 2011)
		L. muscari	Fibrous root (fibrous root)	(Cheng et al., 2005c)

263	trans-p- coumaric acid	*O. japonicus*	Tuberous root (tuber)	(Cheng et al., 2005a)
264	oleanolic acid	*O. japonicus*	Tuberous root (tuber)	(Cheng et al., 2005a)
		L. muscari	Fibrous root (fibrous root)	(Cheng et al., 2005c)
265	azelaic acid	*O. japonicus*	Tuberous root (tuber)	(Cheng et al., 2005a)
266	n-tricosanoic acid	*O. japonicus*	Tuberous root (tuber)	(Cheng et al., 2005a)
267	tianshic acid	*O. japonicus*	Tuberous root (tuber)	(Cheng et al., 2005b)
268	L-pyr- oglutamic acid	*O. japonicus*	Tuberous root (tuber)	(Hao & Wen, 2005)
269	9,12-octadecadienoic acid	*O. japonicus*	Tuberous root (tuberous root)	(Shen et al., 2008)
270	hexadecanoic acid	*O. japonicus*	Tuberous root (tuberous root)	(Shen et al., 2008)
271	6-octadecenoic acid	*O. japonicus*	Tuberous root (tuberous root)	(Shen et al., 2008)
272	tran-p-hydroxycinnamic acid	*L. spicata*	Fibrous root (fibrous root)	(Hu et al., 2011)
273	pentacosane acid	*L. muscari*	Fibrous root (fibrous root)	(Cheng et al., 2005c)
274	hentriacontane acid	*L. muscari*	Fibrous root (fibrous root)	(Cheng et al., 2005c)
275	ursolic acid	*L. muscari*	Fibrous root (fibrous root)	(Cheng et al., 2005c)
276	palmitic acid	*L. muscari*	Tuberous root (tuberous root)	(Jiang et al., 2012)
		O. japonicus	Tuberous root (rhizome)	(Zhao et al., 2017)

277	oleic acid	*O. japonicus*	Tuberous root (rhizome)	(Zhao et al., 2017)
278	syringic acid	*L. spicata*	Fibrous root (fibrous root)	(Hu et al., 2011)
279	2-(4′-hydroxybenzoyl)-5,6-methylenedioxy-benzofuran	*L. spicata*	Fibrous root (fibrous root)	(Hu et al., 2011)
280	2-(4′-hydroxybenzyl)-5,6-methylenedioxy-benzofuran	*L. spicata*	Fibrous root (fibrous root)	(Hu et al., 2011)
281	4-hydroxybenzaldehyde	*L. muscari*	Subterranean part (root)	(Tsai et al., 2013)
282	(−)-pinoresinol	*L. muscari*	Fibrous root (fibrous root)	(Li et al., 2012c)
283	(2S,3R)-methyl 7-hydroxy-2-(4-hydroxy-3-methoxyphenyl)-3-(hydroxymethyl)-2,3-dihydrobenzofuran-5-carboxylate	*L. muscari*	Fibrous root (fibrous root)	(Li et al., 2012c)
284	(4R,5S)-5-(3-hydroxy-2,6-dimethylphenyl)-4-isopropyldihydrofuran-2-one	*L. muscari*	Fibrous root (fibrous root)	(Li et al., 2012c)
285	(+)-platyphyllarin A	*L. muscari*	Subterranean part (root)	(Tsai et al., 2013)
286	platyphyllarin C	*L. muscari*	Aerial part (aerial part)	(Tsai et al., 2015)
287	(+)-Platyphyllarins B	*L. muscari*	Subterranean part (root)	(Tsai et al., 2013)
288	(2R)-(2',4'-dihydroxybenzyl)-6,7-	*L. muscari*	Aerial part (aerial part)	(Tsai et al., 2015)

	methylenedioxy-2,3-dihydrobenzofuran			
289	(2R)-(4-methoxybenzyl)-5,7-dimethyl-6-hydroxyl-2,3-dihydrobenzofuran	*O. japonicus*	Tuberous root (tuber)	(Dang et al., 2017b)
290	2-(2-hydroxyl-4-methoxy-benzyl)-5-methyl-6-methoxyl-2,3-dihydrobenzofuran	*O. japonicus*	Tuberous root (tuber)	(Dang et al., 2017b)
291	3-(2'-hydroxyphenyl)-6,8-dihydroxy-7-methoxy-isocoumarin	*L. muscari*	Aerial part (aerial part)	(Tsai et al., 2015)
292	vanillin	*L. muscari*	Subterranean part (root), aerial part (aerial part)	(Tsai et al., 2013, 2015)
293	(-)-syringaresinol	*L. muscari*	Subterranean part (root)	(Tsai et al., 2013)
294	emodin	*L. muscari*	Fibrous root (fibrous root)	(Wu et al., 2014)
295	allylpyrocatechol	*L. spicata*	Fibrous root (fibrous root)	(Hu et al., 2011)
296	2,6-dimethoxy-4-nitrophenol	*L. spicata*	Fibrous root (fibrous root)	(Hu et al., 2011)
297	N-trans-coumaroyltyramine	*L. muscari*	Fibrous root (fibrous root)	(Li et al., 2012b)
298	N-trans-feruloyloctopamine	*L. muscari*	Fibrous root (fibrous root)	(Li et al., 2012b)
299	(S)-N-trans-feruloyltyramine	*L. muscari*	Subterranean part (root)	(Tsai et al., 2013)

300	(S)-N-cis-feruloyltyramine	*L. muscari*	Subterranean part (root)	(Tsai et al., 2013)
301	(S)-N-trans-p-coumaroyloctopamine	*L. muscari*	Subterranean part (root)	(Tsai et al., 2013)
302	(S)-N-cis-p-coumaroyloctopamine	*L. muscari*	Subterranean part (root)	(Tsai et al., 2013)
303	N-transferuloyltyramine	*L. muscari*	Subterranean part (root)	(Tsai et al., 2013)
304	p-hydroxybenzaldenhyde	*O. japonicus*	Tuberous root (tuber)	(Cheng et al., 2005a)

Supplementary Table 5 Other types of constituents isolated from liriopogons

No.	Metabolites	Plant source	Plant part investigated (as stated in the original source)	References
305	L-borneol-β- D-glucopyranoside	*O. japonicus*	Tuberous root (tuber)	(Cheng et al., 2005a)
306	ophiopojaponin D	*O. japonicus*	Tuberous root (tuber)	(Hao & Wen, 2005)
307	ophiopogonoside A	*O. japonicus*	Tuberous root (tuber)	(Cheng et al., 2004)
		L. muscari	Tuberous root (tuberous root)	(Jiang et al., 2012)
308	ophiopogonoside B	*L. muscari*	Tuberous root (tuberous root)	(Jiang et al., 2012)
309	3,4-dihydroxy-allylbenzene-4-O-α-L-rhamnopyranosy(1-6)-β-D-glucopyranosideand	*O. japonicus*	Tuberous root (tuber)	(Hao & Wen, 2005)
310	liriopeoside A	*L. muscari*	Tuberous root (tuber)	(Cheng et al., 2004)

311	1,4-epoxy-cis-eudesm-6-O-β-D-glucopyranoside	*L. muscari*	Fibrous root (fibrous root)	(Zhang et al., 2011)
312	1β,6β-dihydroxy-cis-eudesm-3-ene-6-O-β-D-glucopyranoside	*L. muscari*	Fibrous root (fibrous root)	(Zhang et al., 2011)
313	1α,6β-dihydroxy-5,10-bis-epi-eudesm-4(15)-ene-6-O-β-D-glucopyranoside	*L. muscari*	Fibrous root (fibrous root)	(Zhang et al., 2011)
314	palmitic acid glyceride	*L. muscari*	Fibrous root (fibrous root)	(Zhang et al., 2011)
315	3,4-dihydroxy-allylbenzene3-O-β-d-glucopyranosyl-4-O-β-dapiofuranosyl-(1→6)-β-d-glucopyranoside	*O. japonicus*	Tuberous root (tuber)	(Liu et al., 2016)
316	3,4,5-trihydroxyallylbenzene 3-O-β-d-glucopyranosyl-4-O-β-d-glucopyranoside	*O. japonicus*	Tuberous root (tuber)	(Liu et al., 2016)
317	cryptomeridiol 11-O-β-d-xylopyranosyl-(1→6)-β-d- glucopyranoside	*O. japonicus*	Tuberous root (tuber)	(Liu et al., 2016)
318	cyclo-(Phe-Tyr)	*O. japonicus*	Tuberous root (tuber)	(Cheng et al., 2005b)
319	cyclo-(Leu-Ile)	*O. japonicus*	Tuberous root (tuber)	(Cheng et al., 2005b)
320	N-(2-(4-hydroxyphenyl)ethyl)-4-hydroxy-cinnamide	*O. japonicus*	Tuberous root (tuber)	(Nakanishi & Kaneda, 1987)
		O. planiscapus	Subterranean part (subterranean part)	(Watanabe et al., 1983b)

321	α-humulene	*O. japonicus*	Tuberous root (tuber)	(Nakanishi & Kaneda, 1987)
322	β-sitosterol	*O. japonicus*	Tuberous root (tuber)	(Kato et al., 1968)
		L. muscari	Fibrous root (fibrous root)	(Wu et al., 2014)
323	palmitic acid glyceride	*L. spicata*	Tuberous root (tuber)	(Lee et al., 1989)
324	β-sitosterol-β- D-glucopyranoside	*L. muscari*	Subterranean part (root)	(Tsai et al., 2013)
325	β-sitosterol-β- D-glucoside	*O. japonicus*	Tuberous root (tuber)	(Kato et al., 1968)
326	stigmasterol	*O. japonicus*	Tuberous root (tuber)	(Kato et al., 1968)
328	stigmasterol-β- D-glucoside	*L. muscari*	Subterranean part (root)	(Tsai et al., 2013)
329	chrysophenol	*O. japonicus*	Tuberous root (tuber)	(Cheng et al., 2005a)
330	emodin	*O. japonicus*	Tuberous root (tuber)	(Cheng et al., 2005a)
331	campesterol gulcoside	*L. muscari*	Fibrous root (fibrous root)	(Cheng et al., 2005c)
332	β- sitosterol palmitate	*L. muscari*	Fibrous root (fibrous root)	(Cheng et al., 2005c)
333	stigmasterol palmitate	*L. muscari*	Fibrous root (fibrous root)	(Cheng et al., 2005c)
334	glutaminsaeure anhydride	*L. muscari*	Fibrous root (fibrous root)	(Cheng et al., 2005c)
335	ethyltributanoate	*L. muscari*	Subterranean part (root)	(Tsai et al., 2013)
336	lupenone	*L. muscari*	Tuberous root (tuberous root)	(Jiang et al., 2007)

337	lupeol	*L. muscari*	Tuberous root (tuberous root)	(Jiang et al., 2007)

Supplementary Table 6 Summary of all retrieved pharmacological findings

Activity	Plant resource	Metabolite tested pharmacologically	Model	Effect	Dosage	Reference
Cardiovascular protection	*O. japonicus*	Steroidal saponins extract	DOX-induced<br>SD rats	↓ values of LVEDP, LVESD and LVEDD;levels of IL-6, TNF-α, IL-1β, MDA; the relative activity of p38 MAPK<br>↑values of LVESP, +2dP/dtmax, –dP/dtmax, EF and FS; activities of SOD, CAT and GSH-Px	100 mg/kg (p.o.)	(Wu et al., 2019)
		ROJ-ext (Aqueous extract)	ICR mice<br>SD rats	↓ length of tail thrombus<br>↓ arterial-venous shunt	12.5 and 25.0 mg/kg;<br>6.25 and 12.5 mg/kg (p.o.)	(Kou et al., 2006)
		ROJ-ext (Ethanol extract)	SD rats<br>HL-60 cells and ECV304 cells	↓ the dried weight of thrombus (36.0% and 70.6%); endothelium injury, adherent or transmigrated leukocytes<br>↓ adhesion of HL-60 cells to ECV304 cells	12.5 and 25.0 mg/kg (p.o.);<br>0.1, 1.0 and 10 μg/mL	(Kou et al., 2005b)
		Ruscogenin	(MCAO/R)-injured mice	↓ infarct size; brain water; ICAM-1, iNOS, COX-2, TNF-α, IL-1β; NF-κB p65 and phosphorylation<br>↑ neurological deficits	5 and 10 mg/kg (i.g.)	(Guan et al., 2013)
		Ruscogenin	(MCAO/R)-injured mice	↓ brain infarction and edema, EB leakage<br>↑ neurological deficits, cerebral brain flow CBF, ameliorated histopathological damage; expression of TJs	10 mg/kg (i.g.)	(Cao et al., 2016)

			OGD/R-injured bEnd.3 cells	↓ sodium fluorescein leakage, expression of TJs, IL-1β and caspase-1, NLRP3 and TXNIP<br>↑ cell viability and TEER value	0.1–10 μM	
		Ophiopogonin D	H9c2 cells<br>C57BL/6J mice	↓ LC3-II/LC3-I ratio, activation of JNK and ERK in H9c2 cells<br>↓ DOX-induced cardiac dysfunction in mice	1 μM<br>10 mg/kg (i.p.)	(Zhang et al., 2015c)
		Ophiopogonin D	H9c2 cells<br>Ang II-induced H9c2 cells	↑ CYP2J3 expression and 14,15-DHET levels in normal H9c2 cells<br>↓ angiotensin II-induced abnormalities in Ca^{2+} homeostasis, ER stress	100, 250 and 500 nM	(You et al., 2016)
		Ophiopogonin D	DOX-induced H9c2 cell<br>DOX-induced rats	↓ ROS accumulation and up-regulation of ERS related proteins<br>↓ cardiac ultrastructural abnormalities in rats	1 μM<br>10 mg/kg (i.p.)	(Meng et al., 2014)
		Ophiopogonin D	Ang II-infused H9c2 cells<br>Ang II-infused rats	↓ ANP, BNP, β-MHC, p-IκBα, p-REL-A, and REL-A proteins<br>↑ LVESD and LVEDD	0.1, 0.25, and 0.5 μM<br>5 or 10 mg/kg (i.p.)	(Wang et al., 2018)
		Ophiopogon D;<br>Metabolite 53 (saponin)	HUVECs	↑ the tube formation in HUVECs	10 and 100 μg/mL	(Lan et al., 2013)
		DT-13	Rat ventricular myocytes	↓ cardiac intracellular Ca^{2+}<br>↑ current voltage curve	0.1 μM	(Tao et al., 2005)

		MDG-1	HMEC-1<br><br>SD rats	↓ cell death<br>↑ cell migration and tube formation; expression of SPHK1, S1P1, bFGF, Akt, ERK, eNOS phosphorylation; NO production<br>↓ average infarct size and cells damage induced by acute myocardial ischemia<br>↑ neovascularization in chemic myocardium	0.4, 1.2, 4 and 10 mM<br>30 mg/kg (i.v.)	(Wang et al., 2010)
		MDG-1	HMEC-1	↑ intracellular S1P generation, SPHK, autocrine and paracrine stimulation of cell surface S1P receptors	0-10 mM	(Wang et al., 2012c)
		Methylophiopogonanone A (MONA)	MCAO-induced rats<br>ODG/R -induced bEND.3 cells<br>THP-1 cells	↓ infarct volume and brain edema, body weight decreases, ROS production, MMP-9 release, ICAM-1 and VCAM-1 expression<br><br>↑ neurological deficit scores, survival time, TJ	1.25, 2.50 or 5.00 mg/kg (i.v.)<br>2.5, 5.0 or 10 µM	(Lin et al., 2015)
		Methylophiopogonanone A (MONA)	I/R-induced mice<br>H/R-induced H9C2 cells	↓ infarct size (by 60.7%) and myocardial apoptosis (by 56.8%), cell apoptosis and cleaved caspase-3 expression<br><br>↑ cardiac function; PI3K, p-Akt, p-eNOS, Bcl-2/Bax ratio and restored NO production	10 mg/kg (p.o.)<br>10 µM	(He et al., 2016)
		Methylophiopogonanone B (MONB)	H_2O_2-induced HUVECs	↓ production of MDA and ROS, H_2O_2-induced apoptosis, p22phox<br><br>↑ SOD activity	10, 20, 40 and 50 µM	(Wang et al., 2019)
		OJP1	ISO-induced myocardial ischemia rats	↓ ISO-induced ST-segment elevation and the heart index; levels of marker enzymes (AST, LDH, CK and CK-MB)<br>↑ ATPases.	100, 200 and 300 mg/kg (i.g.)	(Fan et al., 2020)

		OJP1	STZ-induced diabetic rats	↓blood glucose level<br>↑ insulin level and remediating destruction of pancreatic	150 and 300 mg/kg (i.g.)	(Chen et al., 2011)
	L. muscari	DT-13	C57BL/6 mice<br><br>HUVECs	↓ ROS, TNFR, IL-8, MCP-1 and NO (dose dependent)<br>↓ NO production, phosphorylation of endothelial NO synthase	4 mg/kg (i.v.)<br><br>0.01, 0.1, 1 μM	(Fan et al., 2018)
		DT-13	Sprague-Dawley rats	↓ mRNA expression levels of IL-6 and TF	1.0, 2.0 and 4.0 mg/kg (p.o.)	(Tian et al., 2013)
		DT-13	HUVECs	↓ cleaved caspase-3 and cleaved PARP<br><br>↑ mitochondrial membrane potential, Akt phosphorylation	1, 2, 5 μM	(Qiu et al., 2014)
		Metabolite 209 and 210 (homoisoflavonoids)	Plates	↓ platelet aggregation at IC50 value of 11.59 and 10.69 μM	-	(Tsai et al., 2013)
Anti-inflammatory effects	*O. japonicus*	ROJ-ext (Aqueous extract)	ICR mice and SD rats<br><br>HL-60 and ECV304 cells	↓ ear swelling, pawedema, pleural leukocyte migration, peritoneal total leukocyte and neutrophil migration<br><br>↓ adhesion of HL-60 cells to ECV304 cells, with IC50 of 42.85 μg/mL	25 and 50 mg/kg (p.o.)<br><br>-	(Kou et al., 2005a)
		Ruscogenin	LPS-induced mice	↓ lung wet/dry weight ratio, LPS-induced MPO activity and nitrate/nitrite content; expression of TF, iNOS, procoagulant activity; NF-κB p-p65	0.3, 1.0 and 3.0 mg/kg (p.o.)	(Sun et al., 2012)
		Ruscogenin	MCT-rats	↓ endothelial cell apoptosis<br>↑ expression of eNOS, caveolin-1, and CD31	0.1, 0.4 and 0.7 mg/kg (p.o.)	(Bi et al., 2013)

		Ophiopogonin D	TNF-α- inflamed HaCaT cell;<br><br>DNCB-treated mice	↓ spleen/body weight ratio; TNF-α, IL-4, and IL-5; p38 and ERK protein activation and NF-κB nuclear translocation	1 and 10 μM;<br>125 and 250 nM	(An et al., 2020)
		Ophiopogonin D	MLE-12	↓ TNF-α, IL-1β, IL-6 and IL-8; translocation of NF-κB p65 from the cytoplasm to the nucleus; the phosphorylation of NF-κBp65<br><br>↑ AMPK activity	0-320 μM	(Wang et al., 2020b)
		DT-13	HUVECs<br>THP-1<br>TNF-α induced mice	↓ vascular inflammation, expression of ICAM-1 and VCAM-1; NF-κB p65 phosphorylation, p38 phosphorylation and Src degradation	0.01, 0.1and 1 μM;<br>4 mg/kg (i.g.)	(Zhang et al., 2015b)
		4'-O-Demethylophiopogonanone E	LPS-induced RAW 264.7 macrophage cells	↓ production of NO with IC50 value of 80.2 μg/mL; production of IL-1β and IL-6 with the IC50 value of 32.5 μg/mL and 13.4 μg/mL, respectively	0-50 μg/mL	(Zhao et al., 2017)
		Methylophiopogonone A;<br>Ophiopogonone E;<br>Methylophiopogonanone B;<br>Ophiopogonanone H	LPS-induced murine microglial cell line BV-2	↓ NO production with IC50 of 19.2, 14.4, 7.8 and 20.1 μM, respectively	-	(Li et al., 2012a)
		Ophiopogonanone G;<br>Ophiopogoside A;<br>Ophiopogoside B	human bronchial epithelial BEAS-2B cell line	↓ IL-4-induced eotaxin production and eotaxin expression	25.0 μM	(Hung et al., 2010)
		MDG-1	HUVECs	↓ Bax/Bcl-2 protein ratio, caspase-3, TNF-α, IL-1β, IL-6 and Cox-2	5, 10 or 50 mM	(Li et al., 2017)

		Metabolite 289;<br>Metabolite 290 (phenols)	LPS-induced RAW 264.7 cells	↑ LPS-induced NO production in RAW264.7 cells with the value of IC50 11.4 and 29.1 μM, respectively	-	(Dang et al., 2017b)
	L. muscari	Aqueous extract	OVA-induced mice	↓ IL-5, IL-13, IL-4 and IgE levels in the BALF and serum; expression of eosinophil CCR3 and CD11b in lung cells	150 mg/kg (i.g.)	(Lee et al., 2005)
		DT-13	Mice;<br>HL-60/ECV304	↓ acute paw edema induced by histamine in mice;<br>↓ adhesion of HL-60 to ECV304 cells induced by TNF-α or PMA	4.6 mg/kg (p.o.)<br>0.01, 0.1 and 1 μM	(Tian et al., 2011)
		Spicatoside A;<br>Ophiopogonin D	NCI-H292 cells	↑ PMA-induced mucin production secretion from airway epithelial cells	1, 10 and 100 μM	(Park et al., 2014)
	L. spicata	Metabolite 279, 280 (phenols)	Neutrophils	↓ neutrophil respiratory burst stimulated by PMA with IC50 value of 5.96 and 4.15 μM, respectively	-	(Hu et al., 2011)
Effects on the endocrine system	*O. japonicus*	Methylophiopogonanone A	HFD-induced obese rat model	↓ expression of ACC and SREBP-1C<br><br>↑ activities of lipoprotein lipase and hepatic lipase in serum and liver; expression of LDLR and PPAR α	10 mg/kg (i.g.)	(Li et al., 2020)
		Ruscogenin	STZ-induced diabetic rat	↓ macrophage influx, expression of TNF-α, IL-6 and IL-1β	3.0 mg/kg (p.o.)	(Lu et al., 2014)
		Ophiopogonin D	HFD male mice	↓ Firmicutes/ Bacteroidetes ratios and endotoxin-bearing Proteobacteria levels	1 mg/kg (i.g.)	(Chen et al., 2018b)
		Ophiopogonin D	STZ-induced DN rats	↑ serum albumin and creatinine clearance, serum creatinine, blood urea nitrogen, kidney hypertrophy; TGF-β1, and, GSH, SOD, CAT	2.5, 5 and 10 mg/kg (p.o.)	(Qiao et al., 2020)

				↓ MDA, IL-6, IL-1β		
		Polysaccharides	Gestational diabetes mellitus rat	↓fasting blood glucose and serum insulin level<br>↑APN mRNA in fat tissue and placenta.	125, 250 and 500 mg/kg (i.g.)	(Wang, 2013)
		Polysaccharides	NIT-1cells;<br>hepatoma H4IIE cell;<br>3T3-L1 mouse adipocytes;<br>BBMV assay	↓glucose absorption and activity of α-glucosidase<br><br>↑activity of NIT-1 cells	0.06-240 mg/mL	(Ding et al., 2012)
		MDG-1	ob/ob mouse model	↑fed blood glucose levels (13.5%,23.7%) and the fasting blood glucose levels (8.8%,16.3%)<br>↑serum insulin levels and TG	150 and 300 mg/kg (i.g.)	(Xu et al., 2011)
		MDG-1	diabetic KKAy mouse	↓ TG and LDL-C; expression of GSK3β.<br>↑ HDL-C content; expression of PI3-Kp85, Akt, InsR, IRS-1 and Glut-4	300 mg/kg	(Wang et al., 2012b)
		MDG-1	HFD-induced obesity mice	↓ weight and adipose tissue mass (by up to ~50%); leptin secretion, hepatic lipid accumulation; expressions of genes related to lipid and energy metabolism in the liver<br>↑ oxygen consumption and energy expenditure ameliorate plasma lipidprofiles	300 mg/kg	(Wang et al., 2014)
		MDG-1	Diabetic KKay mice	↓intestinal glucose absorption, and glycogenolysis<br>↑liver glycogenesis; GLP-1 secretion	300 mg/kg	(Zhu et al., 2014)

		MDG-1	Diet-induced obese mice	↓body weight gain, fed blood glucose levels, OGTT, and the insulin resistance	300 mg/kg	(Li et al., 2014b)
		MDG-1	HFD -induced obese C57BL/6 mice	↓ the ratio of Firmicutes/Bacteroidetes; level of D-galactosamine<br>↑level of taurine, SCFAs	300 mg/kg	(Shi et al., 2015)
		OJP	HepG2 cells and 3T3-L1 cells	↑expression of PI3K, AKT, InsR, PPAR and glucose consumption<br>↓expression of PTP1B in mRNA level and protein level in IR HepG2 cells	100, 200 and 400 mg/mL	(Gong et al., 2017)
		OJP1	STZ-induced diabetic rats	↓blood glucose level<br>↑the insulin level and remediating destruction of pancreatic islets	150 and 300 mg/kg	(Chen et al., 2011)
		OJP1	STZ-induced diabetic rats	↓MDA, TG, TC, LDL-C, HDL-C and expression of CTGF<br>↑ activity of both GPx and SOD in the serum, liver and kidneys	150 and 300 mg/kg	(Chen et al., 2013b)
		OJP1	STZ-induced diabetic rats	↓MDA, levels of AGE, hs-CRP, sICAM-1, NO and ET-1<br>↑GPx, CAT and SOD in heart of diabetic rats; eNOS mRNA level	100, 200,300 mg/kg (p.o.)	(Zhang et al., 2016a)
	L. muscari	LMP	HepG2 cells and 3T3-L1 cells	↑expression of PI3K, AKT, InsR, PPARγ and glucose consumption<br>↓expression of PTP1B in IR HepG2 cells	100, 200 and 400 mg/mL	(Gong et al., 2017)
	L. spicata	LSP	HepG2 cells and 3T3-L1 cells	↑expression of PI3K, AKT, InsR, PPAR and glucose consumption<br>↓expression of PTP1B in mRNA level and protein level in IR HepG2 cells	100, 200 and 400 mg/mL	(Gong et al., 2017)

		LSP1, LSP2	Type 2 Diabetic Mice	↓ fasting blood glucose, TC, TG, LDL-C, HDL-C/TC<br>↑ glucose tolerance, insulin resistance	100 and 200 mg/kg (p.o.)	(Chen et al., 2009a)
		Aqueous ethanol extract	STZ-diabetic rats	↓ creatinine clearance, ICAM-1, MCP-1, and fibronectin protein, TNF- α and IL-1β<br>↑ histological architecture, blood urea nitrogen and proteinuria	100 or 200 mg/kg (p.o.)	(Lu et al., 2013)
		LSP1, and LSP2	KKAy diabetic mice	↓ fasting blood glucose, lipid accumulation, hepatic gluconeogenesis<br>↑ insulin resistance and serum lipid metabolism, glycolysis and hepatic glycogen content; expression of InsR, IRS-1, phosphatidylinositol 3-kinase, and PPAR γ	100 and 200mg/kg (i.g.)	(Liu et al., 2013)
		Total Liriope spicata polysaccharides (TLSP which mainly comprised of two fractions (LSP1 and LSP2))	STZ-induced rats	↓ G6Pase activity<br>↑ glucokinase activity	100, 200 and 400 mg/kg	(Xiao et al., 2014)
		Total Liriope spicata polysaccharides (TLSP)	HFD- induced hyperlipidemia mice	↓ TC, TG, LDL-C, expressions of PPARγ and FAS, MDA content, ALT and AST level, total bile acid level<br>↑ T-AOC and SOD and GPx activities, PPARα, SHP, FXR, LXRα, LXRβ, CYP7A1, CYP51, LDL-R, APOE and HMGCR	200, 400, and 800 mg/kg	(Liu et al., 2020)
Immunomodulation	*O. japonicus*	OJP-1, OJP-2, OJP-3, and OJP-4	macrophages cell	↑ phagocytic capacity, energy metabolism rate, NO and IL-1 production	100-400 µg/mL	(Xiong et al., 2011)
		Polysaccharides	C57BL/6 mouse	↓ SMG index, spleen index, IFN-γ level and IFN-γ/IL-4 ratio<br>↑ salivary flow, body weight; water intake	50 and 100 mg/kg (i.g.)	(Wang et al., 2007)
		Saponins	Peritoneal macrophage	↑ phagocytic capacity, macrophage activity, NO production and 1L-1production	100, 200, 400 µg/mL	(Xiong et al., 2012)

	L. muscari	DT-13, ruscogenin	ICR mice; nonparenchymal cells; hepatocytes and spleen cells	↓ ALT level, hepatocelluar necrosis and adipose degeneration<br>↓ release of ALT innonparenchymal cells with IC50 of 6.3×10^{-10} M and 3.9×10^{-7} M, lympho proliferation	10 or 20 mg/kg (i.p.);<br>-;<br>10^{-5}-10^{-4} μM	(Wu et al., 2001)
		Liriope muscari polysaccharides	mouse	↑ phagotrophy function, chemotactic activity, secretion of TNF-α and IL-6	62.5, 125, 250, 500 μg/mL	(Liu et al., 2015)
		Water extract	LPS-induced mouse	↓ NO, IL-6, IL-10, IL-12p40, IP-10, KC, MCP-1, VEGF, GM-CSF, PDGF-BB, intracellular calcium, NF-κB and CREB	25-200 μg/mL	(Kim et al., 2012)
Anti-oxidation	*O. japonicus*	Ophiopogonin D	HUVECs	↓ H_2O_2-induced oxidative stress, apoptosis and ERK1/2 activation	0.6 to 60.0 μM	(Qian et al., 2010)
		Ophiopogonin D	MC3T3-E1cells and RAW264.7 cells;<br>BALB/c female mice	↓ induced MC3T3-E1 dysfunction, H_2O_2-induced MC3T3-E1 dysfunction<br>↓ CTX-1, TRAP activities, MDA, ROS generation, expression of β-catenin, mRNA expressions of Axin2 and OPG	1, 10,100 μM<br>5 and 25 mg/kg (i.p.)	(Huang et al., 2015)
		8-formylophiopogonanone B (FOB-8)	PQ-induced mice	↓ PQ-induced elevation in MDA, GSH and SOD levels	20 mg/kg (i.g.)	(Qian et al., 2019)
Cytotoxicity	*O. japonicus*	Ophiopogonin B	NCI-H157 and H460 cells	↓ H157and H460 cells with IC50 value of 2.86 and 4.61 μM, respectively	-	(Chen et al., 2013a)

		Ophiopogonin B	SGC-7901 cells	↓ cells proliferation, expression levels of Bcl-2 and the phosphorylation levels of ERK 1/2 and JNK 1/2<br>↑ expression levels of caspase-3 and B-cell lymphoma 2-associated X protein	5, 10 and 20 μM	(Zhang et al., 2016b)
		Ophiopogonin B	lung cancer<br>A549, HCC-15 and Calu-3 cells	↓ expression of c-FLIP, p62<br>↑ TRAIL-induced apoptosis by activating autophagy flux; conversion of LC3-I to LC3-II	0, 2.5, 5, and 10 μM	(Nazim et al., 2018)
		Ophiopogonin D	AMC-HN-8 cells	↓ cell proliferation, cyclin B1 and MMP-<br>↑ caspase-3/9 activity, p-p38 MAPK protein expression	0, 12.5, 25 and 50 μmol/l	(Yan et al., 2019)
		Ophiopogonin D	MDA-MB-231cells	↓ cell metastasis, MMP-9 activity, phosphorylation of FAK, Src and AKT<br>↑ nuclear β-catenin	0 to 50 μM	(Zhu et al., 2020)
		Ophiopogonin D	MDA-MB-435 cells<br>HUVECs	↓ cell proliferation, invasion, adhesion, MMP-9 and phosphorylation of p38	5, 10, 20, 40 and 80 μM	(Zhang et al., 2015a)
		Ophiopogonin D'	LNCaP, PC3 and DU145 cells	↓ LNCaP cell proliferation with IC50 values of 5.34 μM<br>↑ RIPK1- and MLKL-dependent necrosis in LNCaP cells<br>↓expression of FasL, AR and PSA	2.5 or 5 μM	(Lu et al., 2020)
		Ophiopogonin Q<br>sprengerin C	HepG2, HLE, BEL7402, BEL7403 and Hela cells	↓ HepG2, HLE, BEL7402, BEL7403 and Hela with ICH50 value of 2.88, 2.61, 3.59, 6.25, 2.74, μM, respectively<br>↓ HepG2, HLE, BEL7402, BEL7403 and Hela with ICH50 of 3.07, 3.68, 8.13, 1.97, 1.74μM, respectively	-	(Li et al., 2013)

		(−)-Liriopein B	MDA-MB-231 and MCF-7 cells	↓ MDA-MB-231 and MCF-7 at IC50 value of 83.76 and 50.25 μM, respectively<br>↓ cell migration and invasion in MDA-MB-231 cells<br>↓ PI3K, Src, EGFR, Tie2, lck, lyn, RTK5, FGFR1, Abl, and Flt	-<br>5, 10, 20 μM<br>10 μM	(Wang et al., 2015)
	L. muscari	Liriopesides B	H460 and H1975 cells	↓ cell viability and proliferation, Bcl-2 and Bcl-xl expression<br>↑ Bax, caspase-3, and caspase-8 expression, autophagy	0, 20, 40 and 60 μM	(Sheng et al., 2020)
		DT-13	A549 cells<br>HUVECs	↓ proliferation and adhesion of A549 cells to HUVECs, fibronectin, invasion, expression of MMP-2 and MMP-9	1-100 μM<br>10 and 30 μM	(Zhang et al., 2012b)
		Metabolite 17	MDA-MB-435, 95D, HepG2, HeLa, MCF-7 and A549 cells	↓ MDA-MB-435, 95D, HepG2, HeLa, MCF-7 and A549 cell lines with IC50 of 16.34, 14.34, 27.10, 14.76, 35.21 and24.69 μM, respectively	-	(Wu et al., 2017)
		Metabolite 63 (saponin), Metabolite 64 (saponin) and DT-13	MDA-MB-435 cells	↓ MDA-MB-435 cells with IC50 values of 0.58, 0.05, and 0.15 μg/mL, respectively	-	(Li et al., 2015)
		Metabolite 65, 66, 114, 117, 131, 132, 133 (saponins)	MDA-MB-435, 95D, HepG2, HeLa, MCF-7 and A549 cells	↓ MDA-MB-435, 95D, HepG2, HeLa, MCF-7 and A549 cell lines with IC50 values of<br>26.01, 30.00, 40.52, 33.42, 39.12, 36.01 mM, respectively;<br>15.99, 20.13 ,49.68,39.98,47.30,36.35 mM, respectively;<br>19.63, 10.82, 15.26, 35.56 mM, respectively;<br>18.07, 20.13, 49.68, 39.98, 47.30 and 36.35 mM, respectively;	-	(Wu et al., 2017)

				17.68, 17.83, 29.48, 22.23, 42.16 and 43.20 mM, respectively;<br><br>17.68, 17.83, 29.48, 22.23, 42.16 and 43.20 mM, respectively;<br><br>↓ MDA-MB-435, 95D, HeLa cell lines with IC50 values of 17.68, 17.83, 29.48, 22.23, 42.16 and 43.20 mM, respectively;<br><br>↓ MDA-MB-435, 95Dcell lines, IC50 with IC50 values of 17.54 and 11.09 mM, respectively		
	L. spicata	Liriopesides B	A2780 cells	↓ growth curve, CA125 level, AKP activity	1, 10^1,10^2, 10^3, 10^4 ,10^5 nM	(Wang et al., 2017a)
	L. graminifolia	Metabolite 6 (saponin)	K562 and HL60 cells	↓K562 and HL60 cells with IC50 values of 18.6 μg/mL and 16.5 μg/mL, respectively	-	(Wang et al., 2012a)
		Metabolite 1, 5, 7	HeLa cells	↓ HeLa cell with IC50 values of 6.0, 13.3 0, and 26.1 μg/mL, respectively	-	(Chen et al., 2017)
		Metabolite 40 and 124 (saponins)	SMMC-7721 and Hela cell	↓Hela with IC50 of 18.6 μg/mL and 40.6 μg/mL, respectively	2.5–100 μg/mL	(Wang et al., 2011a)
		119 (saponin), 123 (saponin) and methylophiopogonanone B	SMMC-7721 and Hela cell	↓SMMC-7721 and Hela cell with IC50 of 45.8 and 13.3 μg/mL, respectively;<br><br>Hela and SMMC-7721 cells with IC50 of 76.4 and 26.1 μg/mL, respectively	2.5–100 μg/mL	(Wang et al., 2011a)
Anti-cancer	*O. japonicus*	Ophiopogonin B	NSCLC<br><br>A549 and NCI-H460 cells; pulmonary	↓phosphorylation of EphA2 (Ser897) in A549 cells; tube formation in EA. hy926 cells; expression of VEGFR2 and Tie-2; phosphorylation of Akt (S473) and PLC (S1248); levels of	10 μM<br><br>75 mg/kg (p.o.)	(Chen et al., 2018a)

			metastasis nude mouse model	EphA2 and phosphorylated EphA2; invasion and migration ability of A549 cells<br><br>↑expression of Ephrin-A1 in both A549 and NCI-H460 cells; phosphorylation of EphA2 (Ser897) in NCI-H460 cells<br><br>↓EphA2, N-cadherin, Snail, Slug and ZEB1tumor angiogenesis and hemoglobin content<br><br>↑ZO-1 and E-cadherin		
		Ophiopogonin B	A549 cells;<br>nude mouse model implanted with A549 cell	↓ expression of Myt1 and phosphorylation of Histone H3 (Ser10)<br>↑ autophagy and apoptosis (75 mg/kg OP-B)	10 μM<br>75 mg/kg (p.o.)	(Chen et al., 2016b)
		Ophiopogonin D'	PC3 and DU145 cells;<br>BALB/c nude mice implanted with PC3 and DU145 cell s	↓ levels of cleaved-RIPK1, caspase 8, cleaved-caspase 8, Bid, caspase 10, and cleaved-caspase 10<br><br>↑ cell apoptosis, expression levels of RIPK1 and Bim<br><br>↓ PC3 and DU145 xenograft tumors in BALB/c nude mice	1, 2.5, 5, 10, 25, and 50 μM<br>2.5 or 5.0 mg/kg (i.p.)	(Lu et al., 2018)
		DT-13	MDA-MB-435 cells;<br>nude mice implanted with MDA-MB-435 cell	↓ cell adhesion to vitronectin, migratory response, expression of avb 3 integrin, TF and Egr-1, excretion of MMP-9 of MDA-MB-435 cells under hypoxic conditions<br>↓ extravasation of MDA-MB-435 cells in mice	1 or 10 mM	(Sun et al., 2010)

		DT-13	95D cells;<br>Orthotopic implantation mouse model	↓ 95D cells metastasis, expression of paxillin, p-paxillin, p-c-Raf, total c-Raf, p-ERK1/2, total ERK1/2 and β-actin<br><br>↑ non-muscle myosin IIA	0.01, 0.1 and 1 μM<br>2.5 or 10 mg/kg (i.g.)	(Wei et al., 2016)
		DT-13	HCT-15, HCT-116, COLO 205, HT-29, SW-620 and SW-480 cells;<br><br>Orthotopic implantation mouse model of colorectal cancer;<br>C57BL/6J APC^{min} mice model	↓ glucose uptake, ATP generation; lactate production; m-TOR<br>↑ AMPK<br>↓ expression of GLUT1, colorectal cancer growth	2.5, 5 and 10 μM<br>0.625, 1.25, 2.5 mg/kg (i.g.)<br>10 mg/kg (i.g.)	(Wei et al., 2019)
		Ruscogenin	SMMC-7721 and HCCLM3;<br>nude mice implanted with HCCLM3 cells	↓ cell migration and invasion; levels of MMP-2, MMP-9, urokinase-type plasminogen activator, VEGF and HIF-1α; phosphorylation of Akt, mTOR	0-100 μM;<br>0.3, 1.0, or 3.0 mg/kg (i.v.)	(Hua et al., 2018)
		Sprengerinin C	HepG-2/BEL7402 cells;<br>nude mice implanted with HepG-2 cells	↓ VEGF-induced vascular endothelial cell proliferation, invasion and tube formation; VEGFR2 activation, MMP-2/9 and VEGF expression<br><br>↑ G2/M phase arrest, NADPH oxidase activity, reactive oxygen species, cleaved caspase-3 and cleaved PARP<br><br>↓ tumor growth in a nude mouse	0.5, 1.0 and 2.0 μM;<br>7.5 and 15 mg/kg (i.p.)	(Zeng et al., 2013)

		Metabolite 26 (saponin)	HUVECs<br><br>C57/BL mice	↓ HUVECs invasion and tube formation; expression of Src tyrosine kinase<br><br>↓ angiogenesis and MMPs/VEGF expression	1.25, 2.5, 5.0 and 10.0 μM<br><br>5.0 μM (SC)	(Zeng et al., 2015)
	L. muscari	Ophiopogon Saponin C1	A549 cells;<br><br>mice	↓ cell migration<br><br>↓ degradation and breakage of the ZO-1 protein; PKCδ and Src	0.01, 0.1, 1 μM<br><br>4.0 mg/kg (i.g.)	(Zhang et al., 2020)
Anti-viral	*L. muscari*	Metabolite 207 (saponin)	HBV-transfected Huh7 cells	↓ pCore-Luc, pS-Luc, pPreS-Luc activities; binding activity of NF- κB protein to CS1 element; CS1 containing promoter activity<br><br>↓ expression of p65/p50 NF- κB protein, phosphorylated NF-κB p65	0-10 μg/mL	(Huang et al., 2014)
		Spicatoside A	human hepatocellular carcinoma cell line Huh 7.5	↓ replication of the genotype 3 HEV replicon<br>↓ HEV genotype 3 strain 47832c<br>↓ expression of HEV ORF2	0.5, 1 and 2 μg/mL;<br>2 μg/mL<br>0.2, 0.5, 1 and 2 μg/mL;	(Park et al., 2019)
Anti-tussive	*O. japonicus*	Ophiopogonin D	Paratracheal neurones	hyperpolarized the paratracheal neurones from a resting membrane potential of -65.7 to -73.5 mV	10 μM	(Ishibashi et al., 2001)
Neuroprotection	*L. muscari*	Ethanol extract	H_2O_2-induced injury in SH-SY5Y cells	↓ intracellular oxidative stress, mitochondrial dysfunction, poly (ADP ribose) polymerase and caspase-3 cleavage	0.5– 50 μg/mL	(Park et al., 2015)
Acute myeloid leukemia (anti-AML)	*L. muscari*	DT-13	Human leukemia cell lines;	↑ apoptosis of HL-60 and Kasumi-1 cells	0-18 μM;	(Wang et al., 2020a)

			NOD/SCID mice with the engraftment of HL-60 cells	↑ Fas, FasL, DR5, TRAIL, the cleaved-PARP and cleaved-caspase 3 and 8, differentiation markers CD11b and CD14, level of C/EBPα and C/EBPβ<br><br>↑ NOD/SCID mice survival time	10 and 20 mg/ kg (p.o.)	
laxative	*L. muscari*	AEtLP (aqueous extract)	operamide (Lop)-induced rats	↑ villus length, crypt layer, muscle thickness in the constipation model<br>↓ mAChRs	1,000 mg/kg (i.g.)	(Kim et al., 2013)
anti-dry eye	*L. muscari*	LPE (water extract)	PM-induced DES rats	↑ tear secretion, corneal smoothness, level of MUC4, conjunctival goblet cells	1, 5 and 10 mg/mL	(Song et al., 2019)
effect on gastrointestinal motility	*L. muscari*	LPE (water extract)	ICC, PPTs; acetic acid and STZ-induced diabetic mouse	depolarizes ICC PPTs in vitro<br><br>↑ ITRs in vivo	10–30 μg/ml<br>100-1000 mg/kg (i.g.)	(Kim et al., 2016)
anti-bronchial asthma	*L. muscari*	LPP (water extract)	EGF-induced asthma condition A549 cells; hBSM	↓ MAPK/NF-κB activity in EGF-induced asthma condition A549 cells, COX-2 and iNOS expression, PLCβ in asthmatic hBSM	0.1 to 1 mg/mL	(Lee et al., 2019)
Hepatoprotection	*O. japonicus*	58-F	CCl_4-induced mouse; H_2O_2-induced BNL CL.2 hepatocyte cell	↓ lysosome membrane permeabilization, cathepsin B, cathepsin D<br>↑ lysosomal enzyme translocation to the cytosol, fluorescence intensity of the LysoTracker Green, cell viability	15 mg/kg (i.g.)<br><br>50 μM	(Yan et al., 2016)

REFERENCES

Adinolfi, M., Parrilli, M., and Zhu, Y. X. (1990). Terpenoid Glycosides from Ophiopogon Japonicus Roots. *Phytochemistry*. 29 (5), 1696–1699.

Asanno, T., Murayama, T., Hirai, Y., and Shoji, J. (1993a). Comparative Studies on the Constituents of Ophiopogonis Tuber and its Congeners. VII. Studies on the Homoisoflavonoids of the Subterranean Part of Ophiopogon Japonicus KERGAWLER Cv. Nanus. (1). *Chem. Pharm. Bull.* 41 (2), 391–393.

Asano, T., Murayama, T., Hirai, Y., and Shoji, J. (1993b). Comparative Studies on the Constituents of Ophiopogonis Tuber and its Congeners. VIII. Studies on the Glycosides of the Subterranean Part of Ophiopogon Japonicus Ker-Gawler Cv. Nanus. *Chem. Pharm. Bull*. (Tokyo) 41 (3), 566–570. Available at: http://www.mendeley.com/research/geology-volcanic-history-eruptive-style-yakedake-volcanogroup-central-japan/.doi:10.1248/cpb.41.566

Chang, J. M., Shen, C. C., Huang, Y. L., Chien, M. Y., Ou, J. C., Shieh, B. J., et al. (2002). Five New Homoisoflavonoids from the Tuber of Ophiopogon Japonicus. *J. Nat. Prod.* 65 (11), 1731–1733. doi:10.1021/np020204o

Chen, J. J., Zhu, Z. L., and LuoDe, S. (2000). Cixi -ophiopogon A and B, C27 Steroidal Glycosides from Ophiopogon Japonicum. *Acta Bot. Yunnanica*. 22 (1), 97–102.

Chen, M. J., Cheng, H., Guo, Y. Y., Jiang, R. L. H., Jiang, H. M., Zhou, Y., et al. (2018a). Ophiopogonin B Suppresses the Metastasis and Angiogenesis of A549 Cells *In Vitro* and *In Vivo* by Inhibiting the EphA2/Akt Signaling Pathway. *Oncol. Rep.* 40, 1339–1347. doi:10.3892/or.2018.6531

Chen, M., Du, Y., Qui, M., Wang, M., Chen, K., Huang, Z., et al. (2013a). Ophiopogonin B-Induced Autophagy in Non-small Cell Lung Cancer Cells via Inhibition of the PI3K/Akt Signaling Pathway. *Oncol. Rep.* 29 (2), 430–436. doi:10.3892/or.2012.2131

Chen, M., Guo, Y., Zhao, R., Wang, X., Jiang, M., Fu, H., et al. (2016b). Ophiopogonin B Induces Apoptosis, Mitotic Catastrophe and Autophagy in A549 Cells. *Int. J. Oncol*. 49 (1), 316–324. doi:10.3892/ijo.2016.3514

Chen, M. L., Wang, W., and Wang, K.-W. (2017). Chemical Constituents of Liriope Graminifolia and Their Biological Activities. *Chem. Nat. Compd.* 53 (6), 1170–1173. doi:10.1007/s10600-017-2229-3

Chen, X., Liu, Y., Bai, X., Wen, L., Fang, J., Ye, M., et al. (2009b). Hypoglycemic Polysaccharides from the Tuberous Root of Liriope Spicata. *J. Nat. Prod.* 72 (11), 1988–1992. doi:10.1021/np900346d

Chen, X., Jin, J., Tang, J., Wang, Z., Wang, J., Jin, L., et al. (2011). Extraction, Purification, Characterization and Hypoglycemic Activity of a Polysaccharide Isolated from the Root of Ophiopogon Japonicus. *Carbohydr. Polym.* 83 (2), 749–754. doi:10.1016/j.carbpol.2010.08.050

Chen, X., Tang, J., Xie, W., Wang, J., Jin, J., Ren, J., et al. (2013b). Protective Effect of the Polysaccharide from Ophiopogon Japonicus on Streptozotocin-Induced Diabetic Rats. *Carbohydr. Polym*. 94 (1), 378–385. doi:10.1016/j.carbpol.2013.01.037

Cheng, Z. H., Wu, T., Bligh, S. W., Bashall, A., and Yu, B. Y. (2004). cis-eudesmane Sesquiterpene Glycosides from Liriope Muscari and Ophiopogon Japonicus. *J. Nat. Prod.* 67, 1761–1763. doi:10.1021/np049864e

Cheng, Z. H., Wu, T., and Yu, B. Y. (2006b). Steroidal Glycosides from Tubers of Ophiopogon Japonicus. J. *Asian Nat. Prod. Res.* 8 (6), 555–559. doi:10.1080/10286020410001721122

Cheng, Z. H., Wu, T., and Guo, Y. L. (2006a). Two New Steroidal Glycosides from Liriope Muscari. *Chin. Chem. Lett.* 17 (1), 31–34.

Cheng, Z. H., Wu, T., Li, L. Z., Liu, N., Yu, B. Y., and Xu, L. S. (2005a). Studies on the Liposoluble Components from Tuber of Ophiopogon Japonicus. *Chin. Pharm. J.* 40 (5), 337–341.

Cheng, Z. H., Wu, T., and Yu, B. Y. (2005b). Chemical Constituents in the Tubers of Ophiopogon Japonicus. Nat. Prod. Res. Dev. 17 (1), 1–3.

Cheng, Z. H., Wu, T., Yu, B. Y., and Xu, L. S. (2005c). Phytchemical Research on Liriope Muscari. Chin. Tradit. Herb. Drugs 36 (6), 823–826.

Choi, S. J., Choi, J., Jeon, H., Bae, S. K., Ko, J., Kim, J., et al. (2015). Application of High-Performance Countercurrent Chromatography for the Isolation of Steroidal Saponins from Liriope Plathyphylla. J. Sep. Sci. 38 (1), 18–24.doi:10.1002/jssc.201401007

Chung, N. D., Thao, N. P., Tuan, H. M., Thanh, N. V., Dang, N. H., Huong, N. T.M., et al. (2017). New Steroidal Glycoside and Flavonoid Constituents from Ophiopogon Japonicus. Nat. Prod. Commun. 12 (6), 905–906. doi:10.1177/1934578x1701200618

Dai, H.F., Deng, S.M., Tan, N.H., and Zhou, J. (2005). A New Steroidal Glycoside from Ophiopogon Japonicus (Thunb.) Ker-Gawl. *J. Integr. Plant Biol.* 47 (9), 1148–1152. doi:10.1111/j.1744-7909.2005.00084.x

Dai, H. F., Zhou, J., Ding, Z. T., Xiong, J., and Tan, N. H. (2000). Two New Steroidal Glycosides from Ophiopogon Japonicus. *Chinese Chem. Lett.* 11 (10), 901–904.

Dang, N. H., Chung, N. D., Tuan, H. M., Hiep, N. T., and Dat, N. T. (2017a). Cytotoxic Homoisoflavonoids from Ophiopogon Japonicus Tubers. *Chem. Pharm. Bull.* (Tokyo) 65 (2), 204–207. doi:10.1248/cpb.c16-00743

Ding, L., Li, P., Lau, C. B., Chan, Y. W., Xu, D., Fung, K. P., et al. (2012). Mechanistic Studies on the Antidiabetic Activity of a Polysaccharide-Rich Extract of Radix Ophiopogonis. *Phytother Res*. 26 (1), 101–105. doi:10.1002/ptr.3505

Do, J. C., Jung, K. Y., Sung, Y. K., Jung, J. H., and Son, K. H. (1995). Spicatoside C, a New Steroidal Saponin from the Tubers of Liriope Spicata. J. Nat. Prod. 58 (5), 778–781. doi:10.1021/np50119a022

Do, J. C., Sung, Y. K., and Son, K. H. (1991). Further Spirostanol Glycosides from the Tuber of Liriope Spicata. *Korean J. Pharmacogn* 22 (2), 73–77.

Duan, C.-L., Li, Y.-J., Li, P., Jiang, Y., Liu, J.-X., and Tu, P.-F. (2010a). Spirostanol Saponins from the Fibrous Roots ofOphiopogon japonicus(Thunb.) Ker-Gawl. *Hca* 93 (2), 227–232. doi:10.1002/hlca.200900165

Duan, C. L., Kang, Z. Y., Lin, C. R., Jiang, Y., Liu, J. X., and Tu, P. F. (2009). Two New Homoisoflavonoids from the Fibrous Roots of Ophiopogon Japonicus (Thunb.) Ker-Gawl. *J. Asian Nat. Prod. Res.* 11 (10), 876–879. doi:10.1080/10286020903093161

Duan, C., Wang, Y., Ma, X., Jiang, Y., Liu, J., and Tu, P. (2012). A New Furostanol Glycoside with Fatty Acid Synthase Inhibitory Activity from Ophiopogon Japonicusa. *Chem. Nat. Compd*. 48 (4), 613–615. doi:10.1007/s10600-012-0325-y

Duan, C. L., Li, Y. J., Wang, F. Y., Miao, L., and Tang, X. D. (2018). New Steroidal Glycosides from the Fibrous Roots of Ophiopogon Japonicus. *J. Asian Nat. Prod. Res*. 20 (8), 744–751. doi:10.1080/10286020.2018.1478819

Duan, C. L., Ma, X. F., Jiang, Y., Liu, J. X., and Tu, P. F. (2010b). Two New Furostanol Glycosides from the Fibrous Root of Ophiopogon Japonicus (Thunb.) Ker-Gawl. *J. Asian Nat. Prod. Res.* 12 (9), 745–751. doi:10.1080/10286020.2010.495332

Fan, S., Zhang, J., Xiao, Q., Liu, P., Zhang, Y., Yao, E., et al. (2020). Cardioprotective Effect of the Polysaccharide from Ophiopogon Japonicus on Isoproterenol- Induced Myocardial Ischemia in Rats. *Int. J. Biol. Macromol.* 147, 233–240. doi:10.1016/j.ijbiomac.2020.01.068

Fan, S., Wang, J., Mao, Y., Ji, Y., Jin, L., Chen, X., et al. (2015). Characterization and Antioxidant Properties of OJP2, a Polysaccharide Isolated from Ophiopogon Japonicus. *Abb* 06 (08), 517–525. doi:10.4236/abb.2015.68054

Hao, F. D., and Wen, L. M. (2005). Ophiopojaponin D, a New Phenylpropanoid Glycoside from Ophiopogon Japonicus Ker-Gawl. *Arch. Pharm. Res.* 28 (11), 1236–1238.

Iqbal, Z., Hiradate, S., Araya, H., and Fujii, Y. (2004). Plant Growth Inhibitory Activity of Ophiopogon Japonicus Ker-Gawler and Role of Phenolic Acids and Their Analogues: A Comparative Study. *Plant Growth Regul.* 43 (3), 245–250. doi:10.1023/b:grow.0000045998.68084.4b

Jiang, C., Liu, Z. H., Li, L., Lin, B. B., Yang, F., and Yang, M. J. (2012). A New Eudesmane Sesquiterpene Glycosides from Liriope Muscari. *J. Asian Nat. Prod. Res.* 14 (5), 491–495. doi:10.1080/10286020.2012.668533

Jiang, T., Huang, B. K., Zhang, Q. Y., Han, T., Zheng, H. C., and Qin, L. P. (2007). [Studies on Chemical Constituents of Liriope Platyphylla]. *Zhong Yao Cai* 30 (9), 1079–1081.

Kaneda, N., Nakanishi, H., Kuraishi, T., and Katori, T. (1983). [Studies on the Components of Ophiopogon Roots (China). I]. *Yakugaku Zasshi* 103 (11), 1133–1139. Available at: http://www.mendeley.com/research/geology-volcanichistory-eruptive-style-yakedake-volcano-group-central-japan/. doi:10.1248/ yakushi1947.103.11_1133

Kato, H., Sakuma, S., Tada, A., Kawanishi, S., and Shoji, J. (1968). [Studies on the Constituents of Ophiopogonis Tuber. I. Isolation of Steroidal Glycosides from Tuber of Ophiopogon Japonicus Ker-Gawler Var. Genuinus Maxim]. *Yakugaku Zasshi* 88, 710–714. doi:10.1248/yakushi1947.88.6_710

Kim, J. E., Lee, Y. J., Kwak, M. H., Ko, J., Hong, J. T., and Hwang, D. Y. (2013). Aqueous Extracts of Liriope Platyphylla Induced Significant Laxative Effects on Loperamide-Induced Constipation of SD Rats. *BMC Complement. Altern. Med.* 13, 333. doi:10.1186/1472-6882-13-333

Kwon, G., Lee, H. E., Lee, D. H., Woo, H., Park, S. J., Gao, Q., et al. (2014). Spicatoside A Enhances Memory Consolidation through the Brain-Derived Neurotrophic Factor in Mice. *Neurosci. Lett.* 572, 58–62. doi:10.1016/j.neulet.2014.04.034

Lan, S., Yi, F., Shuang, L., Chenjie, W., and Zheng, X. W. (2013). Chemical Constituents from the Fibrous Root of Ophiopogon Japonicus, and Their Effect on Tube Formation in Human Myocardial Microvascular Endothelial Cells. *Fitoterapia* 85 (1), 57–63. doi:10.1016/j.fitote.2012.12.025

Lee, D.Y., Son, K.H., Do, J.C., and Kang, S. S. (1989). Two New Steroidal Saponins from the Tubers of Liriope Spicata. *Arch. Pharm. Res.* 12 (4), 295–299. doi:10.1007/bf02911063

Lee, S. R., Han, J. Y., Kang, H. R., Lee, H. L., Noh, H. J., Cha, J. S., et al. (2016). A New Steroidal Saponin from the Tubers of Ophiopogon Japonicus and its Protective Effect against Cisplatin-Induced Renal Cell Toxicity. *J. Braz. Chem. Soc.* 27 (4), 706–711. doi:10.5935/0103-5053.20150319

Lee, Y. C., Lee, J. C., Seo, Y. B., and Kook, Y. B. (2005). Liriopis Tuber Inhibit OVAInduced Airway Inflammation and Bronchial Hyperresponsiveness in Murine Model of Asthma. *J. Ethnopharmacol.* 101 (1–3), 144–152. doi:10.1016/j.jep.2005.04.030

Li, N., Zhang, L., Zeng, K. W., Zhou, Y., Zhang, J. Y., Che, Y. Y., et al. (2013). Cytotoxic Steroidal Saponins from Ophiopogon Japonicus. Steroids 78, 1–7. doi:10.1016/j.steroids.2012.10.001

Li, W. J., Cheng, X. L., Liu, J., Lin, R. C., Wang, G. L., Du, S. S., et al. (2012b). Phenolic Compounds and Antioxidant Activities of Liriope Muscari. Molecules 17 (2), 1797–1808. doi:10.3390/molecules17021797

Li, W. J., Zhang, Z. H., Cheng, X. L., Liu, J., He, Y., Zhou, C., et al. (2012c). Two New Compounds Isolated from Liriope Muscari. Molecules 17 (8), 8773–8781. doi:10.3390/molecules17088773

Li, Y. W., Qi, J., Wen-Zhang, W., Zhou, S. P., Yan-Wu, Y., and Yu, B. Y. (2014a). Determination and Fingerprint Analysis of Steroidal Saponins in Roots of Liriope Muscari (Decne.) L. H. Bailey by Ultra High Performance Liquid Chromatography Coupled with Ion Trap Time-Of-Flight Mass Spectrometry. *J. Sep. Sci.* 37, 1762–1772. doi:10.1002/jssc.201400273

Li, Y. W., Qi, J., Zhang, Y. Y., Huang, Z., Kou, J. P., Zhou, S. P., et al. (2015). Novel Cytotoxic Steroidal Glycosides from the Roots of Liriope Muscari. *Chin. J. Nat. Med.* 13 (6), 461–466. doi:10.1016/S1875-5364(15)30040-6

Li, Y., Zhu, Y.-Y., Shi, L.-L., Shen, L., Wei, H.,Wang, Y., et al. (2014b). UPLC-TOF/MS Based Urinary Metabonomic Studies Reveal Mild Prevention Effects of MDG-1 on Metabolic Disorders in Diet-Induced Obese Mice. *Anal. Methods* 6 (12), 4171–4180. doi:10.1039/c4ay00796d

Liu, N.,Wen, X., Liu, J., Liang, M., Zeng, H., Lin, Y., et al. (2006). Determination of Ruscogenin in Crude Chinese Medicines and Biological Samples by Immunoassay. *Anal. Bioanal. Chem.* 386 (6), 1727–1733. doi:10.1007/s00216-006-0767-9

Liu, S. Q., Kang, L. P., Zhang, J., Liu, Y. X., Zhao, Y., Liu, B., et al. (2016). New Sesquiterpenoid Glycoside and Phenylpropanoid Glycosides from the Tuber of Ophiopogon Japonicus. *J. Asian Nat. Prod. Res.* 18 (6), 520–527. doi:10.1080/10286020.2015.1121996

Liu, W., Wang, Z. L., and Liang, H. Q. (1989). [Studies on the Chemical Constituents of Liriope Spicata Lour (Thunb), Var. Prolifera Y.T. Ma]. *Yao Xue Xue Bao* 24 (10), 749–754.

Liu, Y., Meng, L. Z., Xie, S. X., Xu, T. H., Sun, L. K., Liu, T. H., et al. (2014). Studies on Chemical Constituents of Ophiopogon Japonicus. *J. Asian Nat. Prod. Res.* 16 (10), 982–990. doi:10.1080/10286020.2014.935348

Liu, Y. G., Xu, J. H., and Zhang, H. L. (2015). Effect of Liriope Muscari Polysaccharides on Function of Macrophage in Abdominal Cavity of Mouse. *Zhongchengyao* 37 (10), 2290–2292. doi:10.3969/j.issn.1001-1528.2015.10.043

Liu, Y. H., Wan, L., Xiao, Z., Wang, J., Wang, Y., and Chen, J. (2013). Antidiabetic Activity of Polysaccharides from Tuberous Root of Liriope Spicata Var. Prolifera in KKAy Mice. *Evidence-based Complement. Altern. Med.* 2013, 349790. doi:10.1155/2013/349790

Liu, Y. H., Xiang, Z. N., Chen, C., Wan, L. S., and Chen, J. C. (2020). Hypolipidemic and Hepatoprotective Effects of Polysaccharides Extracted from Liriope Spicata Var. Prolifera in C57BL/6J Mice with High-Fat Diet-Induced Hyperlipidemia. *Evidence-based Complement. Altern. Med.* 2020, 8013189. doi:10.1155/2020/8013189

Lu, Z.,Wu, C., Zhu,M., Song, W.,Wang, H.,Wang, J., et al. (2020). Ophiopogonin D' Induces RIPK1-dependent Necroptosis in Androgen-dependent LNCaP Prostate Cancer Cells. *Int. J. Oncol.* 56 (2), 439–447. doi:10.3892/ijo.2019.4945

Lv, N., Pu, J. P., Jin, F. X., and Yu, S. H. (2012). Structural Analysis of a Polysaccharide OPF-1 from Ophiopogon Japonicus. J. *Dalian Polytech. Univ.* 31 (4), 243–246.

Ma, S., Kou, J., and Yu, B. (2011). Safety Evaluation of Steroidal Saponin DT-13 Isolated from the Tuber of Liriope Muscari (Decne.) Baily. *Food Chem. Toxicol.* 49, 2243–2251. doi:10.1016/j.fct.2011.06.022

Nakakuki, S. (1986). Comparative Studies on the Constituents of Ophiopogonis Tuber and Its Congeners. I. Studies of the Constituents of the Subterranean Part of Liriope platyphylla Wang et Tang. *Chem. Pharm. Bull.* 34 (1), 430–433.

Nakanishi, H., and Kaneda, N. (1987). [Studies on the Components of Ophiopogon Tuber (China). II]. *Yakugaku Zasshi* 107 (10), 780–784. Available at: http://www.mendeley.com/research/geology-volcanic-history-eruptive-style-yakedakevolcano-group-central-japan/. doi:10.1248/yakushi1947.107.10_780

Nazim, U. M., Jeong, J. K., and Park, S. Y. (2018). Ophiopogonin B Sensitizes TRAIL-Induced Apoptosis through Activation of Autophagy Flux and Downregulates Cellular FLICE-like Inhibitory Protein. *Oncotarget* 9, 4161–4172. doi:10.18632/oncotarget.23647

Nguyen, T. H. A., Tran, V. S., Andrea, P., Franke, K., and Wessjohann, L. A. (2003). Homoisoflavonoids from Ophiopogon Japonicus Ker-Gawler. *Phytochemistry* 62, 1153–1158. doi:10.1002/hlca.201200493

Okanishi, T., Akahori, A., Yasuda, F., Takeuchi, Y., and Iwao, T. (1975). Steroidal Sapogenins of Sixteen Liliaceae Plants. *Chem. Pharm. Bull.* 23 (3), 575–579. doi:10.1248/cpb.23.575

Park, S. H., Lee, H. J., Ryu, J., Son, K. H., Kwon, S. Y., Lee, S. K., et al. (2014). Effects of Ophiopogonin D and Spicatoside A Derived from Liriope Tuber on Secretion and Production of Mucin from Airway Epithelial Cells. *Phytomedicine* 21 (2), 172–176. doi:10.1016/j.phymed.2013.08.013

Qi, J., Hu, Z. F., Zhou, Y. F., Hu, Y. J., and Yu, B. Y. (2015). Steroidal Sapogenins and Glycosides from the Fibrous Roots of Ophiopogon Japonicus and Liriope Spicata Var. Prolifera with Anti-inflammatory Activity. *Chem. Pharm. Bull.* (Tokyo) 63 (3), 187–194. doi:10.1248/cpb.c14-00735

She, G., and Shi, J. (2003). [Structural Features of Two Neutral Polysaccharides Md-1, Md-2 from Ophiopogon Japonocus]. *Zhong Yao Cai* 26 (2), 100–101.

Shen, H. L., Xiang, N. J., Xu, Y., Gao, Q.,Miu, M. M., and Li, J. M. (2008). Analysis of Fat-Soluble Components of Ophiopogon Japonicus by GC-MS. *Chin. J. Spectrosc. Lab.* 25 (4), 669–672.

Sheng, H., Lv, W., Zhu, L., Wang, L., Wang, Z., Han, J., et al. (2020). Liriopesides B Induces Apoptosis and Cell Cycle Arrest in Human Non-small Cell Lung Cancer Cells. *Int. J. Mol. Med.* 46 (3), 1039–1050. doi:10.3892/ijmm.2020.4645

Shi, L. L., Li, Y., Wang, Y., and Feng, Y. (2015). MDG-1, an Ophiopogon Polysaccharide, Regulate Gut Microbiota in High-Fat Diet-Induced Obese C57BL/6 Mice. *Int. J. Biol. Macromol.* 81, 576–583. doi:10.1016/j.ijbiomac.2015.08.057

Sun, K., Cao, S., Pei, L., Matsuura, A., Xiang, L., and Qi, J. (2013). A Steroidal Saponin from Ophiopogon Japonicus Extends the Lifespan of Yeast via the Pathway Involved in SOD and UTH1. *Int. J. Mol. Sci.* 14 (3), 4461–4475. doi:10.3390/ijms14034461

Sun, L., Lin, S., Zhao, R., Yu, B., Yuan, S., and Zhang, L. (2010). The Saponin Monomer of dwarf Lilyturf Tuber, DT-13, Reduces Human Breast Cancer Cell Adhesion and Migration during Hypoxia via Regulation of Tissue Factor. *Biol. Pharm. Bull.* 33 (7), 1192–1198. doi:10.1248/bpb.33.1192

Tada, A., Kasai, R., Saitoh, T., and Shoji, J. (1980a). Studies on the Constituents of Ophiopogonis Tuber. V. Isolation of a Novel Class of Homoisoflavonoids and Determination of Their Structures. 1. *Chem. Pharm. Bull.* 28 (5), 1477–1484. doi:10.1248/cpb.28.1477

Tada, A., Kasai, R., Saitoh, T., and Shoji, J. (1980b). Studies on the Constituents of Ophiopogonis Tuber. VI. Structures of Homoisoflavonoids. *Chem. Pharm. Bull.* 28 (7), 2039–2044. Available at: http://www.mendeley.com/research/geology-volcanic-history-eruptive-style-yakedake-volcano-group-centraljapan/. doi:10.1248/cpb.28.2039

Tada, A., Kobayashi, M., and Shoji, J. (1973). Studies on the Constituents of Ophiopogonis Tuber. III. On the Structure of Ophiopogonin D. *Chem. Pharm. Bull.* 21 (2), 308–311. doi:10.1248/cpb.21.308

Tada, A., and Shoji, J. (1972). Studies on the Constituents of Ophiopogonis Tuber. II. On the Structure of Ophiopogonin B. *Chem. Pharm. Bull.* 20 (8), 1729–1734. Available at: http://www.mendeley.com/research/geology-volcanic-historyeruptive-style-yakedake-volcano-group-central-japan/. doi:10.1248/cpb.20.1729

Wang, H. (2013). Preventive Effects of Ophiopogon-Polysaccharide on Apiponectin in Gestational Diabetes Mellitus Rat. *Asian Pac. J. Trop. Med.* 6 (4), 296–299. doi:10.1016/S1995-7645(13)60059-0

Wang, H., Yu, H., Sun, Y., Zhao, H., Guo, Z., and Yu, B. (2017a). Liriopesides B Inhibited Cell Growth and Decreased CA125 Level in Human Ovarian Cancer A2780 Cells. *Nat. Prod. Res.* 31 (18), 2198–2202. doi:10.1080/ 14786419.2017.1320788

Wang, H. C., Chang, F. R., Huang, T. J., Kuo, C. Y., Tsai, Y. C., and Wu, C. C. (2015). (-)-Liriopein B Suppresses Breast Cancer Progression via Inhibition of Multiple Kinases. *Chem. Res. Toxicol.* 28 (5), 897–906. doi:10.1021/tx500518j

Wang, J. Z., Ye, L. M., and Chen, X. B. (2008). A New C27-Steroidal Glycoside from Ophiopogon Japonicus. *Chin. Chem. Lett.* 19 (1), 82–84. doi:10.1016/j.cclet.2007.10.055

Wang, K. W., Ju, X. Y., Zhang, L., Wang, W., and Shen, L. Q. (2012a). [A Novel C27-Steroidal Glycoside Sulfate from Liriope Graminifolia]. *Yao Xue Xue Bao* 47 (5), 619–623. doi:10.16438/j.0513-4870.2012.05.005

Wang, K. W., Zhang, H., Shen, L. Q., and Wang, W. (2011a). Novel Steroidal Saponins from Liriope Graminifolia (Linn.) Baker with Anti-tumor Activities. *Carbohydr. Res.* 346, 253–258. doi:10.1016/j.carres.2010.11.015

Wang, L., Jiang, X. L., Zhang, W. M., Li, F., Khan, A. A., Liu, X., et al. (2017b).Homoaro-cholestane, Furostane and Spirostane Saponins fromthe Tubers of Ophiopogon Japonicus. *Phytochemistry* 136, 125–132. doi:10.1016/j.phytochem.2017.01.006

Wang, L. Y., Wang, Y., Xu, D. S., Ruan, K. F., Feng, Y., and Wang, S. (2012b). MDG-1, a Polysaccharide from Ophiopogon Japonicus Exerts Hypoglycemic Effects through the PI3K/Akt Pathway in a Diabetic KKAy Mouse Model. *J. Ethnopharmacol.* 143, 347–354. doi:10.1016/j.jep.2012.06.050

Wang, S., Lin, X., Wang, L. Y., Ruan, K. F., Feng, Y., and Li, X. Y. (2012c). A Polysaccharides MDG-1 Augments Survival in the Ischemic Heart by Inducing S1P Release and S1P1 Expression. *Int. J. Biol. Macromol.* 50 (3), 734–740. doi:10.1016/j.ijbiomac.2011.12.005

Wang, S., Zhang, Z., Lin, X., Xu, D. S., Feng, Y., and Ding, K. (2010). A Polysaccharide, MDG-1, Induces S1P1 and bFGF Expression and Augments Survival and Angiogenesis in the Ischemic Heart. *Glycobiology* 20 (4), 473–484. doi:10.1093/glycob/cwp199

Wang, W. (2010). Studies on the Chemical Constituents of Liriope Graminifolia (L.) Baker. Zhejiang Gongshang University.

Wang, X. M., Sun, R. G., Zhang, J., Chen, Y. Y., and Liu, N. N. (2012d). Structure and Antioxidant Activity of Polysaccharide POJ-U1a Extracted by Ultrasound from Ophiopogon Japonicus. *Fitoterapia* 83 (8), 1576–1584. doi:10.1016/j.fitote.2012.09.005

Wang, X. M., Sun, R. G., Hao, D. P., and Wang, M. (2016). Structural Analysis and Observation of Ophiopogon Japonicus Polysaccharide POJ-U1b. Nat. Prod. Res. Deve. 28, 1228–1232.

Wang, Y., Xu, J., Zhang, L., and Qu, H. B. (2011b). A New Steroidal Glycoside from the Ophiopogon Japonicus Ker-Gawler (Liliaceae). *Nat. Prod. Res.* 25 (1), 31–35. doi:10.1080/14786411003762051

Wang, Y., Zhu, Y., Ruan, K., Wei, H., and Feng, Y. (2014). MDG-1, a Polysaccharide from Ophiopogon Japonicus, Prevents High Fat Diet-Induced Obesity and Increases Energy Expenditure in Mice. *Carbohydr. Polym.* 114, 183–189. doi:10.1016/j.carbpol.2014.08.013

Wang, Y., Li, D., Song, L., and Ding, H. (2020b). Ophiopogonin D Attenuates PM2.5-induced Inflammation via Suppressing the AMPK/NF-kappa B Pathway in Mouse Pulmonary Epithelial Cells. Exp. *Ther. Med.* 20 (6), 139. doi:10.3892/etm.2020.9268

Watanabe, Y., Sanada, S., Ida, Y., and Shoji, J. (1984). Comparative Studies on the Constituents of Ophiopogonis Tuber and its Congeners. III. Studies on the Constituents of the Subterranean Part of Ophiopogon Ohwii OKUYAMA and O. Jaburan (KUNTH) LODD. *Chem. Pharm. Bull.* 32 (10), 3994–4002. Available at: http://www.mendeley.com/research/geology-volcanic-historyeruptive-style-yakedake-volcano-group-central-japan/. doi:10.1248/cpb.32.3994

Watanabe, Y., Sanada, S., Ida, Y., and Shoji, J. (1983b). Comparative Studies on the Constituents of Ophiopogonis Tuber and its Congeners. II. Studies on the Constituents of the Subterranean Part of Ophiopogon Planiscapus Nakai. 1. *Chem. Pharm. Bull.* 31 (10), 3486–3495. doi:10.1248/cpb.31.3486

Watanabe, Y., Sanada, S., Ida, Y., and Shoji, J. (1983a). Comparative studies on the constituents of ophiopogonis tuber and its congeners. I. Studies of the constituents of the subterranean part of Liriope platyphylla Wang et Tang. *Chem. Pharm. Bull.* 31 (6), 1980–1990. doi:10.1248/cpb.31.1980

Watanabe, Y., Sanada, S., Ida, Y., and Shoji, J. (1985). Comparative Studies on the Constituents of Ophiopogonis Tuber and its Congeners. IV. Studies on the Homoisoflavonoids of the Subterranean Part of Ophiopogon Ohwii OKUYAMA and O. Jaburan (KUNTH) LODD. *Chem. Pharm. Bull.* 33 (12), 5358–5363. Available at: http://www.mendeley.com/research/geology-volcanic-historyeruptive-style-yakedake-volcano-group-central-japan/. doi:10.1248/cpb.33.5358

Watanabe, Y., Sanada, S., Tada, A., and Shoji, J. (1977). Studies on the Constituents of Ophiopogonis Tuber. IV. On the Structures of Ophiopogonin A, B', C, C', and D'. *Chem. Pharm. Bull.* 25 (11), 3049–3055. doi:10.1248/cpb.25.3049

Wu, X., Dai, H., Huang, L., Gao, X., Tsim, K. W., and Tu, P. (2006). A Fructan, from Radix Ophiopogonis, Stimulates the Proliferation of Cultured Lymphocytes: Structural and Functional Analyses. *J. Nat. Prod.* 69 (9), 1257–1260. doi:10.1021/np060033d

Wu, Y., Bi, S.-X., Huang, Z., Qi, J., and Yu, B.-Y. (2018). Novel Steroidal Saponins with Cytotoxic Activities from the Roots of Ophiopogon Japonicus (L. f.) Ker-Gawl. *RSC Adv.* 8 (5), 2498–2505. doi:10.1039/c7ra12363a

Wu, Y., Li, Y. W., Qi, J., and Yu, B. Y. (2014). Ethyl Acetate- Soluble Chemical Constituents of Fibrous Roots of Liriope Muscari. *Chin. J. Exp. Tradit. Med.* 20 (1), 163–166.

Wu, Y., Wang, X.-M., Bi, S.-X., Zhang, W., Li, R.-M., Wang, R.-J., et al. (2017). Novel Cytotoxic Steroidal Saponins from the Roots of Liriope Muscari (Decne.) L.H. Bailey. *RSC Adv.* 7 (23), 13696–13706. doi:10.1039/c6ra26031d

Xiao, Z. Q., Wang, Y. L., Gan, S. R., and Chen, J. C. (2014). Polysaccharides from Liriopes Radix Ameliorates Hyperglycemia via Various Potential Mechanisms in Diabetic Rats. *J. Sci. Food Agric.* 94, 975–982. doi:10.1002/jsfa.6347

Xiong, S. L., Hou, D. B., Huang, N., and Li, A. L. (2012). Preparation and Biological Activity of Saponin from Ophiopogon Japonicus. *Afr. J. Pharm. Pharmacol.* 6 (26), 1964–1970. doi:10.5897/ajpp12.484

Xiong, S. L., Li, A., Huang, N., Lu, F., and Hou, D. (2011). Antioxidant and Immunoregulatory Activity of Different Polysaccharide Fractions from Tuber of Ophiopogon Japonicus. *Carbohydr. Polym.* 86 (3), 1273–1280. doi:10.1016/j.carbpol.2011.06.025

Xu, J., Wang, Y., Xu, D. S., Ruan, K. F., Feng, Y., and Wang, S. (2011). Hypoglycemic Effects of MDG-1, a Polysaccharide Derived from Ophiopogon Japonicas, in the Ob/ob Mouse Model of Type 2 Diabetes Mellitus. *Int. J. Biol. Macromol.* 49, 657–662. doi:10.1016/j.ijbiomac.2011.06.026

Xu, T. H., Xu, Y. J., Xie, S. X., Zhao, H. F., Han, D., Li, Y., et al. (2008b). A Novel Steroidal Glycoside, Ophiofurospiside A from Ophiopogon Japonicus (Thunb.) Ker-Gawl. *J. Asian Nat. Prod. Res.* 10 (5), 415–418. doi:10.1080/10286020801966567

Xu, T., Xu, Y., Chen, P., Han, D., Zhao, H., Si, Y., et al. (2007). A New Furospirostanol Saponin, Ophiofurospiside B from Ophiopogon Japonicus (Thunb.) Ker-Gawl. *Chem. Res. Chin. Universities* 23 (6), 742–744.doi:10.1016/s1005-9040(07)60161-5

Xu, Y. J., Xu, T. H., Hao, L. Z., Zhao, H. F., Xie, S. X., Si, Y. S., et al. (2008a). Two New Steroidal Glucosides from Ophiopogon Japonicus (L.f.) Ker-Gawl. *Chin. Chem. Lett.* 19 (7), 825–828. doi:10.1016/j.cclet.2008.04.033

Yan, Z., Liu, G., Liang, M., and Xu, Y. (2019). Ophiopogonin D Inhibits Cell Proliferation and Induces Apoptosis of Human Laryngocarcinoma through Downregulation of Cyclin B1 and MMP-9 and Upregulation of P38-MAPK Signaling. *Oncol. Lett.* 17 (2), 1877–1882. doi:10.3892/ol.2018.9788

Yoshitama, K., Kawasoe, T., and Ishikura, N. (1993). Isolation of a New Flavonol Glycoside and its Effects on the Blue Color of Seed coats ofOphiopogon Jaburan. *J. Plant Res.* 106, 223–227. doi:10.1007/bf02344589

Yu, B.-Y., Hirai, Y., Shoji, J., and Xu, G.-J. (1990). Comparative Studies on the Constituents of Ophiopogonis Tuber and its Congeners. VI. Studies on the Constituents of the Subterranean Part of Liriope Spicata Var. Prolifera and L. Muscari. (1). *Chem. Pharm. Bull.* 38 (7), 1931–1935. doi:10.1248/cpb.38.1931

Yu, B. Y., Qiu, S. X., Zaw, K., Xu, G. J., Hirai, Y., Shoji, J., et al. (1996). Steroidal Glycosides from the Subterranean Parts of Liriope Spicata Var. Prolifera. *Phytochemistry* 43 (1), 201–206. doi:10.1016/0031-9422(96)00228-2

Zhang, H. M.,Wang, G. L., Bai, C. Q., Liu, P., Liu, Z. M., Liu, Q. Z., et al. (2011). A New Eudesmane Sesquiterpene Glucoside from Liriope Muscari Fibrous Roots. *Molecules* 16 (11), 9017–9024. doi:10.3390/molecules16119017

Zhang, J., Fan, S., Mao, Y., Ji, Y., Jin, L., Lu, J., et al. (2016a). Cardiovascular Protective Effect of Polysaccharide from Ophiopogon Japonicus in Diabetic Rats. *Int. J. Biol. Macromol.* 82, 505–513. doi:10.1016/j.ijbiomac.2015.09.069

Zhang, T., Kang, L. P., Yu, H. S., Liu, Y. X., Zhao, Y., Xiong, C. Q., et al. (2012a). Steroidal Saponins from the Tuber of Ophiopogon Japonicus. *Steroids* 77 (12), 1298–1305. doi:10.1016/j.steroids.2012.04.015

Zhang, T., Zou, P., Kang, L. P., Yu, H. S., Liu, Y. X., Song, X. B., et al. (2009). Two Novel Furostanol Saponins from Ophiopogon Japonicus. *J. Asian Nat. Prod. Res.* 11 (9), 824–831. doi:10.1080/10286020903156265

Zhang, W., Zhang, Q., Jiang, Y., Li, F., and Xin, H. (2016b). Effects of ophiopogonin B on the Proliferation and Apoptosis of SGC-7901 Human Gastric Cancer Cells. *Mol. Med. Rep.* 13, 4981–4986. doi:10.3892mmr.2016.5198

Zhang, Y., Han, Y., Zhai, K., Sun,M., Liu, J., Yu, B., et al. (2015a). Ophiopogonin-D Suppresses MDA-MB-435 Cell Adhesion and Invasion by Inhibiting Matrix Metalloproteinase-9. *Mol. Med. Rep.* 12 (1), 1493–1498. doi:10.3892/mmr.2015.3541

Zhang, Y., Liu, J., Kou, J., Yu, J., and Yu, B. (2012b). DT-13 Suppresses MDA-MB-435 Cell Adhesion and Invasion by Inhibiting MMP-2/9 via the P38 MAPK Pathway. *Mol. Med. Rep.* 6, 1121–1125. doi:10.3389/fphar.2018.0145010.3892/mmr.2012.1047

Zheng, Q., Feng, Y., Xu, D. S., Lin, X., and Chen, Y. Z. (2009). Influence of Sulfation on Anti-myocardial Ischemic Activity of Ophiopogon Japonicus Polysaccharide. *J. Asian Nat. Prod. Res.* 11 (4), 306–321. doi:10.1080/10286020902727363

Zhou, C. X., Zou, L., Mo, J. X., Wang, X. Y., Yang, B., He, Q. J., et al. (2013b). Homoisoflavonoids from Ophiopogon Japonicus. *Hca* 96 (7), 1397–1405. doi:10.1002/hlca.201200493

Zhou, Y.-F., Qi, J., Zhu, D. N., and Yu, B. Y. (2008). Homoisoflavonoids from Ophiopogon Japonicus and its Oxygen Free Radicals (OFRs) Scavenging Effects. *Chin. J. Nat. Medicines* 6 (3), 201–204. doi:10.3724/sp.j.1009.2008.00201

Zhou, Y. F., Hu, Y. Y., Mao, J. W., and Yu, B. Y. (2013a). Steroidal A glycones from Acid-Hydrolyzed Products of Ophiopogon Japonicus. *Bio Technology: An Indian Jounrnal* 8 (12), 1674–1677.

Zhu, X., Wang, K., and Chen, Y. (2020). Ophiopogonin D Suppresses TGF-β1- Mediated Metastatic Behavior of MDA-MB-231 Breast Carcinoma Cells via Regulating ITGB1/FAK/Src/AKT/β-catenin/MMP-9 Signaling axis. *Toxicol. Vitro* 69, 104973. doi:10.1016/j.tiv.2020.104973

Zhu, Y., Cong, W., Shen, L., Wei, H., Wang, Y., Wang, L., et al. (2014). Fecal Metabonomic Study of a Polysaccharide, MDG-1 from Ophiopogon Japonicus on Diabetic Mice Based on Gas Chromatography/time-Of-Flight Mass Spectrometry (GC TOF/MS). *Mol. Biosyst.* 10, 304–312. doi:10.1039/c3mb70392d

Zhu, Y., Yan, K., and Tu, G. (1987). Two Homoisoflavones from Ophiopogon Japonicus. *Phytochemistry* 26 (10), 2873–2874. doi:10.1016/S0031-9422(00)83615-8
